# Determinants of care outcomes for patients who die in hospital in Ireland: a retrospective study

**DOI:** 10.1186/s12904-015-0014-2

**Published:** 2015-04-18

**Authors:** Kieran McKeown, Trutz Haase, Jonathan Pratschke, Shelagh Twomey, Helen Donovan, Feline Engling

**Affiliations:** Social and Economic Research Consultant, 16 Hollybank Road, Dublin, Ireland; Social and Economic Consultant, Dublin, Ireland; Research Fellow, Department of Economics and Statistics, University of Salerno, Salerno, Italy; Specialist Palliative Care Nurse, Health Service Executive, Wexford, Ireland; Senior Utilization Officer, Sheikh Khalifa General Hospital, Abu Dhabi, United Arab Emirates; Research Assistant, Lisbon, Portugal

**Keywords:** End-of-life, Palliative care, Hospital deaths, Outcomes, Multi-level modelling

## Abstract

**Background:**

More people die in hospital than in any other setting which is why it is important to study the outcomes of hospital care at end of life. This study analyses what influenced outcomes in a sample of patients who died in hospital in Ireland in 2008/9. The study was undertaken as part of the Irish Hospice Foundation’s Hospice Friendly Hospitals Programme (2007–2012).

**Methods:**

Outcomes of care were assessed by nurses, doctors and relatives who cared for the patient during the last week of life. Multi-level modelling was used to analyse how care outcomes were influenced by care inputs.

**Results:**

The sample of 999 patients represents 10% of acute hospital deaths and 29% of community hospital deaths in Ireland in 2008/9. Five care outcomes were assessed for each patient: symptom experience, symptom management, patient care, acceptability of the way patient died, family support. Care outcomes during the last week of life tended to be better when: the patient had cancer; admission to hospital was planned rather than emergency; death occurred in a single room or where privacy, dignity and environment of the ward was better; team meetings were held; there was good communication with patients and relatives; relatives were facilitated to stay overnight and were present at the time of death; nursing staff were experienced and had training in end-of-life care; the hospital had specific objectives for developing end-of-life care in its service plan.

**Conclusions:**

The study shows significant differences in how care outcomes, including pain, were assessed by nurses, doctors and relatives. Care inputs operate in a mutually reinforcing manner to generate care outcomes which implies that improvements in one area are likely to have spill-over effects in others. Building on these findings, the Irish Hospice Foundation has developed an audit and review system to support quality improvement in all care settings where people die.

**Electronic supplementary material:**

The online version of this article (doi:10.1186/s12904-015-0014-2) contains supplementary material, which is available to authorized users.

## Background

The outcome which hospitals, and health services, seek to achieve is the best possible quality of life and well-being for each person and the wider population. This understanding of outcome is informed by the WHO definition of health [1], including its definition of palliative care which states: ‘Palliative care is an approach that improves the quality of life of patients and their families facing the problem associated with life-threatening illness, through the prevention and relief of suffering’ [2-4]. The importance of framing health services in terms of outcomes is widely recognised but so too are the challenges of measuring outcomes and their determinants [5]. It is normally easier to measure inputs (such as financial resources, staff, physical capital) and processes (flow of patients, admissions, discharges, waiting times) but more difficult to measure the resulting outcomes.

In Ireland more people die in hospital (43%) than in any other setting; the remainder die at home (26%), in long-stay places of care (25%), and in hospice (6%) [6]. This is broadly similar to other developed countries where the trend towards hospitalisation of dying and, more generally, ‘the medicalisation of dying’ [7,8], co-exists with the fact that most people, whether in Ireland [9], England [10] or Europe [11], prefer to die at home, including those who are terminally ill, but most will actually die in hospital [12]; in Ireland, the preference to die at home is even stronger among doctors and nurses than among the general population [13,14]. It seems likely that substantial proportions of the population in all developed countries will continue to die in hospital [15,16] which is why it is important to understand and measure the outcomes of hospital care at the end of life.

Care outcomes are defined to include dimensions of quality which have been used, either singly or in combination, in previous studies to measure end-of-life care: acceptability of the way patient died [17]; quality of patient care [18,19]; patient’s symptom experience [20]; patient’s symptom management [21]; support for family [18,19]. Care inputs are defined as the structure and process of care [5].

## Methods

The study assesses patient experiences of dying in hospital (care outcomes) and the factors associated with variations in that experience (care inputs). The patient’s experience was assessed retrospectively through the perceptions of nurses, doctors and relatives who provided most of the care during the last week of life. Full details of the methods, including questionnaires, are in the manual for the study (Additional file 1).

### Measures

#### Care outcomes

‘Acceptability’ is a global judgement about whether the patient had an acceptable death. This was measured by asking nurses, doctors, and relatives if the way the patient died was personally acceptable to them. This question was adapted from a study of dying in French hospitals [17] and the response format was changed from ‘yes/no’ to a 10-point scale ranging from ‘definitely not acceptable’ (1) to ‘very acceptable’ (10).

The quality of patient care was measured using three questions: How well did staff manage the patient’s symptoms? How well did staff communicate with the patient? How well did staff respect the patient’s wishes? These questions were developed after reviewing the domains of care used in the Family Evaluation of Hospice Care (FEHC) scale [18,19] and consulting with experts in end-of-life care to ensure face and content validity. A similar process was used to generate the two questions on family support: How well did staff communicate with relatives? How well did staff give emotional support to relatives? These questions were rated on a 10-point scale from ‘not well’ (1) to ‘very well’ (10).

The patient’s symptom experience and symptom management focused on five symptoms that are common at the end of life: pain, nausea, breathlessness, secretions and anxiety [20,21]. Symptoms were measured on a 4-point scale: ‘all the time’ (1), ‘most of the time’ (2), ‘some of the time’ (3), ‘none of the time’ (4). Symptom experience was rated on a 10-point scale from ‘unsatisfactory’ (1) to ‘satisfactory’ (10); symptom management was rated on a 10-point scale from ‘very badly’ (1) to ‘excellent’ (10). In order to keep the questionnaires manageable for respondents, data on symptom management was collected from nurses and doctors only while data on symptom experience was collected from nurses and relatives only.

#### Care inputs

Care inputs were measured by: patient characteristics including disease and whether death was sudden or expected; route of admission to hospital and length of stay; physical environment of hospital including single rooms; end-of-life care decisions including diagnosis of dying; multidisciplinary team working; communication with patients and relatives; documentation in healthcare records; support for families before, during and after death; ward and hospital culture; hospital characteristics including focus on end-of-life care.

#### Questionnaires

Six questionnaires were designed to collect the data:

1. Nurse’s Perception of Patient
2. Doctor’s Perception of Patient
3. Relative’s Perception of Patient
4. Ward Culture
5. Hospital Culture
6. Hospital Resources & Facilities

These six questionnaires were linked by a common identification code so that every item of information on a patient was linked to corresponding information about the nurse, doctor, relative, ward and hospital. Questionnaires were piloted in six hospitals before being finalised.

### Sample

#### Sample of hospitals

All the main public acute hospitals and larger community hospital were invited to participate in the study. A total of 43 hospitals participated – 24 acute and 19 community – equivalent to nearly two thirds (62%) of those invited. The study covers a major part of the acute hospital sector in Ireland as measured by the number of patients (72%), deaths (71%), staff (73%) and bed-capacity (74%). The study covers 20% of all community hospital beds in Ireland.

#### Ethical approval and consent

Ethical approval was sought and obtained from every hospital participating in the study. In some instances, approval was granted by an ethics committee which covered all hospitals in a geographical area (HSE Dublin North East; HSE South); in others, approval was granted by the ethics committee of individual hospitals (Mater Misericordiae University Hospital; Beaumont Hospital; St. James’s & Tallaght Hospital; St. Luke’s Hospital; Connolly Hospital; Naas General Hospital; Cork University Hospital; Kerry General Hospital; Limerick Regional Hospital; Sligo General Hospital; Letterkenny General Hospital). Bereaved relatives were invited by a letter from each hospital to participate in the study. This was followed up by a telephone call from each hospital to explain the study in more detail and request their consent to send out the questionnaire. Questionnaires were sent to relatives who consented to receive them and returned questionnaires were taken as evidence of implied and informed consent.

#### Sample of deaths

Each hospital selected a sample of 50 deaths in the four month period between November 2008 and February 2009. The quota (approximately 12 deaths per month for four months) was filled by taking all deaths from the beginning of the month until the quota was completed. This ensured that hospital staff could excise no influence on which deaths were selected while also ensuring the study was manageable in terms of the number of questionnaires to be completed each month. In smaller hospitals, every death was selected since it was impossible for them to meet the quota of 50 deaths in the four-month period.

### Data collection

Questionnaires 1 & 2 were completed within a week of the patient’s death. Questionnaire 3 was completed three months after the death, similar to the minimum bereavement period adopted in other surveys of bereaved relatives [22-24]. Questionnaire 4 was completed by a sample of 10 nurses and healthcare assistants in each of the wards where a patient’s death was included in the study. Questionnaire 5 was completed by a quota sample of 100 staff in each hospital with participation proportionate to five different staff categories, excluding nurses and health care assistants. Questionnaire 6 was based on 2008 data and authorised by hospital management before being returned.

### Data analysis

Multilevel modelling (MLM) was used to analyse the influence of care inputs on each care outcome since, in addition to separating individual-level (L1), ward-level (L2), and hospital-level (L3) influences, MLM also controls for covariance between the care inputs. A total of 13 multilevel models were generated by analysing the five care outcomes and the different perspectives of nurses, doctors and relatives. The model for each care outcome also contained an estimate of how that outcome was related to the other care outcomes. All models were estimated using the software package MLwiN v.2.10, and specified as three-level linear regression equations. The significance level (p < .05) was set in order to maximise the chances of identifying statistically significant influences on care outcomes. In order to build a more complete picture of these influences, ANOVA was used to test for differences between the means of each care input.

## Results

### Dataset and response rates

The sample of 999 deaths was mainly drawn from acute hospitals (880, 88%) with the remainder (119, 12%) from community hospitals. As a proportion of total deaths in Ireland in 2008, this represents 10% of acute hospital deaths and 29% of community hospital deaths, making it the largest study ever undertaken in Ireland to assess the quality of care provided to patients who die in hospital.

Data was collected from respondents (999 nurses, 737 doctors, 461 relatives, 2358 ward staff, 1858 hospital staff) on care inputs and care outcomes (Table 1). The response rate by nurses and doctors was 84% and 68% respectively (based on those hospitals which could have met the full quota of 50 deaths) which yielded a sub-sample of 737 deaths with matching data from both nurses and doctors. The response rate by relatives was 46% and within the range found in similar surveys of relatives, both in Ireland [25,26] and elsewhere [22]; this yielded a sub-sample of 461 deaths with matching data from nurses, doctors and relatives. The response rates by hospital staff to Questionnaire 4 (83%) and Questionnaire 5 (64%) were relatively high.

**Table 1:** **Dataset and response rates**

**Questionnaire**	**Dataset of completed questionnaires**	**Response rate**
1	999 deaths (completed by nurse)	84%
2	737 deaths (completed by doctor)	68%
3	461 deaths (completed by relative)	46%
4	2,358 ward staff (completed by nurse & health care assistant)	83%
5	1,858 hospital staff (completed by other hospital staff)	52%
6	24 acute & 19 community (completed by management)	100%

### Connections between care outcomes

There is a statistically significant relationship between each of the care outcomes (Table 2). This suggests that these outcomes may represent different aspects of the same underlying concept. The largest correlations are between patient care, acceptability of the way patient died and family support.

**Table 2:** **Statistically significant (p < .05) relationships between care outcomes in the assessment of nurses, doctors & relatives**

**Care outcomes**	**Symptom management**	**Symptom experience**	**Patient care**	**Acceptability**	**Family support**
Symptom management		0.44N	0.30N	0.18N	0.07N
			0.44D	0.13D	
Symptom experience			0.24N	0.26N	
			0.66R	0.23R	
Patient care				0.49N	0.58N
				0.73D	0.62D
				0.80R	0.85R
Acceptability					0.04N
					0.12D
					0.10R
Family support					

Notes: N = Nurse. D = Doctor. R = Relative. Results are unstandardised regression coefficients.

### Differences in the assessment of outcomes by nurses, doctors and relatives

Differences in how nurses, doctors and relatives assess care outcomes were measured by the level of agreement between their assessments of individual patients (Table 3). The results show that the highest level of agreement between nurses, doctors and relatives was for acceptability of the way the patient died (68%); for all the other care outcomes, the level of agreement between nurses, doctors and relatives was no greater than 25% (Table 3). There was a higher level of agreement between nurses and doctors but, for all but one care outcome, the level of agreement between them was no greater than 45%. Differences were also evident in the assessment of pain. The proportion of patients deemed to have pain ‘all or most of the time’ during the last week of life varied between doctors (11%), nurses (17%) and relatives (34%) (Table 4).

**Table 3:** **Agreement (%) between nurses, doctors & relatives on care outcomes**

**Care outcomes**	**Nurses doctors relatives %**	**Nurses & doctors %**	**Doctors & relatives %**	**Nurses & relatives %**
Acceptability of death	68	82	82	73
Patient care	19	39	39	35
Symptom experience	NA*	NA*	NA*	67
Symptom management	25	45	45	44
Support for family	25	45	45	44

Based on subset of patients (N = 312) with responses for nurses, doctors and relatives. Level of agreement was measured using a 4-point scale to minimise the risk of over-estimating the level of agreement or disagreement.

*NA = Not available because this data was collected from nurses and relatives only.

**Table 4:** **Agreement (%) between nurses, doctors & relatives on whether patient had pain all or most of the time in the last week of life**

Doctor’s rating: patient had pain all or most of the time	11%
Nurse’s rating: patient had pain all or most of the time	17%
Relative’s rating: patient had pain all or most of the time	34%
Agreement between doctors & nurses	81%
Agreement between doctors & relatives	68%
Agreement between nurses & relatives	66%
Agreement between doctors, nurses & relatives	51%

Based on subset of patients (N = 312) with responses for nurses, doctors and relatives. Level of agreement was measured using a 4-point scale to minimise the risk of over-estimating the level of agreement or disagreement.

### Rating of care outcomes

Most patients, based on the complete sample and the three sets of assessments, are reported to be relatively comfortable as far as pain (84-90%), nausea (94-95%), anxiety (87-89%), restlessness (83-85%) and chest secretions (80-83%) are concerned, but a smaller percentage are able to breathe comfortably (60-65%). Patient care, when expressed using the original 10-point scale, was 8.1 (according to doctors), 7.5 (according to nurses) and 7.3 (according to relatives). The proportion of ‘unacceptable’ deaths, scoring 3 or less out of 10, was higher in the assessment of relatives (21%) than nurses (13%) or doctors (3%). Family support, expressed using the original 10-point scale, was rated 8.3 according to nurses and doctors, and 7.0 according to relatives.

### Influence of care inputs on care outcomes

Most variation in care outcomes is explained by L1 variables with relatively little influence exercised by L2 and L3 variables (Additional file 2); this is partly due to the small sample sizes at L2 (283 wards) and L3 (43 hospitals) relative to the requirements of multi-level modelling. Eight sets of care inputs have a statistically significant influence on at least one care outcome: disease and sudden death; route of admission; physical environment; team meetings; communication; facilitating relatives; staff readiness; hospital governance.

#### Disease and sudden death

Patient care is best, in the assessment of doctors, for cancer patients (3.45 percentage points better compared to patients with circulatory diseases). The worst care, in the assessment of nurses, is for patients with dementia/frailty (−5 percentage points worse compared to patients with circulatory diseases). Patients with respiratory diseases also received lower scores from nurses on patient care (−3.16 percentage points lower than patients with circulatory diseases). The patient’s personal characteristics (age, sex, marital status, religion, ethnicity, etc.) do not influence the quality of care received although patients with private health insurance are perceived by their relatives to have a more positive symptom experience.

Nearly a quarter of all deaths in the study (24%) were sudden or unexpected. These deaths were associated with worse symptom experience according to nurses (−4.46 percentage points) and relatives (−6.94 percentage points); relatives also gave these patients a more negative appraisal of patient care (−14.57 percentage points). Further analysis revealed that sudden deaths are more likely in ED and ICU, and negatively associated with all statistically significant predictors of care outcomes.

#### Route of admission

The majority of acute hospital patients in the study were unplanned admissions through ED (84%) and this had a negative impact on care outcomes particularly in the assessment of doctors and nurses. These patients were assessed as having a less acceptable death when compared to other patients (−5.63 percentage points according to nurses and −4.13 percentage points according to doctors). In addition, these patients had more negative experience of symptoms (−5.11 percentage points according to nurses) and poorer symptom management (−4.22 percentage points according to doctors). For relatives, ED admissions are associated with a reduced sense of family support (−3.64 percentage points). Consistent with other findings, cancer patients are less likely to be admitted through ED.

#### Physical environment

Deaths in single rooms are associated with significantly better care outcomes when compared to multi-occupancy rooms. Acceptability of a patient’s death is much higher in single rooms (by 5.67 percentage points according to nurses and 5.09 percentage points according to relatives). Symptom management is better in single rooms (by 4.21 percentage points according to doctors) and symptom experience is also better (by 7.66 percentage points according to relatives). The physical environment of the room and ward (such as allowing privacy, dignity and control) had a significant influence on care outcomes (a percentage point improvement in the environment increases patient care by 0.80 percentage points for nurses and by 0.12 percentage points for doctors).

#### Team meetings

Team meetings, comprising medical and nursing staff, improve patient care (by 3.49 percentage points when assessed by doctors and 4.91 percentage points when assessed by nurses). Nurses also gave a higher rating for family support (by 2.68 percentage points) where this meeting was held. Multidisciplinary meetings, comprising all relevant health care professionals, improve symptom management by 5.22 percentage points in the assessment of nurses.

#### Communication

Each percentage point increase in the quality of discussion with patients, as assessed by nurses, improves symptom experience by 0.04 percentage points and patient care by 0.06 percentage points. Similarly, each percentage point increase in the quality of discussion with relatives, in the assessment of nurses, improves symptom management by 0.15 percentage points, patient care by 0.12 percentage points, acceptability of the patient’s death by 0.09 percentage points and family support by 0.08 percentage points. Relatives also experience an improvement in family support (0.05 percentage points) associated with the quality of discussion with relatives but the quality of discussion with patients has no effect on how relatives assess care outcomes.

#### Facilitating relatives

When a relative was present at the moment of death, the acceptability of the way the patient died increased by 5 percentage points according to both relatives and nurses. Prior to the death, when a relative stayed overnight in hospital this was associated with a beneficial impact on symptom management which improved by 3.84 percentage points when assessed by nurses.

#### Staff readiness

Staff readiness, measured by whether nurses feel professionally prepared for dealing with the death of a patient, improves how nurses and relatives assess the patient’s symptom experience (by 4.14 and 6.75 percentage points respectively). In addition, nurses who feel personally prepared for dealing with the death of a patient are more likely to see the patient’s death as acceptable (by 4.42 percentage points). The nurses’ years of experience in hospital – based on the average for the hospital – improves acceptability of the patient’s death (by 3.69 percentage points as assessed by relatives) and improves family support (by 0.91 percentage points as assessed by nurses). The patient’s symptom experience is further improved by the average number of years nurses have spent on the ward (by 0.46 percentage points in the assessment of nurses and 1.34 percentage points in the assessment of relatives). Where nurses received training since qualifying in end-of-life/palliative care, symptom management improved (by 5.92 percentage points in the assessment of doctors).

#### Hospital governance

Hospitals which have end-of-life objectives in their service plan have better symptom management (by 4.89 percentage points as assessed by doctors). Also, for each percentage point increase in the number of respondents who feel that staffing levels are insufficient, the acceptability of deaths on these wards declines (by 0.09 percentage points according to doctors).

## Discussion

The results suggest that the quality of care for people who die in Irish hospitals compares favourably to elsewhere. For example, deaths are more likely to be rated as acceptable by nurses and doctors in Ireland compared to deaths in French hospitals [17] while patients who die in Irish hospitals seem to be as comfortable as patients who die in English hospitals where the LCP has been used [21]. This is consistent with other international comparisons of palliative care [27] and consumer surveys of health services [28]. Patient care and family support are rated lower than the main comparative data which pertains to US hospices [18,19] but the fact that acute hospitals deal with a wider spectrum of deaths than hospices, from sudden to expected, would need to be taken into account in any valid comparison [29].

### Why are care outcomes better for cancer patients?

Cancer patients have better care outcomes than other patients because they are more likely to be planned admissions (though most are still unplanned), to die in a single room, to be the subject of more team meetings and better communication, to have relatives who stayed overnight and were present at the moment of death. These are statistically significant influences on care outcomes and, because directly observed by nurses, doctors and relatives, are likely to have practical clinical significance. By contrast, the quality of end-of-life care received by dementia patients is poorer because they are more likely to die in a multi-occupancy room where there is less and poorer communication and where relatives are less likely to be present at the moment of death. The study suggests that if all patients were offered the same standard of care that is currently available to cancer patients, then the quality of end-of-life care in Irish hospitals could be improved significantly.

### Route of admission to hospital

Our analysis suggests that the system of care for patients at the end of life is essentially emergency-led rather than electively-led and, within that, there are systematic variations which make certain wards (oncology and geriatric) and medical specialties (cancer) more conducive to a planned approach to the needs of patients. When these findings are added to the acknowledged under-provision of community-based and home-based services for medical needs in Ireland, and the fact that EDs are the only route of admission to hospital for most patients, it becomes clear that route of admission is symptomatic of more general problems of accessing services for patients at the end of life, with consequent knock-on impacts on overall care outcomes. Based on this study, a more planned route of admission, which would also require better coordination of services between hospital and community, could significantly improve care outcomes at the end of life.

### Communication with patients and relatives

A significant finding is that the quality of nurses’ discussion with relatives, rather than patients, was more influential in the nurses’ assessment of care outcomes. This may be due to the way communication was measured which focused on verbal communication since that may be less important than non-verbal communication as patients in the last week begin to show signs of withdrawing from the world and from contact with those around them. At the same time, previous research suggests other possible explanations for the patterns of communication identified: (i) there may be a tendency among health professionals to speak with the families of older people rather than the older person [30] (ii) hospital practitioners may have difficulty talking about dying and death [31,13] (iii) there may be a fear that relatives have a power to complain which dying patients do not [32]. Whatever the reason, these findings suggest the need for some deeper reflection by healthcare professionals on whether the patterns of communication revealed by the study are entirely consistent with a patient-centred approach and the importance of respecting the patient’s autonomy as far as possible.

### Role of training and experience

The study shows that experience and training combine to improve the readiness of nurses to care for dying patients. The effects of readiness on care outcomes are visible not only to nurses but to doctors and relatives as well. This finding draws attention to the importance of training but also of retaining experienced nurses within the hospital and ward, and ensuring they have a direct role in patient care where they can have an impact on patient outcomes and on their colleagues.

### Different perspectives of nurses, doctors and relatives

The study shows substantial differences in how nurses, doctors and relatives assess pain with correspondingly low levels of agreement between them. This suggest the assessment of pain may lack specificity (resulting in false positives) and/or sensitivity (resulting in false negatives) although it is impossible, from this data alone, to estimate the number of true positives or true negatives. The prevalence of pain (‘all or most of the time’) is lower than reported in a previous study in Ireland [25] and lower than studies of elderly patients in long-term care in Europe [33], the US [34] and Canada [35] where about 50% of the patients experienced pain in the last week of life; in roughly half of these cases, the experience of pain was a daily occurrence. This evidence, in conjunction with the diverging views of relatives, nurses and doctors on the prevalence of pain, gives some grounds for questioning how well pain is diagnosed and treated among patients who die in Irish hospitals.

### Strengths and limitations

The main strength of the study is that it is based on a large sample of hospital deaths and covers many aspects of the patient’s final journey in hospital, ‘from admission to discharge’. An additional strength is that it is based on the judgements of nurses, doctors and relatives who were with the patient during the last week of life. A significant weakness is that the patient’s voice is missing. Moreover the finding that nurses, doctors and relatives are often at variance in their assessment of care outcomes implies that their judgements may not necessarily coincide with those of patients.

It is noteworthy that hospital staff tend to give consistently higher ratings for all care outcomes compared to relatives which suggests that care outcomes may not be as good as nurses and doctors believe. It is possible that nurses and doctors assess care outcomes not just by reference to explicit standards but also use implicit standards which may be self-referential, based on limited information and may risk positive bias. There may also be a ‘study effect’ whereby hospital staff provided overly-positive ratings for care outcomes due to a sense of pride in their work, a fear of negative consequences or out of organisational loyalty.

Finally, since this is a cross-sectional study it is important to state that identification of statistically-significant effects between independent variables (care inputs) and dependent variables (care outcomes) does not necessarily imply a causal relationship.

## Conclusions

The conceptual and measurement foundations of this study have been used by the Irish Hospice Foundation to develop an audit and review system for the purpose of supporting quality improvement in all care settings where people die [36]. The main components of this system are an audit and review tool to facilitate the healthcare team in discussing and assessing the quality of end-of-life care in selected cases which is then linked to a survey of bereaved relatives.

## Additional files

Additional file 1:
**Manual for the study.**
Additional file 2:
**Statistically significant (p < .05) relationships between care inputs and care outcomes.**
