# Supplementary material for: Determinants of care outcomes for patients who die in hospital in Ireland: a retrospective study
Source: BMC Palliat Care. 2015 Apr 18;14:11. doi: 10.1186/s12904-015-0014-2 (PMC4422526; doi:10.1186/s12904-015-0014-2)
Supplement: Additional file 1: — Manual for the study. [file 12904_2015_14_MOESM1_ESM.pdf]

HospiceFriendly HOSPITALS

Putting Hospice Principles into Hospital Practice.

National Audit of End-of-Life Care in Hospitals in Ireland

2008/9 & 2011/12

The Manual

Prepared by:
Dr Kieran McKeown
Evaluation Coordinator

October 2008

The Irish Hospice Foundation
The Atlantic Philanthropies
Feidhmeannacht na Seirbhíse Sláinte
Health Service Executive

Table of Contents

ACKNOWLEDGEMENTS **III**

1. INTRODUCTION **1**

2. THE H*f*H PROGRAMME **2**

3. RATIONALE FOR NATIONAL AUDIT OF END-OF-LIFE CARE **6**

4. SCOPE OF NATIONAL AUDIT OF END-OF-LIFE CARE **9**
4.1 End-of-Life Care as a Multi-Level Reality 10
4.2 End-of-Life Care as a Multi-Faceted Reality 10
4.3 Content of Questionnaires 12
4.3.1 Questionnaire 1: Patient Profile (Nurse Version) 12
4.3.2 Questionnaire 2: Patient Profile (Doctor Version) 14
4.3.3 Questionnaire 3: Bereaved Relatives / Friends 14
4.3.4 Questionnaire 4: Ward Perceptions 14
4.3.5 Questionnaire 5: Hospital Perceptions 15
4.3.6 Questionnaire 6: Hospital Profile 15
4.4 Additional Research Modules 15

5. SAMPLE CHARACTERISTICS **16**

6. LEGAL AND ETHICAL ISSUES **21**
6.1 Legal Issues 21
6.2 Ethical Issues 23

7. IMPLEMENTATION PROCEDURES FOR THE AUDIT OF END-OF-LIFE CARE **24**
7.1 Creation of Audit Management Spreadsheet 24
7.2 Questionnaires 1 & 2: (Patient Profile - Nurse & Doctor Versions) 24
7.3 Questionnaire 3: Survey of Bereaved Relatives / Friends 25
7.4 Questionnaire 4: Survey of Ward Perceptions 26
7.5 Questionnaire 5: Survey of Hospital Perceptions 27
7.6 Questionnaire 6: Hospital Profile 28

8. DATA STORAGE **28**

9. DATA ANALYSIS **28**
9.1 Baseline Study 29
9.2 Follow-up Study 29

10. OUTPUTS OF NATIONAL AUDIT OF END-OF-LIFE CARE **30**

11. TIMEFRAME FOR IMPLEMENTING NATIONAL AUDIT END-OF-LIFE CARE 31

12. RESEARCH TEAM 31

12.1 Kieran McKeown, Evaluation Coordinator 32

12.2 Trutz Haase, Data Analyst 32

12.3 Jonathan Pratschke, Statistical Consultant 32

REFERENCES 33

APPENDIX 1: QUESTIONNAIRE 1 - PATIENT PROFILE (NURSE VERSION) 38

APPENDIX 2: QUESTIONNAIRE 2 - PATIENT PROFILE (DOCTOR VERSION) 71

APPENDIX 3: QUESTIONNAIRE 3 - BEREAVED RELATIVES 74

APPENDIX 4: QUESTIONNAIRE 4 - WARD PERCEPTIONS 93

APPENDIX 5: QUESTIONNAIRE 5 - HOSPITAL PERCEPTIONS 105

APPENDIX 6: QUESTIONNAIRE 6 - HOSPITAL PROFILE 114

APPENDIX 7: LETTER OF INVITATION TO BEREAVED RELATIVES 128

APPENDIX 8: BRIEFING NOTES FOR PHONING RELATIVES 130

APPENDIX 9: LETTER WITH QUESTIONNAIRE 133

APPENDIX 10: LEAFLET ON BEREAVEMENT 135

APPENDIX 11: THANK-YOU LETTER TO BEREAVED RELATIVES 138

APPENDIX 12: H*f*H PUBLICATION AND DISSEMINATION STRATEGY 140

Acknowledgements

This Manual is the outcome of a team effort. The contribution of the H*f*H Evaluation Sub-Committee has been truly enormous, both as individuals and as a group. The experience and expertise of its members has been invaluable in giving shape and direction to a core vision that has developed into this audit system. In addition, each member of the Evaluation Sub-Committee has brought to the work a positive and supportive style of interaction, and facilitated a process for creating and developing ideas, which has added depth and breadth to the audit system, and given it a sense of mission and ambition. The members of the Evaluation Sub-Committee are:

- Prof. David Clark — Chair, Visiting Professor of Hospice Studies, TCD & UCD
- Orla Keegan — Head of Education, Research & Bereavement Services, The Irish Hospice Foundation
- Mervyn Taylor — Programme Manager, Hospice Friendly Hospitals Programme
- Shelagh Twomey — Deputy Programme Manager, Hospice Friendly Hospitals Programme
- Max Watson — Consultant in Palliative Medicine, Northern Ireland Hospice
- Gail Birkbeck — The Atlantic Philanthropies (in attendance)

All members of the H*f*H team contributed hugely, and in a wide variety of ways, to the development of this audit system. The H*f*H team comprise the following:

- Mervyn Taylor — Programme Manager
- Shelagh Twomey — Deputy Programme Manager
- Denise Robinson — Programme Administrator
- Joanna Garcia — Support Services
- Helen Donovan — Standards Development Coordinator
- Mary Bowen — Special Projects
- Paul Murray — Development Coordinator / Special Projects
- Joanne Carr — Development Coordinator
- Ann Hayes — Development Coordinator
- Amanda Manning — Development Coordinator
- Fran McGovern — Development Coordinator
- Bryan Nolan — Development Coordinator
- Aoife O'Neil — Development Coordinator
- Ruth Agar — Development Coordinator
- Nuala Harmey — Development Coordinator
- Colette Cunningham — Development Coordinator
- Daphne Doran — Adviser on Standards Development
- Amandine Hugodot — External Consultant

Within the H*f*H team, a particular debt of gratitude is due to Mervyn Taylor, Shelagh Twomey and Helen Donovan. Their creative ideas and insights have influenced every aspect of the audit including the overall understanding of end-of-life care on which the audit is based, the linkage between standards and audit, and the detailed design of six questionnaires that

make up the audit. A national audit on the scale proposed would not have been possible without their experience and expertise of the hospital system in Ireland, and their passionate commitment to improving the quality of care and the quality of life for those people whose final days are spent in hospital.

The work of Siobhán McCarthy and Professor Ciarán O'Boyle, at the Royal College of Surgeons of Ireland (RCSI), is gratefully acknowledged. RCSI prepared a literature review on the instruments used in the bereaved relatives survey[1,] and played a lead role in the design of this questionnaire.

The National Steering Committee (NSC) has been supportive and constructive in its comments during the preparation of the audit system and this is gratefully acknowledged. The members of the National Steering Committee up to 2nd July 2008 were:

- Prof. Cillian Twomey — Chair, Consultant Physician in geriatric medicine at Cork University Hospital and St. Finbarr's Hospital, Cork
- Eugene Murray — Chief Executive Officer, The Irish Hospice Foundation
- Denis Doherty — Chairman, The Irish Hospice Foundation
- Dr. Doiminic Ó Brannagáin — Consultant in Palliative Medicine, Health Services Executive, North East
- Dr. Mary Hynes — Assistant Director, National Hospitals Office, Health Services Executive [until July 08]
- Professor David Clark — Visiting Professor of Hospice Studies, TCD & UCD
- Ann Ryan — Health Information and Quality Authority
- Sheila Dickson — President, Irish Nurses Organisation
- Julie Ling — Nurse Advisor, Services for Older People and Palliative Care, Department of Health & Children
- Orla Keegan — Head of Education, Research & Bereavement Services, Irish Hospice Foundation
- Brenda Power — Broadcaster and journalist
- Ann Kennelly — Local Health Manager, Health Services Executive, North Cork
- Margaret Murphy — Patient Advocate
- Prof. Brendan McCormack — Director of Nursing Research & Practice Development, Royal Group of Hospitals, Belfast and University of Ulster at Jordanstown
- Larry Walsh — Director of Health Services National Partnership Forum

Jonathan Pratschke, who is a member of the research team, made invaluable comments on the methodological and statistical aspects of the Manual, and contributed significantly to the questionnaire design. Trutz Haase, also a member of the research team, was very helpful at an early stage in designing the questionnaires. Karen Ryan, Consultant in Palliative Medicine at the Mater Hospital and St. Francis Hospice, was very generous with her time in commenting on earlier drafts of the questionnaires. Similarly, Professor Phil Larkin, Chair of Palliative Care Studies at University College Dublin make very insightful and constructive suggestions on earlier drafts of the questionnaires.

1 McCarthy, S. O'Boyle C., 2007.

The Manual was reviewed by a number of external experts – including Dr. Mark Dynarski from Mathematica Policy Research, Dr. Patricia Ricker from Harvard University, and Dr. Joanne Lynn from the US Office of Clinical Standards and Quality at the Centers for Medicare and Medicaid Services – and their comments and suggestions are gratefully acknowledged.

Finally, we wish to express our thanks to the funding partners of the H*f*H programme: The Atlantic Philanthropies, the Health Service Executive, the Health Services National Partnership Forum, and the Dormant Accounts Fund.

Kieran McKeown, Evaluation Coordinator - H*f*H Programme, October 2008.

1. Introduction

This Manual describes the system for a national audit of end-of-life care in Ireland. The system has been devised by, and for, the Hospice Friendly Hospitals (H*f*H) Programme. Like the H*f*H programme, this audit system is unique in being the first major initiative in an EU country to develop comprehensive standards for end-of-life care in the hospital setting, and to underpin these with a comprehensive audit system. It is essential therefore that the 'manual' or protocol for this system is fully documented and made available as widely as possible. In view of this, the Manual was written with a number of stakeholders in mind.

First, the most important stakeholder is the hospitals of Ireland, both those currently in the programme and those who may wish to join in the future. The Manual describes the rationale and scope of the audit system, the practical implications of implementing it as part of a comprehensive review of each hospital's end-of-life services, and the benefits to each hospital of adopting the system.

Second, the Manual is written for staff on the H*f*H Programme since the data yielded by the audit system will be a key resource in assisting programme implementation. Specifically, the audit system is designed to underpin the development and implementation of standards for end-of-life care in Irish hospitals, and to assist the H*f*H Development Coordinators in developing the capacity of each hospital to meet those standards.

Third, the Manual is written to enable the National Steering Committee of the H*f*H Programme to carry out its over-seeing role and ensure that programme implementation and evaluation are conducted effectively and to the highest quality.

Fourth, the Manual is written to invite and facilitate external scrutiny so that the audit system meets the highest scientific standards. This requirement is more than usually important given the ambitious and innovative nature of the audit system, the practical challenge of data collection which it entails, the analytical challenge of linking the end-of-life experiences of patients and their relatives to the caring practices and cultures of hospital systems, and the evaluation challenge of tracking change over time and assessing the impact of H*f*H in bringing about that change. For that reason, as indicated in the Acknowledgements, we invited three external experts to review the Manual.

Fifth, the Manual is designed to inform key agencies in the broader policy and regulatory environment of the H*f*H Programme – Department of Health & Children, Health Services Executive (HSE), Health Information & Quality Authority (HIQA), Oireachtas Committee on Health – about the range of data that will be generated by the audit system, and to indicate that this will be available for their use.

Sixth, and finally, we regard the audit system as a basis for collaboration with researchers in other countries, with a view to developing the system further and building a deeper comparative understanding of end-of-life care in different hospital systems. The study of end-of-life care is a relatively new area of research, and the demographics of Europe – which has the highest proportion of older citizens of any continent worldwide – suggest that European health care systems need to give greater consideration to helping people to die with dignity, especially in the hospital context, where so many deaths occur. The EU research programme in the area of health contains funding possibilities for collaborative research related to audit and evaluation systems based on the rationale that 'this research should serve to guide hospitals to develop their own effective safety and quality improvement programmes and provide the basis for assessing hospital quality of care by

purchasers and national and local governments'[2.] The audit system described in this Manual is informed by this rationale.

In practical terms, the Manual will act as the full protocol for implementing the audit system. This is essential given that the validity and reliability of the system requires a clear set of procedures, which are applied consistently in all hospitals.

2. The H*f*H Programme

The H*f*H Programme is a five year programme (2007-2012) designed to improve the quality of end-of-life care in acute and community hospitals in Ireland[3.] The programme has three aims:

- To develop comprehensive standards for all hospitals in relation to dying, death and bereavement
- To develop the capacity of acute and community hospitals[4] to introduce and sustain these standards
- To change the overall culture in hospitals and institutions in relation to dying, death and bereavement

Its activities are focused around four key themes: *Integrated Care; Communication; Design & Dignity; Patient autonomy[0].*

The rationale, or 'logic model'[5], which informs the H*f*H Programme is based on the assumption that a set of programme inputs – focused on standards and capacity development through a range of activities under the themes of integrated care, communication, dignity & design, and patient autonomy - will result in a set of outputs which are measurable against H*f*H standards for end-of-life care in hospital and which, in turn, will produce outcomes in terms of improved end-of-life experiences for patients and their families in the participating hospitals. This logic model informs the overall aim of the programme which is stated on page two of the Grant Application to The Atlantic Philanthropies as follows: "The Hospice Friendly Hospitals Programme aims to put hospice principles into hospital practice and to ensure that a systematic quality approach exists within the public health services to facilitate … a good death when it is expected, or can be predicted, and supportive systems when death occurs unexpectedly".

2 European Commission, 2008.
3 For a useful overview of the programme, see Watson, Ó Brannagáin, and Keegan, 2007.
4 There is no official definition of a 'community hospital' but the convention is to differentiate them from 'acute hospitals' if they do not have an accident and emergency department.
5 A logic model is essentially a statement of how and why a programme works. At its simplest, a logic model is built around an evidence-based theory which states that if certain inputs are used in a particular way then certain outputs and outcomes are likely to be produced. These 'if-then' statements are the causal theory on which the programme is based.

Figure 1 Logic Model of H*f*H Programme

Programme Inputs:
Develop and change the standards, capacity and culture of end-of-life care in hospitals under the four HfH themes of:
(i) Integrated care
(ii) Communication
(iii) Design&Dignity
(iv) Autonomy

→

Programme Outputs:
Improvements in the indicators which measure standards, capacity and culture of end-of-life care in hospitals under the four HfH themes.

→

Programme Outcomes:
Improvements in the in the indicators which measure patient's experience of end-of-life care in hospitals, under the HfH themes, as reported by:
(i) relatives

The H*f*H Programme is being developed in two phases. Phase One (2007-2010) focuses on hospitals which responded to a call for Expressions of Interest in 2006. Phase Two (2010-2012) will focus on hospitals likely to ensure sustainability on completion of the programme. In practice, as the programme is evolving, a steady stream of hospitals are joining the programme, or have expressed an interest in joining, and we expect this to continue as the audit system is rolled out.

The hospitals currently participating in the H*f*H Programme (at October 2008) are listed in Table 1. These comprise 18 acute hospitals (one of them for children only), equivalent to approximately a third of all (50) acute hospitals in Ireland, and range in size from 620 beds (Beaumont Hospital) to 96 beds (Monaghan General Hospital). The H*f*H Programme also comprises 21 community hospitals, equivalent to around 13% the 150 community hospitals in Ireland, and range in size from 316 beds (St. Mary's Hospital) to 50 beds (Belvilla).

Table 1 Hospitals Participating in Phase One of the H*f*H Programme, October 2008

Acute Hospitals	Community Hospitals
**Dublin: Central and South**	
• Our Lady's Children's Hospital, Crumlin	• Leopardstown Park Hospital, D18<br>• Peamount Hospital, Co. Dublin
• Adelide & Meath Hospital Incorporating National Children's Hospital - Tallaght<br>• Naas General Hospital	• Royal Hospital Donnybrook<br>• Meath Community Unit, Dublin 8<br>• Bru Chaoimhin, Dublin 8<br>• Bellvilla Community Centre, Dublin 8
**Dublin: Central and North West**	
• Mater Misericordiae University Hospital<br>• James Connolly Hospital, Blanchardstown,	• St. Mary's Hospital, Phoenix Park, Dublin
**Dublin: North**	
• Beaumont Hospital, Dublin 9	• St. Joseph's Hospital, Dublin 5<br>• Rockfield Hospital, Dublin North

North East	
• Cavan General Hospital<br>• Monaghan General Hospital<br>• Louth County Hospital, Dundalk<br>• Our Lady of Lourdes Hospital, Drogheda<br>• Our Lady's Hospital, Navan	• St. Joseph's Hospital, Ardee<br>• Boyne View, Drogheda<br>• Cottage Hospital, Drogheda<br>• St. Mary's Hospital, Drogheda<br>• St. Oliver Plunkett Hospital, Dundalk<br>• St. Joseph's Hospital, Trim<br>• St. Mary's Hospital, Castleblaney<br>• Oriel House, Monaghan<br>• Ballyconnell Unit, Cavan<br>• Lisdaran Unit, Cavan, Co. Cavan<br>• Virginia Health Care Unit, Cavan
**South East**	
• South Tipperary General Hospital, Clonmel<br>• St. Luke's General Hospital, Kilkenny<br>• Waterford Regional Hospital<br>• Wexford General Hospital	
**West**	
• Portiuncula Hospital, Ballinasloe	
**North West**	
• Sligo General Hospital	• St. John's Hospital, Sligo
**South**	
• Cork University Hospital	
**Total**	
**18**	**21**

In the period since the start-up of H*f*H in mid-2007, a substantial amount of programme activity has taken place. A staff team of 16 has been recruited comprising four in management and support services, 10 Development Coordinators, and two staff with specific responsibility for the development of standards for end-of-life care. Each Development Coordinator works with a portfolio of hospitals and, within each hospital, has facilitated the process of establishing a Standing Committee on Dying, Death and Bereavement. The Development Coordinators have adopted a 'gate-to-grave' approach in order to identify and link issues about end-of-life care across the spectrum of hospital activity and this, in turn, has assisted the Standing Committees in formulating their agenda for action to improve services. Development Coordinators have organised a public launch of the H*f*H Programme in each hospital with a view to raising awareness of the programme among hospital staff while also gaining extensive coverage for end-of-life issues through local and national media. They also keep a monthly log of their activities and significant events, so that an analysis of the unfolding history of the programme can be undertaken.

Two members of the H*f*H team work on the development of standards for end-of-life care in hospital. These standards will inform the process of change within hospitals but will also act

as the benchmark by which progress towards improved end-of-life care is measured. In February 2008, draft standards on the physical environment for end-of-life care in hospital were published by the H*f*H, and a consultation process on these standards was held between February and March 2008; the final guidelines were published in June 2008[6]. Coincidentally, in March 2008, the Health Information and Quality Authority (HIQA) published its National Quality Standards for Residential Care Settings for Older People in Ireland[7], and these also contain a standard on end-of-life care[8]. During the remainder of 2008, H*f*H staff will prepare draft standards on 'what matters most' to patients, relatives, staff, and hospital systems.

A substantial amount of research has been commissioned by the H*f*H Programme for the purpose of strengthening the knowledge-base of the programme, and providing a more solid foundation for action. In April 2007, a literature review on integrated care was carried out by the International Observatory on End of Life Care at Lancaster University[9]. In November 2007, the results of a survey carried out by Tribal Consulting on the physical design of 20 hospitals – 15 acute and 5 community – was published[10]; this publication also contained the results of a literature review on good practice in design and dignity which was carried out by the Centre for Health Policy & Management in TCD[11]. A further review, on the theme of communication in hospital settings, was completed in February 2008 by the International Observatory on End of Life Care at Lancaster University[12]; also on this theme, a communications programme has been developed to improve communications between hospital staff and patients, including piloting of a communications 'train the trainers' course[13]. Under the theme of patient autonomy, research is being carried out by University College Cork (UCC) and the Royal College of Surgeons in Ireland (RCSI) on various ethical issues surrounding end-of-life, and a public opinion survey has been carried out on this theme[14]. Two further reviews of literature were completed in the first half of 2008: one on the cost effectiveness of palliative care in hospital[15], and the other on systems used by hospitals to review deaths[16]; both reviews suggest other lines of inquiry that will be pursued as part of this audit system (see Section 4.4 below).

Within the Irish Hospice Foundation, a follow-up of the national baseline study on palliative care – published in 2006 but based on 2004 data[17] – is currently being undertaken, while the CEO of the Irish Hospice Foundation has recently published a review of progress in implementing the 2001 national palliative care strategy[18]. In June 2008, the Health Services Executive and the Irish Hospice Foundation published a report for consultation outlining a framework for integrating palliative care into the management of all chronic disease[19]. The all-systems approach to end-of-life care improvement and its evaluation has also influenced some of the thinking that has gone into the preparation of a business case for the creation of an all-Ireland Institute of Hospice and Palliative Care[20].

---

6 Hospice Friendly Hospitals Programme, 2008.
7 Health Information and Quality Authority, 2008.
8 The HIQA standard on end-of-life care states: "Each resident continues to receive care at the end of his / her life which meets his / her physical, emotional, social and spiritual needs and respects his / her dignity and autonomy" (2008:23). Thirteen criteria are then listed as indicators of this standard.
9 Cohen Fineberg and Hughes, 2007.
10 Tribal, 2007.
11 Hugodot and Normand, 2007.
12 Cohen Fineberg and Miller, 2008
13 Andec Communications, 2007.
14 Weafer and Associates Research Consultancy Ltd, 2007.
15 Hugodot and Normand, 2008.
16 Hugodot, 2008.
17 Murray, Sweeney, Smyth, and Conolly, 2006.
18 Murray, 2008.
19 Health Service Executive and the Irish Hospice Foundation, 2008
20 Prospectus, 2008.

In the broader environment of the H*f*H Programme, the reorganisation of health services continues. This has resulted in a move in 2006 from 10 health boards to a single Health Service Executive (HSE) – generating system-wide challenges, including that of finding a more appropriate balance between hospital-based and community-based services and, within hospital-based services, between acute, sub-acute and non-acute beds[21]. The scale of this challenge is acknowledged in the HSE's Transformation Programme[22], and in the review of acute hospital bed capacity published in September 2007[23]. These developments are both a challenge and an opportunity for the H*f*H Programme since they add the issue of end-of-life care to a much broader agenda of change within hospitals.

In keeping with its objectives, the H*f*H Programme has been involved in two major conferences on end-of-life care. In November 2007, a one-day conference attended by up to 500 people was addressed by distinguished national and international speakers on the four programme themes of integrated care, communication, dignity and design, and patient autonomy. The following day, a seminar on hospital design was jointly hosted by the H*f*H Programme and HSE Estates, led by Professor Roger Ulrich, before an invited audience of decision-makers in the HSE, the Department of Health & Children, and H*f*H staff; Professor Roger Ulrich also delivered a public lecture on design and dignity in Dublin in June 2008 which was attended by over 300 people. In April 2008, another major conference on end-of-life care was organised jointly by the H*f*H Programme and the National Council on Ageing and Older People (NCAOP). This conference launched a report – jointly commissioned by the H*f*H Programme and the NCAOP – on end-of-life care for older people in acute and long-stay settings, based on a postal survey of all acute and long-stay settings in Ireland[24]. In addition to national conferences, a number of seminars have been held for H*f*H staff, and facilitated by distinguished experts in various aspects of end-of-life care such as design (Ian Clarke), nursing practices (Brendan McCormack), and dying in emergency departments (Cara Bailey).

Work on developing the audit system for the H*f*H Programme began in early 2007 when the National Steering Committee appointed an Evaluation Sub-Committee. In October 2007, an Evaluation Coordinator was appointed to develop the audit system, and this Manual summarises the work carried out in the period since then.

3. Rationale for National Audit of End-of-Life Care

The audit system described in this Manual has five core objectives:

(i) To assist in implementing the H*f*H Programme, through the provision of data on end-of-life care and end-of-life experiences in hospitals;

(ii) To deepen understanding of end-of-life care and end-of-life experiences through statistical analysis of the factors associated with variations in the quality of each;

(iii) To inform the process by which hospitals assess their end-of-life care against objective standards; these standards for end-of-life care are currently being drafted by the H*f*H Programme;

(iv) To evaluate whether compliance with objective standards improves end-of-life care and end-of-life experiences in hospitals;

21 In its review of acute hospital bed capacity, PA Consulting Group (2007:15) defined sub-acute and non-acute beds as follows: 'Sub-acute beds are inpatient beds used for the continuing care of a patient requiring rehabilitation or other semi-acute services. Non-acute beds are inpatient beds used for continuing care of a patient no longer requiring acute services, eg long-term of the elderly'.
22 Health Service Executive, 2006.
23 PA Consulting Group, 2007.
24 O'Shea, Murphy, Larkin, Payne, et al, 2008.

(v) To evaluate if any change has occurred in end-of-life care and end-of-life experiences between the baseline in 2008/9 and the follow-up in 2011/12, including whether there is any difference between H*f*H and non- H*f*H hospitals, or between acute and community hospitals.

These objectives indicate that the system comprises not just audit but also the related elements of research and evaluation. Nevertheless, the predominant focus is on audit - justifying the title 'a national audit of end-of-life care' - because its main purpose is to improve end-of-life care and end-of-life experiences in hospitals[25], in line with the overall objective of the H*f*H Programme. The importance of audit as an instrument of quality improvement is clearly articulated in the report of the Commission on Patient Safety and Quality Assurance: "Clinical audit needs to be at the heart of clinical practice, and is something that all health practitioners should be engaged in. Clinical audit is about continuing evaluation and improvement by health professionals working towards delivery of safe, high quality care for patients. Clinical audit arguably constitutes the single most important method which any health care organisation can use to understand and ensure the quality of the service it provides. It is one of the principal methods used to monitor clinical quality and the results provided by clinical audit are a source of indispensable information to patients, the public, clinicians, and healthcare managers. It also provides a powerful mechanism for ongoing quality improvement highlighting incidences where standards are not met and identifying opportunities for improvement"[26].

The proposed system also contains elements of research because it seeks to identify those factors, including standards, which influence end-of-life care and end-of-life experiences since these will also to serve the overall audit purpose by contributing to the process of quality improvement. Equally, the proposed system focuses on evaluation in order to identify the extent of change over time in the quality of end-of-life care and end-of-life experiences.

In light of these considerations, it is essential that each hospital receives a detailed report on its audit results (see Section 8 below), and for the hospital's Standing Committee on Dying, Death and Bereavement, or equivalent, to use these results, in conjunction with the objective standards on end-of-life care that are currently being drafted by the H*f*H Programme, to prepare and implement a quality improvement plan for end-of-life care within the hospital.

The rationale for the audit system is also informed by a number of other considerations. First, in order to improve the quality of end-of-life care – the core objective of the H*f*H Programme – it is necessary to have a set of standards which define quality in end-of-life care, as well as valid and reliable indicators of the extent to which those standards are being met within Irish hospitals. At present, this data does not exist in any comprehensive or comparative form, partly because the standards do not exist, and partly because the study of end-of-life care is only being established as a field of study in its own right. A key instrument of change in the H*f*H Programme therefore is the development of standards covering all aspects of end-of-life care in hospitals including the physical environment, what matters most to patients, relatives, staff, and hospital systems. In order to be meaningful, these standards need to be matched and underpinned by an assessment process which allows each hospital to measure its performance against those standards. In light of this, the H*f*H Programme is simultaneously establishing a set of standards for end-of-life care and an audit system for generating baseline and follow-up data to monitor improvements in the

25 According to the National Institute of Clinical Excellence (NICE) in the UK, a clinical audit is a 'quality improvement process that seeks to improve patient care and outcomes through systematic review of care against explicit criteria and the implementation of change. Aspects of the structure, processes, and outcomes of care are selected and systematically evaluated against explicit criteria. Where indicated, changes are implemented at an individual, team, or service level and further monitoring is used to confirm improvement in healthcare delivery' (National Institute of Clinical Excellence, 2002; see also Parsley and Corrigan, 1999).
26 Commission on Patient Safety and Quality Assurance, 2008:151

quality of that care. The audit system is therefore an integral part of the H*f*H Programme, and part of the process of continuous improvement within hospitals, supporting the work of the H*f*H Development Coordinators. The data generated by the audit system, in conjunction with other sources of data and analysis within hospitals, will enable each hospital's Standing Committees on Dying, Death and Bereavement, to evaluate their performance relative to H*f*H standards, and relative to the performance of other hospitals in Ireland.

Second, the audit system has been designed to contribute to a deeper understanding of the factors which influence end-of-life care. We believe this is necessary since, in order to bring about change, it is helpful to identify the relative importance of different influences on end-of-life care, and to prioritise those areas of interventions which are likely, other things being equal, to have the biggest impact in terms of improving quality. In the audit system, we distinguish between two broad sets of factors which influence end-of-life care and end-of-life experiences: individual-level factors and hospital-level factors. Individual-level factors include the personal characteristics and symptoms of the patient, the hospital services received by the patient, the characteristics of the nurse who provided most of the during the last week of life, and the perceptions of bereaved relatives about the patient's end-of-life care. Hospital-level factors include various indicators of size and staffing levels of the hospital, hospital facilities and procedures for dying and death, as well as the hospital's and the ward's end-of-life 'culture' as perceived by staff. This multi-level approach to studying end-of-life care is similar to that found in other types of social research - such as education (where performance is seen as the outcome of student-level and classroom-level characteristics), or family well-being (where outcomes are determined by individual, family, and neighbourhood characteristics) – and lends itself to advanced statistical analysis such as multi-level modelling[27]. Given that data will be collected from all hospitals in two waves – at baseline in 2008/9 and at follow-up in 2011/12 – the audit system will yield both cross-sectional and longitudinal data, and will allow robust conclusions to be drawn about the strength and relative weight of factors associated with end-of-life care. In light of the paucity of research in this area, this promises to be a significant contribution to both the H*f*H Programme and to the study of end-of-life care generally.

Third, evaluation of the H*f*H Programme poses particular problems from the point of view of 'scientific' evaluation, especially if this is understood in terms of the 'gold standard' of experimental method[28]. This is because entry to the H*f*H Programme is through self-selection by hospitals, whereas an experimental method requires the random allocation of

27 Multi-level modelling is one of a number of statistical techniques within structural equation modelling and is essentially an advanced form of multiple regression analysis. The basic principle in a two-level model is that change in a dependent variable is the outcome of level one characteristics (in this case, the standard and experience of care by an individual patient) and level two characteristics (in this case, the profile, procedures and culture of the hospital). These characteristics can be separated into: (i) fixed parameters whose influence can be quantified using the independent variables in the dataset and (ii) variance parameters expressing the variability which cannot be explained using the existing set of independent variables. The influence of fixed parameters on a dependent variable can be separated, in turn, into an intercept (which may be interpreted as the overall adjusted mean) and a series of slopes (denoting the change in the dependent variable for a unit change in the independent variables). The advantage of multilevel modelling in the context of this audit system is that it will enable us to quantify the relative contributions of patient-level factors and hospital-level factors on the standards and experiences of end-of-life care, including the interaction effects of both levels. The statistical package to be used for multilevel modelling is called MLwiN 1.1 software, and was developed at the Institute of Education, University of London (see Rasbash, Browne, Goldstein et al., 2001).

28 The experimental method, also called a randomised control trial (RCT), involves setting up two matched groups using a random process of selection and then offering the programme to one group (usually called the experimental group) while the other group is either placed on a waiting list or offered an alternative programme (this group is usually called the control group). Since both groups are matched prior to the intervention, it is reasonable to infer that any differences which emerge at the end of the programme can be attributed to the programme. RCTs are now regarded as the gold standard among researchers for measuring the efficacy of programmes because the process of randomly allocating subjects to either an experimental or control group ensures that both groups are as perfectly matched as possible. This is because random allocation is a way of controlling for all possible differences – both known and unknown – between the groups other than the fact that one (the experimental group) receives the programme and the other (the control group) does not. Comparing the two groups before and after the programme therefore is a reliable way of assessing if the programme has any effect on the outcomes measured, since this can be the only source of differences between the two groups. In other words, it is reasonable to infer that the programme is the cause of the observed differences between the two groups. It is for this reason that RCTs provide a level of certainty about the efficacy of an intervention which is not achievable through any other research design.

hospitals to experimental and control groups. Nevertheless, a quasi-experimental method[29] – involving a comparison of H*f*H and non- H*f*H hospitals, with appropriate statistical matching and controls – offers a second-best alternative. This will make it possible to estimate the impact of H*f*H by using statistical analysis to control for the influence which baseline characteristics may exercise at follow-up and thereby estimate, other things being equal, the unique impact of the H*f*H Programme. Given that all hospitals will receive their audit results at both baseline and follow-up, the difference between H*f*H and non- H*f*H hospitals lies essentially in whether or not it has development support to promote interest and reflection on end-of-life issues; this significantly limits our capacity to assess the full impact of the H*f*H Programme, since the audit system is also part of the H*f*H Programme, and reflects the greater priority being given to audit over evaluation within the programme. The impact of the H*f*H Programme, in the restricted sense referred to, will also be assessed using qualitative analysis of the programme from an 'advocacy-for-change' perspective (see Section 4.3 below). The capacity of the study to integrate qualitative and quantitative methods is one of its strengths, giving rise to a powerful multi-method approach.

Overall, the rationale for the audit system is based on a recognition of the importance of generating data that will be useful and practical in bringing about change in hospitals, and establishing a system that may subsequently become an integral part of how hospitals monitor the quality of their end-of-life services. This, in itself, is an ambitious undertaking given that there are currently no standards for measuring end-of-life care in hospitals in Ireland, and there are few agreed definitions for many of the basic concepts, even the concept of end-of-life care itself. Nevertheless the linkage between audit and quality improvement is crucial to the rationale for the system proposed, and is also underlined in the report of the Commission on Patient Safety and Quality Assurance (January 2007-August 2008): "Clinical audit arguably constitutes the single most important method which any healthcare organisation can use to understand and ensure the quality of the service that it provides. It also provides a powerful mechanism for ongoing quality improvement"[30].

4. Scope of National Audit of End-of-Life Care

The audit system is designed to measure a comprehensive set of indicators for end-of-life care. Given that the system is also designed to enable comparison from one hospital to another, and to measure change over time, the data is collected in a standardised format; in other words, the essentially qualitative nature of end-of-life care is measured by a set of quantitative indicators. This is both a strength and a weakness and draws attention to the need to ensure that, in the interpretation of each indicator, there is appropriate consideration given to the context for that indicator, and an awareness that no one indicator, taken in isolation, is likely to give an unambiguous picture of the quality of end-of-life care.

Our understanding is that end-of-life care is both 'a multi-level' reality as well as 'a multi-faceted' reality. It is appropriate therefore to indicate how these two qualities have shaped the scope and design of the audit system.

29 A quasi-experimental method also involves setting up two groups (an experimental and comparison group) except that the process of random allocation is not used to establish each group, either because of ethical or practical considerations, or both. As a consequence, it is not possible to be sure that the two groups are perfectly matched. Nevertheless, when both groups are matched on a range of known variables – bearing in mind that, due to the absence of random allocation, there is no control over unknown variables - it is often reasonable to infer that differences between the experimental and comparison group are attributable to the programme. In this way, the quasi-experimental method is often regarded as a robust test of a programme, even if second best to a randomised control trial.
30 Commission on Patient Safety and Quality Assurance, 2008:151

4.1 End-of-Life Care as a Multi-Level Reality

The audit system is based on the understanding that end-of-life care is a multi-level reality in the sense that the experience of dying, death and bereavement within a hospital is shaped by:

(i) What happens to the patient in the last week of life. This is the 'individual-level' perspective, and is measured through the perceptions of the nurse and doctor who cared for the patient in the last week, as well as the patient's bereaved relative.

(ii) What is the environment for end-of-life care within the ward and hospital. This is the 'hospital-level' perspective, and is measured in terms of staff perceptions of end-of-life care within the ward and hospital, as well as the overall capacity of the hospital in terms of its size and facilities.

Both of these levels combine to influence the overall quality of end-of-life care, and the overall experience of dying, death and bereavement. The audit comprises six questionnaires to measure these two levels: three questionnaires at the individual level, and three at the hospital level. This is summarised in Table 2, and the questionnaires are in Appendices 1-6 of this Manual.

Table 2 Measurement Instruments in National Audit of End-of-Life Care

Level		Questionnaires
**Individual Level**	**1.1**	Questionnaire 1: Patient Profile (Nurse Version) (Green)
	**1.2**	Questionnaire 2: Patient Profile (Doctor Version) (Yellow)
	**1.3**	Questionnaire 3: Bereaved Relatives (Blue)
**Hospital Level**	**2.1**	Questionnaire 4: Ward Perceptions (Pink)
	**2.2**	Questionnaire 5: Hospital Perceptions (Purple)
	**2.3**	Questionnaire 6: Hospital Profile (Brown)

The audit system is based not just on a recognition of the multi-level nature of end-of-life care but on the fact that these levels are seamlessly merged and integrated in the actual practice and experience of end-of-life care. End-of-life care, as seen through the eyes of patients and relatives, is a different experience to end-of-life care as seen through the eyes of nurses and doctors, or the collective perceptions of ward and hospital staff. These perspectives are inextricably linked as part of the same underlying reality – and in that sense both are true - and the audit system is designed to reflect this tiered, multi-perspective reality. It is for this reason that we will merge all six questionnaires into one integrated database using a linked ID numbering system, described below (see Section 6.1).

4.2 End-of-Life Care as a Multi-Faceted Reality

End-of-life care is multi-faceted as well as being multi-level, essentially because the range of variables and indicators that are potentially relevant, directly or indirectly, to understanding this concept is vast, at both the patient-level and the hospital-level. Like all concepts, end-of-life care is measurable only through various indicators of the underlying reality which it

represents because, like all measurement, the concept or idea itself cannot be measured directly[31].

In recognition of this, the audit keeps the scope of data collection to manageable proportions, and focuses on those aspects of end-of-life care which are of most direct relevance to the H*f*H Programme and to improving the quality of care. For this reason, each questionnaire collects data which is particularly relevant to the quality of end-of-life care, particularly under the four H*f*H Programme themes of: integrated care, communication, dignity and design, and patient autonomy. The reviews of literature which have been undertaken on these themes (see Section 2 above) were carefully examined with a view to identifying key measurement domains. In addition, the questionnaires have been developed in close consultation with all H*f*H staff, but particularly those involved in developing standards. In this way, the themes and domains in the audit system are those which are of most direct relevance to end-of-life care. Correspondingly, we have excluded a wide range of issues which, although they may exercise an indirect influence on end-of-life care – at either patient-level (such as the personality characteristics or life experiences of the patient), or hospital level (such as the hospital's management style or the morale and motivation of staff) – would be burdensome to collect relative to the potential benefits in terms of greater understanding of end-of-life care. Despite these limitations, the range of data collected by the audit system is substantial, more ambitious than any previous study of end-of-life care in Ireland, and larger than most international studies in this field.

A graphic illustration of the audit system and its relationship to the H*f*H Programme is illustrated in Figure 2. This reveals how the purpose of the audit system is to generate a database of indicators on end-of-life care which can then be used to: (i) audit programme outputs and outcomes relative to H*f*H standards and to the standards in hospitals generally; (ii) analyse the influences on key output and outcome variables, notably the quality of care and quality of patient experiences; and (iii) evaluate the extent of change between baseline and follow-up in the key output and outcome variables. Figure 2 also illustrates how the results of the audit are themselves an input into the H*f*H Programme since all data and analysis generated by the system is fed back to each hospital that participates in the audit as part of a continuous process of improving quality and capacity, as well as changing the hospital culture on end-of-life care. For those hospitals which are formally part of the H*f*H Programme, Development Coordinators support this continuous improvement process but, as explained below, they also support the audit process and the dissemination of its results.

31 This understanding of measurement is reflected in the statistical term 'latent variable', a term used to refer to the underlying factor around which various indicators are statistically associated. This, in turn, is underpinned by a philosophical understanding of measurement which recognises that the essential or 'noumenal' quality at the heart of every concept - the 'thing-in-itself' - is immeasurable and is only accessible through its 'phenomenal' manifestation. The concept of end-of-life care, and the associated concepts of life and death, lend themselves particularly to this understanding of measurement, while also engendering appropriate respect for the limitations of those measurements.

Figure 2 National Audit of End-of-Life Care and its Linkage to H*f*H Programme

Create a database of indicators for end-of-life care by collecting
Patient-level data:
- ✓ Audit-of-services to patient
- ✓ Survey of patient's bereaved relative

Hospital-level data:
- ✓ Staff perceptions of hospital's end-of-life culture
- ✓ Profile and procedures of hospital for end-of-life care

Database comprises two waves of data collected at baseline (2008/9) and follow-up (2011/2).

→

Analyse database to:
1. Audit programme outputs and outcomes in each hospital relative to all hospitals.
2. Analyse influences on key output variables (notably quality of care), and on key outcome variables (notably patient experiences).
3. Evaluate changes between baseline and follow-up in key output variables (notably quality of care), and on key outcome variables (notably patient experiences).

Programme Inputs:
Develop and change the standards, capacity and culture of end-of-life care in hospitals under the four HfH themes of:
(i) Integrated care
(ii) Communication
(iii) Design
(iv) Autonomy

→

Programme Outputs:
Improvements in the indicators which measure standards, capacity and culture of end-of-life care in hospitals under the four HfH themes.

→

Programme Outcomes:
Improvements in the in the indicators which measure patient's experience of end-of-life care in hospitals, under the HfH themes, as reported by:
(i) relatives
(ii) hospital staff.

4.3 Content of Questionnaires

The six questionnaires which form the dataset for the audit were devised to measure end-of-life care and experiences under the four themes of the H*f*H Programme, which are: integrated care, communication, dignity & design, and patient autonomy[0]. Within each theme, we identified a number of measurement domains, and drew upon H*f*H guidelines and existing research to develop a set of appropriate indicators. For each questionnaire, we now set out these themes and domains; the actual indicators are the questions themselves and these are in Appendices 1-6 of this Manual, which also includes a detailed guide to each to the instruments. All questionnaires were checked by the National Adult Literacy Association to ensure the use of 'Plain English'.

4.3.1 Questionnaire 1: Patient Profile (Nurse Version); Colour code: Green

The patient profile questionnaire (nurse version) is composed mainly of single questions that do not form part of established scales (See Appendix One). This reflects the fact that there are few validated instruments for measuring end-of-life care. However we have adopted an established scale for measuring the quality of end-of-life care from Family Evaluation of Hospice Care (FEHC)[32], and we use the Quality of Dying and Death Instrument (QODD)[33] to

32 Developed by, and available from, the National Hospice and Palliative Care Organisation (NHPCO), based in Virginia in the US at: http://www.nhpco.org/i4a/pages/Index.cfm?pageid=4397. The Family Evaluation of Hospice Care (FEHC) was developed by Joan Teno and Stephen Connor at Brown University in the US (Connor, Teno, Spence and Smith, 2005) based on a previously validated scale, Toolkit After-Death Bereaved Family Member Interview (Teno, Clarridge, Casey, Edgman-Levitan and Fowler, 2001).

33 Developed by, and available from, the University of Washington End of Life Care Research Program at: http://depts.washington.edu/eolcare/instruments/index.html. The Quality of Dying and Death Instrument (QODD) was developed by Donald Patrick, Ruth Engleberg and Randall Curtis (Patrick, Engleberg and Curtis 2001) and has been validated in three studies (Curtis, Patrick, Engleberg, Norris, Asp, and Byock, 2002; Hodde, Engelberg, Treece, Steinberg, and Curtis, 2004; Mularski, Heine, Osborne, Ganzini, and Curtis, 2005).

measure the quality of life in the week before death. These two scales are also used in the survey of bereaved relatives and will allow the audit to assess the degree of congruence between the views of nurses and relatives on these two aspects of end-of-life. In addition, we ask the nurse, the doctor and the bereaved relative to rate the physical space where the patient spent most of the time during the last week of life. The links between themes in the H*f*H Programme and each domain of the patient profile part of the questionnaire are summarised in Table 3.

This questionnaires also collects data on the nurse in three domains: (i) nurse characteristics including age, sex, where brought up and whether English is the first language; attitudes to dying and death; preparedness for end-of-life care, experience of particular upset following death, and personal feelings (ii) nurse perceptions of the ward including its environment and working relationships; (iii) nurse perceptions of the hospital including its priorities and supports for end-of-life care. The links between these domains and the themes in the H*f*H Programme are also summarised in Table 3. This questionnaire will be completed at baseline using a 'pencil-and-paper' format, but it may be possible to have it completed electronically or 'on-line' at follow-up.

Table 3 Themes and Domains Covered in Questionnaire 1: Patient Profile (Nurse Version) (Green)

**Questionnaire Domain**	**Programme Themes**			
	**Integration**	**Communication**	**Design**	**Autonomy**
**Part One: Patient Profile**				
Characteristics of patient				
Place of death in hospital	□		□	
Awareness that patient was dying	□	□		□
Communication with patient	□	□	□	□
Communication with relatives	□	□	□	□
Decisions about treatment	□			□
Specialist palliative care	□			□
Quality of life in last week of life	□			□
Overall care in last week of life	□			□
Time after death	□	□		
Personal belongings of patient	□	□		
Staff review & comments on care of patient	□	□	□	□
**Part Two: Nurse's Perceptions**				
Nurse: background characteristics	□	□		
Nurse: attitudes to dying and death	□	□		
Nurse: preparation for end-of-life care	□	□		
Nurse: experiences following patient deaths	□	□		
Nurse: personal thoughts and feelings	□	□		
Ward: ward characteristics		□	□	
Ward & hospital: working environment	□	□		
Ward & hospital: quality of end-of-life care	□	□	□	□
Hospital: priorities	□	□		□
Hospital: supports for staff	□	□		

4.3.2 Questionnaire 2: Patient Profile (Doctor Version); Colour code: Yellow

This questionnaire covers a small number of domains comprising: awareness that patient was dying, decisions about treatment, specialist palliative care, and evaluation of overall care in last week of life (See Appendix Two). These domains use the same questions as in the nurse version of this questionnaire and, as such, will allow the audit to compare the perceptions of doctors and nurses on different aspects of end-of-life care.

4.3.3 Questionnaire 3: Bereaved Relatives / Friends; Colour code: Blue

The questionnaire for the survey of bereaved relatives is based primarily on questions taken from the Quality of Dying and Death Instrument (QODD)[34] (See Appendix Three). A subscale on the quality of care is taken from Family Evaluation of Hospice Care (FEHC)[35]. In addition, bereaved relatives are asked to rate the physical space where the person spent most of the time during the last week of life. In order to facilitate comparison between the perceptions of hospital staff and bereaved relatives, all of these questions are common to both questionnaires. There are also three open-ended questions which are common to both questionnaires which ask: (i) what went well in the care of this patient? (ii) what did not go so well? and (iii) what could have been done better? These open-ended questions will allow us to compare the perspectives of staff and bereaved relatives on end-of-life care, as well as offering practical guidance on how services might be improved. This coincides, once again, with the study's commitment to a multi-method approach that combines qualitative and quantitative insights. The links between programme themes and measurement domains are summarised in Table 4.

Table 4 Themes and Domains Covered in Questionnaire 3: Bereaved Relatives / Friends (Blue)

Questionnaire Domain	Programme Themes			
	Integration	Communication	Design	Autonomy
Quality of life in last week				□
Quality of care in the last week	□	□		□
Experience of the hospital and ward			□	
Relative views on care of patient	□	□	□	□

4.3.4 Questionnaire 4: Ward Perceptions; Colour code: Pink

This questionnaire covers the same domains as the nurse perceptions part of the patient profile & nurse perceptions questionnaire described above (Section 4.3.1; see Appendix Four). The ward perceptions questionnaire measures staff attitudes to dying and death, working environment, the quality of end-of-life care on the ward, personal and professional preparedness for end-of-life care, experiences of particular upset following the death of a patient as well as their perceptions of the hospital's priorities, and the quality and supports for end-of-life care. The links between these measurement domains and H*f*H Programme themes are as described in Table 3 above. The ward perceptions questionnaire is completed in each ward where a death is included in the audit, and by 10 staff in that ward - Nurses and Health Care Assistants - proportionate to their numbers on the ward.

---

34 Developed by, and available from, the University of Washington End of Life Care Research Program at: http://depts.washington.edu/eolcare/instruments/index.html. The Quality of Dying and Death Instrument (QODD) was developed by Donald Patrick, Ruth Engleberg and Randall Curtis (Patrick, Engleberg and Curtis 2001) and has been validated in three studies (Curtis, Patrick, Engleberg, Norris, Asp, and Byock, 2002; Hodde, Engelberg, Treece, Steinberg, and Curtis, 2004; Mularski, Heine, Osborne, Ganzini, and Curtis, 2005).

35 Developed by, and available from, the National Hospice and Palliative Care Organisation (NHPCO), based in Virginia in the US at: http://www.nhpco.org/i4a/pages/Index.cfm?pageid=4397. The Family Evaluation of Hospice Care (FEHC) was developed by Joan Teno and Stephen Connor at Brown University in the US (Connor, Teno, Spence and Smith, 2005) based on a previously validated scale, Toolkit After-Death Bereaved Family Member Interview (Teno, Clarridge, Casey, Edgman-Levitan and Fowler, 2001).

4.3.5 Questionnaire 5: Hospital Perceptions; Colour code: Purple

This questionnaire is a sub-section of the Questionnaire 4 - Ward Perceptions (See Appendix Five). It measures staff attitudes to dying and death, their preparedness for end-of-life care, their experience of particular upset following the death of a patient as well as their perceptions of the hospital's priorities, its working environment, and the quality and supports for end-of-life care. The links between programme themes and measurement domains are as described in Table 3 above. The survey of hospital perceptions will be completed by 100 staff across all categories in the hospital, excluding nurses and health care assistants who are already included in the survey of ward perceptions. The sample will reflect the overall distribution of staff in the hospital, subject to the requirement that at least one staff must be selected from each of the following in order to ensure that numerically small staff categories are also represented: CEO / General manager, nursing management (DoN, Ass DoN, CNM3), consultant doctors, non-consultant doctors, allied health professionals, pastoral carers, bereavement coordinators, end-of-life care coordinators, porters, catering staff, household staff, mortuary staff, security staff, complaints officer / patient advice & liaison officer, ward clerk, reception, administration. The data from this questionnaire will be merged with matching data from the Ward Perceptions Questionnaire to give an overall measure of hospital culture on end-of-life care.

4.3.6 Questionnaire 6: Hospital Profile; Colour code: Brown

This questionnaire uses a range of measures to assess the hospital's size, capacity and structure, as measured by the number of beds, patients, deaths and staff (See Appendix Six). Informed by the H*f*H Design & Dignity Guidelines for the Physical Environments of Hospitals Providing End-of-Life Care[36], the questionnaire also measures the range of hospital facilities, procedures, and resources for end-of-life care, including characteristics of the mortuary. As such, the questionnaire covers all the H*f*H Programme themes, and generates 'hard data' on hospital-level characteristics. This questionnaire is completed and verified by hospital management.

4.4 Additional Research Modules

In addition to the quantitative data collected through the audit system, we will also carry out additional, more qualitative research. The full scope of this work has still to be worked out but three modules have been considered by the Evaluation Sub-Committee. These modules could form the basis of PhD theses, with the possibility of supervision being provided by appropriate members of the National Steering Committee and its sub-committees.

The first module would involve case studies of end-of-life journeys based on interviews with actual patients, particularly since all of the data in the audit system are based on the perceptions of either relatives or staff, rather than the patients themselves. The limited focus of the audit system, while understandable and in line with the methodologies generally used in end-of-life studies, adds cogency to the case for giving a direct voice to the patient in the overall evaluation.

The second module would involve an analysis of the H*f*H Programme from an advocacy-for-change perspective, drawing on instruments used in other advocacy evaluation studies[37]. This module could include documenting the activities of Development Coordinators and assessing the effectiveness of different change-strategies; reviewing the role of Standing Committees on Dying, Death and Bereavement and their impact on changing end-of-life care; examining the possible influence of H*f*H conferences and seminars in changing the attitudes to end-of-life care among policy makers and service providers; assessing the

36 Hospice Friendly Hospitals Programme, 2008

37 There is an online clearing house with a wide range of tools, frameworks, articles etc. on evaluating advocacy at: http://www.innonet.org; see also the website of the Irish Evaluation Network, based at DCU, at: http://www.dcu.ie/education_studies/ien/resources.htm

extent of media coverage of H*f*H and its impact on public attitudes; undertaking a retrospective identification and analysis of critical incidents that have occurred since the inception of the programme, and situating the change process in the national context of public policy as articulated by the Department of Health & Children and implemented by bodies such as the HSE, and HIQA.

The third module would involve a wider systems-level review of deaths in hospitals. This would be done by linking data from the audit system to other forms of clinical and administrative data within each hospital. For example, data on the use of tests, imaging, medicines, and other hospital procedures in the period immediately preceding death could cast light on how these interventions affect the quality of living and dying for a sample of patients. In line with research carried out elsewhere[38], a case study approach could be used to review selected deaths, and this review could also include an assessment of the costs and benefits of end-of-life care in circumstances where there is variation in the intensity or aggressiveness of treatment offered by the hospital, and in the extent of structured planning by the hospital for end-of-life care[39].

5. Sample Characteristics

The H*f*H Programme has been adopted in 18 acute hospitals, equivalent to approximately one third of all (50) acute hospitals in Ireland; it is also in 21 community hospitals, equivalent to just over a tenth (13%) of all (150) community hospitals in Ireland (see Section 2 above). The ultimate ambition of the H*f*H programme is to make every hospital in Ireland – both acute and community – a ‘Hospice Friendly Hospital’. In order to facilitate this goal, and to ensure that the national audit of end-of-life care makes the largest possible impact, it was decided to offer the audit to a majority of acute hospitals; in July 2008, 40 of these hospitals were invited to participate irrespective of participation in the programme. All community hospitals (21) participating in the programme at that time were also invited to participate, as well as St. Columba’s Hospital, Thomastown, Co. Kilkenny. Given that 74% deaths in Ireland occur outside the home[40] – in either hospital or HSE facilities (76%), or in nursing homes (24%)[41] – the decision to include the largest acute hospitals in the audit will yield a fairly comprehensive picture of end-of-life care in hospitals in Ireland.

The number of deaths in acute hospitals in 2006, including the monthly average, is summarised in Table 5. This reveals that if each hospital carried out an audit of 50 deaths then, over a four month period, this would capture 91% of all deaths in the acute hospital sector in that period. In light of this, it was decided that a four month audit period would safely yield a comprehensive picture of the majority of deaths in the majority of acute hospitals.

Table 5 Deaths in Acute Hospitals in 2006, Based on HIPE Data, Including Estimated Time Required to Audit 50 Consecutive Deaths in Each Acute Hospital

Number	Hospital	Total Deaths	Average per Month
1	Beaumont Hospital	861	72
2	St. James's Hospital	840	70

38 For a review, see Hugodot and Normand, 2007.
39 Hugodot, 2008.
40 Central Statistics Office, 2006, Table 3.16; further details of the breakdown were given in a personal communication with the Vital Statistics Section of the Central Statistics Office.
41 A recent report estimated that there are 785 nursing homes – or long-stay facilities – in Ireland (O’Shea, Murphy, Larkin, Payne, et al, 2008).

Number	Hospital	Total Deaths	Average per Month
3	Mater Misericordiae University	710	59
4	Cork University Hospital	649	54
5	St. Vincent's Hospital, Elm Park	646	54
6	University College Hospital, Galway	543	45
7	Limerick Regional Hospital	515	43
8	Waterford Regional Hospital	504	42
9	Tallaght Hospital Hospital	382	32
10	Mayo General Hospital	367	31
11	Mercy University, Cork	338	28
12	Tralee General Hospital	336	28
13	Sligo General Hospital	327	27
14	Letterkenny General Hospital	320	27
15	James Connolly Memorial Hospital	292	24
16	Wexford General Hospital	292	24
17	Our Lady's Hospice, HX	276	23
18	St. Luke's Hospital, Kilkenny	262	22
19	Tullamore General Hospital	257	21
20	Loughlinstown Hospital	256	21
21	Our Lady of Lourdes Hospital, Drogheda	255	21
22	Cavan General Hospital	226	19
23	Naas General Hospital	223	19
24	South Infirmary - Victoria, Cork	206	17
25	Portiuncula Hospital, Ballinasloe	191	16
26	Louth County, Dundalk	182	15
27	Ennis General Hospital	173	14
28	Longford Westmeath General	170	14
29	Merlin Park Hospital	168	14
30	St. Joseph's Hospital, Clonmel	164	14
31	Nenagh General Hospital	152	13
32	Roscommon County Hospital	149	12
33	Our Lady's Hospital, Navan	143	12
34	Portlaoise General Hospital	117	10
35	Monaghan General Hospital	112	9
36	Mallow General Hospital	107	9
37	St. Michael's, Dun Laoghaire	91	8
38	St. John's Hospital, Limerick	67	6
39	St. Luke's Hospital, Rathgar	62	5
40	Crumlin Children's Hospital	60	5
41	St. Finbarr's Hospital, Cork	45	4

Number	Hospital	Total Deaths	Average per Month
42	Our Lady's Hospital, Cashel	29	2
43	Rotunda Hospital	22	2
44	Temple Street Children's Hospital	21	2
45	Coombe Women's Hospital	19	2
46	NMH Holles Street	18	2
47	Peamount Hospital	11	1
48	Limerick Maternity Hospital	10	1
49	Erinville Maternity Hospital	7	1
50	St. Mary's Orthopedic Hospital, Cork	3	0
51	Royal Victoria Eye & Ear Hospital	1	0
	**TOTAL**	**12,177**	**1,015**

Source: Based on Hospital In-Patient Enquiry (HIPE).

Within each hospital, the national audit of end-of-life care is seeking the following samples:

(i) Questionnaires 1 and 2: Patient Profile (Nurse and Doctor Versions). This will be completed on 50 deaths over a four month period. This will be completed as soon as possible after the patient has died, preferably within one week of the death. In hospitals where the annual average number of deaths exceeds 150, and in order to distribute evenly the burden of completing these questionnaires over the audit period, the H*f*H Programme will set a quota of deaths to be completed during each of the four months in the audit period to reflect the proportion of deaths in the previous years in: (i) A & E (ii) intensive care and (iii) other wards; these quotas will be completed by taking all consecutive deaths from the beginning of each month until the quota for that month is reached. In hospitals where the annual average number of deaths is less that 150, the questionnaires will be completed on each consecutive death within the audit period. This part of the audit lasts for four months, irrespective of whether the hospital has reached the target of 50 deaths.

(ii) Questionnaire 3: Bereaved Relative. The bereaved relative of each patient included in the audit will be contacted three months after the death in order to seek their consent to participate in a survey about the patient's experiences during the last week of life. The decision to contact relatives three months after death is informed by the practice used in the largest study ever undertaken of bereaved relatives - carried out by the US National Hospice and Palliative Care Organisation (NHPCO) - involving over 1,000 hospices and nearly 250,000 bereaved relatives in which the contact was made between 1-3 months after the death[42]. In line with similar surveys[43], we expect to receive at least 25 valid responses, based on a response rate of 50%.

42 Rhodes, Mitchell, Miller, Connor, and Teno, 2008; Teno, Janet, Shu, Casarett, Spence, Rhodes, and Connor, 2007.

43 In the US in 2005, the National Hospice and Palliative Care Organisation (NHPCO) reported an average response rate of 40% to postal surveys carried out by 695 hospices of bereaved relatives using the Family Evaluation of Hospice Care (FEHC) instrument (http://www.nhpco.org/files/public/FEHCSampleReportOct2005.pdf). Another study using the FEHC instrument, based on 631 hospices and 106,514 bereaved relatives, reported a response rate of 45% (Teno, Janet, Shu, Casarett, Spence, Rhodes, and Connor, 2007:121). In the original validation of the FEHC instrument, based on 156 bereaved relatives, the response rate was 63% (Teno, Clarridge, Casey, Edgman-Levitan, and Fowler, 2001:754-5). A survey of 935 bereaved relatives, based on interviews using the Quality of Death and Dying (QODD) instrument encountered a refusal rate of 47% (Curtis, Patrick, Engelberg, Norris, Asp, & Byock, 2002:19). An even higher refusal rate of 65% was experienced in another interview-based survey of bereaved relatives following 108 deaths, based on the QODD instrument (Mularski, Heine, Osborne, Ganzini, & Curtis, 2005:283). Significantly, a randomised control trial using the VOICES instrument to

(iii) Questionnaire 4: Ward Perceptions. This survey will be completed by 10 nurses and health care assistants in wards where a death has been included in the audit. The number of nurses and health care assistants in the sample will reflect their proportion within the ward. The names of the nurses to be included in the sample will be drawn at random from the list of all nurses working on that ward, excluding those employed on an agency basis. Similarly, the names of the health care assistants to complete the ward perceptions questionnaire will be selected at random from the list of all health care assistants working on that ward, excluding those employed on an agency basis.

(iv) Questionnaire 5: Hospital Perceptions. This survey will be completed by 100 staff across all categories in the hospital, excluding nurses and health care assistants who are already included in the survey of ward perceptions. The sample will reflect the overall distribution of staff in the hospital, subject to the requirement that at least one staff must be selected from each of the following staff categories in order to ensure that numerically small categories are also represented: general manager, nursing management (DoN, Ass DoN, CNM3s), consultant doctors, non-consultant doctors, allied health professional, social work, pastoral care, bereavement coordinator, portering, catering, household, mortuary, security, complaints officer / patient advice & liaison officer, ward clerk, reception, administration (excluding complaints officer / patient advice & liaison officer, ward clerk, and reception). The quota for each category of staff will be set by the H*f*H Programme on the basis of information supplied by the hospital in the Hospital Profile Questionnaire. Ideally, the quota for each category of staff should be filled by randomly selecting names from a list of all staff in each category. Where this is not possible, the questionnaire should be distributed as widely as possible among that staff category until the quota is met.

(v) Questionnaire 6: Hospital Profile. This questionnaire will be completed by management of the hospital, based on data for 2008.

The overall sample resulting from these requirements is summarised in Table 6. The size and structure of the sample is shaped by the multi-level and multi-faceted nature of end-of-life care, and by the need for a critical mass of hospitals to participate in order to make it a truly national audit. In addition to these audit requirements, the sample also reflects the research objective of identifying the factors which influence the quality of end-of-life care (the output) and the quality of end-of-life experience (the outcome), and the data implications of using multi-level modelling to separate individual-level and hospital-level influences (see Figures 1 and 2 above). The sample is also shaped by the evaluation objective of assessing whether, when the baseline in 2008/9 is compared with the follow-up in 2010/11, there are measurable differences in outputs and outcomes between H*f*H and non-H*f*H hospitals. In view of this, it is worth outlining in more detail why this sample size will facilitate the research and evaluation objectives of the study as well as its audit objectives.

measure the experience of bereaved relatives found that the response rate did not differ significantly between postal and interview groups [(interview: 56% (69 of 123) and postal: 52% (161 of 308)]. Although responders in the two groups did not differ in terms of their socio-demographic characteristics, postal questionnaires had significantly more missing data, particularly on questions about service provision and satisfaction with services (Addington-Hall, Walker, Jones, Karlsen, and McCarthy, 1998).

Table 6 Size of Samples for National Audit of End-of-Life Care

Measurement Instrument		Acute Hospitals (Total hospitals = 30)	Community Hospitals (Total hospitals = 20)	Total
**1.**	Patient Profile Questionnaire (Nurse Version)	50 nurses x 30 hospitals<br>Total = 1,500	10 nurses x 20 hospitals<br>Total patients = 200	Total = 1,700
**2.**	Patient Profile Questionnaire (Doctor Version)	50 doctors x 30 hospitals<br>Total = 1,500	10 doctors x 20 hospitals<br>Total patients = 200	Total = 1,700
**3.**	Bereaved Relatives Questionnaire	25 relatives x 30 hospitals<br>Total relatives = 750	5 relatives x 20 hospitals<br>Total relatives = 100	Total = 850
**4.**	Ward Perceptions Questionnaire	10 staff in 5 wards (estimated) x 30 hospitals<br>Total staff = 1,500	10 staff in 5 wards (estimated) x 20 hospitals<br>Total staff = 1,000	Total = 2,500
**5.**	Hospital Perceptions Questionnaire	100 staff x 30 hospitals<br>Total staff = 3,000	100 staff x 20 hospitals<br>Total staff = 2,000	Total = 5,000
**6.**	Hospital Profile Questionnaire	Total hospitals = 30	Total hospitals = 20	Total = 50
**Total questionnaires**		**Total = 8,280**	**Total = 3,520**	**Total = 11,800**

Sample size, from the perspective of research and evaluation, is typically informed by three considerations. First, the sample must be adequate to analyse the influence of individual-level and hospital-level characteristics on end-of-life care, as we expect significant differences to emerge at the hospital level. The adoption of a clustered, multistage sampling strategy is informed by this requirement. The resulting data (individual observations nested within hospitals) will be analysed using multi-level modelling techniques (see footnote above), which require roughly 25 individual-level units of analysis[44]. For this reason, we have set a target of 25 responses per acute hospital from the survey of bereaved relatives. Community hospitals are much smaller in size – and have significantly fewer deaths compared to acute hospitals – but all possible steps will be taken in order to include them within the multi-level analysis.

Second, the sample size must be adequate to correctly detect any change in end-of-life care between baseline and follow-up, and between H*f*H and non-H*f*H hospitals. This, in turn, is informed by four main factors. The first factor is the power of statistical tests, and the convention in robust evaluations is that any test should allow differences to be detected in 80% of cases (referred to as statistical power of 0.8)[45]. The second factor is the level of statistical significance used to evaluate the results of statistical tests. The convention is that results should be true in 95% of cases (referred to as p=0.05)[46]. The third factor is the expected size of the change between baseline and follow-up, or between H*f*H and non-H*f*H hospitals. This is more difficult to estimate, since the H*f*H Programme is a relatively unique programme and there are no precedents for its likely effect size. As a result, a cautious

44 In this study, the individual-level units comprise the patient, the nurse who provided most of the care to the patient in the last week of life, and the bereaved relative.
45 Conversely, this convention accepts the likelihood that differences between the experimental and control group may not be detected in 20% of cases.
46 Conversely, this convention accepts that differences detected between an experimental and control group may not be true in 5% of cases.

approach would suggest that the sample size should be able to detect relatively small changes, across a wide range of outcome domains[47]. Finally, the hierarchical "nesting" of individual outcomes within hospitals has consequences for the size of the sample, as there is effectively a distinct sample at each level in the hierarchy (individuals, hospitals etc.).

Power depends on the parameter being tested and, as Snijders (2005) points out, power considerations are different depending on whether the researcher focuses on testing a regression coefficient, a variance parameter, or the means for particular groups. In most studies, attention is paid to the level 1 and level 2 regression coefficients, as these provide the most powerful insights and statistical tests. In this context, it is known that sample size at level 2 (hospitals) is more important to statistical power than that at level 1 (individuals). It is therefore appropriate that the proposed sample of hospitals (50) is larger than the sample of patients and relatives within each hospital (roughly 25).

The identification of the minimum sample size, based on these considerations, is a complex task. To start with, a sample size of just over 800 would be necessary in order to conduct an *F*-test for a classical multiple regression model with 10 predictors, assuming statistical power of 80%, the significance level mentioned earlier (p=0.05), and a "small" effect size between baseline and follow-up (equivalent to 0.02 using Cohen's f^2). The sample of 1,700 estimated in Table 6 above will clearly facilitate this. If we now consider the nested design of the study, we must deal with two different sample sizes: the sample size of the micro-units (individuals) within each hospital (n) and the number of hospitals (N), with N × n being the total sample size for the micro-units. For hierarchically structured, multi-level data, where the hypothesis tested refers to one of the 'fixed' regression coefficients in the model, the formulae for the standard errors and sample sizes are provided by Snijders & Bosker (1993). These formulae are rather complex, and detailed information is needed on the means, variances and covariances of the predictor variables, as well as on the variances and covariances of the random effects included in the multi-level model. In the absence of such information, which can only be derived from prior research, we might hypothesise that the impact of the shared variance at hospital level on the effect size could be equivalent to reducing this effect size (measured by Cohen's f^2) to roughly 0.015. In this case, the required sample size would be just under 1,100. Again, the sample of 1,700 estimated in Table 6 above will clearly facilitate this.

6. Legal and Ethical Issues

In order to implement the national audit, it is essential to clarify and resolve all legal and ethical issues that may arise in carrying out a national audit of end-of-life care, particularly as it affects the rights of patients, relatives, and staff. That is the purpose of this section.

6.1 Legal Issues

The core legal issue is that the right of patients and their relatives to privacy and confidentiality must be respected at every stage of the audit. This right is enshrined in the Data Protection Acts 1988 and 2003, and it is the responsibility of the Data Protection Commissioner to uphold this right. For this reason, we have liaised closely with the Office of the Data Protection Commissioner in preparing this Manual, and clarified that our procedures are compliant with all data protection requirements.

47 The effect size is a simple way of standardising and comparing the difference between two sets of scores. It is typically used to compare the difference between an experimental and a control group, but can equally be used to compare changes within a group between baseline and follow-up. The index established by Jacob Cohen (1988) and referred to as 'Cohen's d', is calculated by subtracting the mean at baseline from the mean at follow-up and dividing by their pooled standard deviation. Thus, the effect size is measured in standard deviation units and the score varies from 0.0 to 3.0. By convention, a coefficient between 0.2 and 0.5 indicates a small change, between 0.5 and 0.8 indicates a moderate change, and above 0.8 indicates a large change.

A core requirement of the data protection legislation is that a person's 'personal data' or 'personal health information' can only be used by the hospital for the purposes for which it was collected, and can only be used for other purposes with the patient's consent. The implications of this are spelt out in the Data Protection Commissioner's Guidelines on research in the health sector as follows: "If any proposed use of a patient's data for purposes unrelated to their treatment would likely come as a surprise to them, then a new and separate consent should be sought"[48]. The same restriction applies to the contact details of relatives which are normally only collected for the purpose of contacting those relatives.

One of the 'exceptions' to this general rule is a clinical audit of the hospital's services, where this is carried out by staff members of the hospital in order 'to improve the quality of care provided to patients generally'. Explicit consent is not required in this case because, according to the Data Protection Commissioner's Guidelines: 'Given the fundamental role played by clinical audit in patient care, implied consent is normally all that is required when the audit could likely be of benefit to that patient. Implied consent will also be considered as sufficient in those cases where no direct benefit is likely to accrue to the patient concerned and where the audit is to be carried out by the health facility itself"[49].

The national audit of end-of-life care is not a 'clinical audit', as the term is used in the previous paragraph, because staff in the H*f*H Programme are not normally hospital staff. For this reason, any legal access to the personal data of patients and relatives can only take place through either: (i) obtaining the consent of patients and relatives, or (ii) anonymising the data so that it is no longer 'personal data' or 'personal health information' under the meaning of data protection legislation. In this audit, we have opted for the anonymisation route by devising an ID numbering system for all questionnaires in the national audit of end-of-life care. This protects the right to privacy of patients and relatives because: "Where patient data is anonymised, there is no need from a data protection perspective to seek the consent of patients for the use of the data for research and clinical audit purposes"[50]. For this reason, the anonymisation route has been described as 'the most desirable option' for research involving patients and relatives[51].

Strictly speaking, all data pertaining to patients and relatives in the national audit of end-of-life care is pseudo-anonymised, rather than irrevocably anonymised, since the hospital retains the coding frame which links the ID numbers to individual patients and relatives, including their contact details. Nevertheless, according the guidelines issued by the Data Protection Commissioner for research in the health sector, "the requirement to capture consent to use the data for research purposes, in such circumstances, will no longer apply"[52]. Each hospital retains the coding frame which links ID numbers to named patients and relatives, and confidentiality is protected by each hospital's existing confidentiality procedures. No one outside the hospital will have access to this coding frame.

The ID numbering system being used in the audit is indicated in Table 7. The effect of this ID numbering system, from a data protection perspective, is to anonymise the data so that it can no longer be regarded as 'personal data', and therefore does not affect the personal or privacy rights of patients or their relatives. From a statistical perspective, as explained in Section 8 below, this ID numbering system allows all of the questionnaires to be systematically linked in the analysis.

48 Data Protection Commissioner, 2007:7
49 Data Protection Commissioner, 2007:12
50 Data Protection Commissioner, 2007:12
51 Sheikh, 2008:43; see also Department of Health and Children, 2008.
52 Data Protection Commissioner, 2007:10

Table 7 ID Numbering System for Questionnaires in National Audit of End-of-Life Care

Hospital ID	Ward ID	Patient ID*
This number will be allocated to each hospital by the H*f*H Programme, and will remain confidential to each hospital.	This number (from one onwards) will be allocated by the audit manager in each hospital, with a separate number allocated to each ward in the audit.	This number (from one onwards) will be allocated by the audit manager in each hospital, with a separate number allocated to each patient in the audit.

*The Patient ID is replaced by the Questionnaire ID in Questionnaire 4: Ward Perceptions and Questionnaire 5: Hospital Perceptions.

The survey of bereaved relatives (Questionnaire 3) also raises data protection issues, since it is not permissible for the hospital to pass the contact details of bereaved relatives to a third party, such as the H*f*H Programme, without the consent of those relatives, given that those contact details were not collected for the purpose of carrying out a survey. In order to meet this requirement, and to ensure that the hospital is fully involved in the survey of bereaved relatives, we have devised a four-step procedure, described in Section 6.4 below, which has been approved by the Office of the Data Protection Commissioner, and which fully respects the rights of relatives to both privacy and confidentiality and confidentiality on the one hand, and to give or withhold consent on the other.

6.2 Ethical Issues

The core ethical issue for the audit is that bereaved relatives can only be surveyed after they have been given an opportunity to give their free and informed consent. The ethical basis for this is that every person – and not just bereaved relatives – has the right of autonomy and that includes the right to decide what s/he wants to do. As such, the requirement to seek the consent of bereaved relatives is not qualitatively different to the ethical obligation on hospitals to obtain the consent of patients to carry out a procedure or treatment[53].

Obtaining consent has been described as ‘a process – not an administrative task’[54] and, once given, it can be revoked by the person at any time. In light of this, the audit has devised a two stage process for obtaining consent. The first involves writing to the bereaved relative informing them about the study and inviting their participation; the second involves phoning the bereaved relative and giving him / her an opportunity to discuss the survey before deciding whether to give or withhold consent. In both the letter and the phone call, bereaved relatives are also informed that, should they give consent, they are free to withdraw it at any time, even after they have agreed to receive the questionnaire. This procedure, or some variant of it, is the accepted ethical practice for carrying out surveys of bereaved relatives.

In preparation for the audit, the H*f*H Programme commissioned the Royal College of Surgeons of Ireland (RCSI) to review all existing research on surveys of bereaved relatives and to develop a methodology for this part of the audit[55]. The model proposed by the RCSI, which is similar to the one proposed here, has been approved by five separate ethical committees:

- ✓ HSE Dublin / Mid-Leinster Research Ethics Committee (for Mullingar General Hospital)
- ✓ HSE South East Research Ethics Committee (for St. Columba’s Community Hospital)
- ✓ Sligo General Hospital Research Ethics Committee (for St. John’s Community Hospital)
- ✓ Naas General Hospital Research Ethics Committee (for Naas General Hospital)

53 Dublin Hospitals Group Risk Management Forum, 2006.
54 Dublin Hospitals Group Risk Management Forum, 2006:2
55 McCarthy, S. O’Boyle C., 2007.

✓ RCSI Research Ethics Committee.

In addition to these approvals, an expedited review and approval for the entire audit system has been granted by the HSE North East Area Research Ethics Committee in respect of Cavan General Hospital.

Each hospital will decide if the audit system requires ethical approval from its local research ethics committee. This is because there is no centralised system in Ireland for obtaining global ethical approval for a national audit such as this. Where ethical approval is indicated by a hospital as being necessary, the H*f*H Evaluation Coordinator will make the application and supply all the necessary documentation.

7. Implementation Procedures for the Audit of End-of-Life Care

It is essential to have clear procedures in place, embedded within each hospital's line-management structures, to ensure that the audit is administered in a uniform and consistent manner across all hospitals. A core requirement therefore is that each hospital will appoint an audit manager to oversee the entire audit. This is not a full-time position, but it will require a person of experience and authority within the hospital to manage the process. This section outlines the set of instructions that will be issued, using a short guidance booklet, to the audit manager and ward managers in hospitals which participate in the audit.

7.1 Creation of Audit Management Spreadsheet

In order to assist the hospital in managing the audit, the HfH Programme has developed an Audit Management Spreadsheet, so that the audit manager can track the administration and collection of each of the six questionnaires that make up the audit. This is available on CD. The audit database is essential for ensuring that each questionnaire has the correct ID and can be tracked from the time it leaves the audit manager's office until it is completed and returned to that office. For this reason, each questionnaire and pre-paid HfH envelope, before it leaves the audit manager's office, must have an ID number which matches exactly the details on the audit database. A questionnaire with an incorrect ID, or with no ID, cannot be used in any subsequent analysis. Each completed questionnaire will be returned to the audit manager in a sealed enveloped; the ID number on the envelope will be recorded on the audit database before being posted to the HfH office in Dublin. In order to complete the audit trail, the HfH office in Dublin will send regular up-dates to the audit manager in order to check that the questionnaires received by the HfH office in Dublin match those which have been issued from the audit manager's office in the hospital.

The following procedures are to be followed for administering each questionnaire. All questionnaires are colour coded to avoid confusion.

7.2 Questionnaires 1 & 2: (Patient Profile - Nurse & Doctor Versions)

These two questionnaires will be completed as soon as possible after the patient has died, preferably within one week of the death. The target is to complete both questionnaires on 50 deaths over a four month period. However, because hospitals vary in size, the following procedures have been put in place for larger and smaller hospitals:

- In hospitals where the number of deaths in the previous year exceeded 150, and for whom the target of 50 deaths can be achieved in the four months, the H*f*H Programme will set a quota of deaths to be completed during each of the four months of the audit. This quota will reflect the proportion of hospital deaths in the previous years broken down by: (i) A & E (ii) intensive care and (iii) other wards. The quota in each of these

categories will be completed by taking all consecutive deaths from the beginning of each month until the quota for that month is reached.

- In hospitals where the number of deaths in the previous year was less than 150, and for whom the target of 50 deaths is unlikely to be achieved in the four months, every consecutive death within the audit period will be included.

As soon as a death is notified to the audit manager, the following procedures will be followed:

1. The audit manager will enter as many details as are available on the audit database; any missing data will be collected as soon as possible, preferably within days of the patient's death.
2. The audit manager will identify the ward where the patient died, and identify the name of the ward manager (CNM2) and the patient's consultant.
3. The audit manager will write the correct ID numbers on the cover-page of the Patient Profile Questionnaire (Nurse and Doctor Versions), matching those in the audit database. Each questionnaire will be accompanied by a pre-paid H*f*H envelope.
4. The audit manager will give the Patient Profile (Nurse Version) Questionnaire to the ward manager (CNM2) who, in turn, will designate a nurse to take responsibility for completing this questionnaire. Ideally, this will be the nurse who provided most care to the patient during last week of life. It is possible that a number of other nurses, including specialist palliative care nurses, may have been involved in the care of the patient during the last week, and it may be helpful if the nurse designated to complete the questionnaire were to consult these. However it is recommended that one nurse is designated as having overall responsibility for completing this questionnaire; and this should be done within a week of the death. It will take approximately 60 minutes to complete this questionnaire, and it is recommended that each designated nurse is given protected time and space to complete it. Once completed, the designated nurse – who should not be a student nurse - will put the questionnaire in the pre-paid H*f*H envelope, seal it, and return to the ward manager (CNM2) within a week; the ward manager (CNM2) will then return the sealed envelope to the audit manager. Note that this nurse will not participate in the Survey of Ward Perceptions since the same sub-set of questions are already in the Patient Profile (Nurse Version) Questionnaire.
5. The audit manager will give the Patient Profile (Doctor Version) Questionnaire to the consultant, or may ask the ward manager (CNM2) to do so, whichever is the most convenient for both. The consultant may complete the questionnaire or designate a doctor – but not a trainee doctor - to do so. The consultant / doctor will be asked to put the completed questionnaire in the pre-paid H*f*H envelope, seal it, and return to the ward manager (CNM2) within a week, who will then return it to the audit manager.
6. The audit manager will follow-up with the ward manager (CNM2) and / or consultant if the Patient Profile Questionnaire (Nurse or Doctor) has not been returned within one week. It is possible that delays may arise, particularly if the case is referred to the coroner; in this case, it may be necessary for the nurse / doctor to go the mortuary to access the patient's hospital record. In those cases, a new deadline will be agreed with the nurse / doctor for return of the completed questionnaire to the ward manager (CNM2).

7.3 Questionnaire 3: Survey of Bereaved Relatives / Friends

The bereaved relatives / friends of patients in the audit will be invited to participate in a survey on end-of-life care, and end-of-life experiences in the hospital. For the purpose of this survey, the bereaved relative / friend will be the person named in the patient's hospital

chart as the designated contact person. The survey will involve the following four-step procedure:

1. Three months after the death of each patient in the audit, the Director of Nursing (DoN) or a person nominated by the DoN, will send a letter of invitation to the bereaved relative, offer sympathy on their bereavement, explain that a survey of bereaved relatives is being undertaken, and invite them to participate. Before sending this letter, the audit manager will check that there are no outstanding legal issues pending, such as an inquest into the cause of the patient's death. There is a copy of this letter in Appendix Seven of the Manual.
2. One week after this letter of invitation, the DoN or a person nominated by the DoN, will phone the bereaved relative to offer sympathy, explain the survey, and request consent to send out the questionnaire; where consent is given, the bereaved relative will be told that they will receive the questionnaire within a couple of days. The person making the phone call may suggest to the bereaved relative that s/he can seek the assistance of other family members or friends in filling out the questionnaire, if that would help. Equally, if the bereaved relative wishes to make a complaint, the person phoning from the hospital will inform him / her as to the procedures for doing so. A briefing document has been prepared to assist hospital staff in making these phone calls, and is in Appendix Eight of the Manual.
3. If consent is given by the bereaved relative, this is documented by the audit manager, and the questionnaire will be sent out within a couple of days of the phone call. Before being sent out, the audit manager will ensure that the bereaved relative questionnaire has the correct ID number, matching the ID number on the corresponding patient profile questionnaire, and on the audit database; in this way, all questionnaires can be subsequently linked in the analysis. In addition to the questionnaire, a pre-paid H*f*H envelope will be included so that the completed questionnaire can be returned to the H*f*H office in Dublin. The enclosures will also include a covering letter explaining the purpose of the survey (Appendix Nine of the Manual), and an information leaflet about bereavement (Appendix Ten of the Manual). The covering letter will also inform bereaved relatives that they may phone a help-line in the H*f*H office, if they need to talk to someone in confidence about their bereavement.
4. Two weeks after the questionnaire has been sent out, a final letter is sent by the DoN or a person nominated by the DoN, to all bereaved relatives who have received the questionnaire. This letter will thank those who have already returned their questionnaires, and request those who have not to so as soon as possible. There is a copy of this letter in Appendix Eleven of the Manual.

7.4 Questionnaire 4: Survey of Ward Perceptions

The survey of ward perceptions will be carried out in those wards where a patient's death has been included in the audit. Given that the number of wards to be included in this survey can only be established at the end of the four month audit period, the survey of ward perceptions will be completed in the following month.

This survey will completed by 10 ward staff, comprising nurses and health care assistants. The number of nurses and health care assistants in the sample will reflect their proportion within the ward. The names of the nurses to be included in the sample will be drawn at random from the list of all nurses working on that ward, excluding those employed on an agency basis. Similarly, the names of the health care assistants to complete the ward perceptions questionnaire will be selected at random from the list of all health care assistants working on that ward, excluding those employed on an agency basis.

The procedures for carrying out the survey in each ward are:

1. The audit manager will inform the HfH Programme in Dublin on the number of wards where a patient's death has been included in the audit. From this, the HfH Programme will estimate the total number of questionnaires to be supplied to the hospital (based on number of wards x 10 questionnaires per ward).
2. The audit manager will prepare 10 Ward Perceptions Questionnaires for each ward, by writing on each cover-page, and envelope, the hospital, ward, and questionnaire ID. Each questionnaire will be distributed with a pre-paid HfH envelope which will also have the hospital, ward, and questionnaire ID.
3. The audit manager will deliver 10 Ward Perceptions Questionnaires to each ward manager (CNM2), and will assist the ward manager (CNM2) in randomly selecting names from the list of nurses and the list of health care assistants to fill their respective quotas.
4. The ward manager (CNM2) will give the Ward Perception Questionnaire and a pre-paid HfH envelope to the selected staff. In the course of doing so, the Ward Manager (CNM2) will create a list containing the name of each staff who has received a questionnaire and the corresponding questionnaire ID.
5. The ward manager (CNM2) will allow a week for the questionnaire to be completed and returned in a sealed envelope. Each returned questionnaire will be ticked against the list of staff who received a questionnaire; in this way, the ward manager (CNM2) will be able to track those questionnaires that have not been returned, and a further deadline of one week will be agreed with the staff concerned.
6. The ward manager (CNM2) will send all of the completed questionnaires to the audit manager, who will record the number of returned questionnaires on the database, and then post them to the HfH office in Dublin.

7.5 Questionnaire 5: Survey of Hospital Perceptions

The survey of hospital perceptions will be completed by 100 staff across all categories in the hospital, excluding nurses and health care assistants who are already included in the survey of ward perceptions. The sample will reflect the overall distribution of staff in the hospital, subject to the requirement that at least one staff must be selected from each of the following in order to ensure that numerically small staff categories are also represented: CEO / General manager, nursing management (DoN, Ass DoN, CNM3), consultant doctors, non-consultant doctors, allied health professionals, pastoral carers, bereavement coordinators, end-of-life care coordinators, porters, catering staff, household staff, mortuary staff, security staff, complaints officer / patient advice & liaison officer, ward clerk, reception, administration. The quota for each category of staff will be set by the HfH Programme on the basis of information supplied by the hospital in the Hospital Profile Questionnaire. Ideally, the quota for each category of staff will be filled by randomly selecting names from the list of all staff in each category. Where this is not possible, the questionnaire will be distributed as widely as possible among each staff category until the quota is met.

The procedures for completing this survey are:

1. The HfH Programme will inform the audit manager about the quota of staff in each category who will complete the Hospital Perceptions Questionnaire.
2. The audit manager will prepare 100 Hospital Perceptions Questionnaires by writing on the cover-page the hospital and questionnaire ID. Each questionnaire will be distributed with a pre-paid HfH envelope which will also have the hospital and questionnaire ID.

3. The audit manager will deliver the quota of questionnaires to each head of service, and assist the head of service in randomly selecting the names from their list of staff to fill the quota.
4. The head of service will give the Hospital Perceptions Questionnaire and a pre-paid H*f*H envelope to the selected staff. In the course of doing so, the head of service will create a list containing the name of each staff who has received the questionnaire and the corresponding questionnaire ID.
5. The head of service will allow a week for the questionnaire to be completed and returned in a sealed envelope. Each returned questionnaire will be ticked against the list of staff who received a questionnaire; in this way, the head of service will be able to track those questionnaires that have not been returned, and a further deadline of one week will be agreed with the staff concerned.
6. The head of service will send all of the completed questionnaires to the audit manager, who will record the number of returned questionnaires on the database, and then post them to the H*f*H office in Dublin.

7.6 Questionnaire 6: Hospital Profile

This questionnaire will be completed by a person nominated by the General Manager or the Director of Nursing. The questionnaire will require an intimate knowledge of the hospital and its information systems. This is because it covers a range of questions to assess the hospital's size and capacity such as number of beds, patients, deaths and staff, as well as assessments of the hospital's facilities for end-of-life care, including its procedures and resources. Some of the questions involve matters of judgement, such as questions about the extent to which the hospital meets certain standards for end-of-life care (Sections F, J, K and L). In view of this, the answers should reflect the overall assessment of hospital management. The questionnaire is scheduled to be completed in February 2009, based on 2008 data. When completed, it will be given to the audit manager who will record on the audit database that it has been completed, and then post it to the H*f*H office in Dublin. In turn, the H*f*H office will confirm that it has been received.

8. Data Storage

All questionnaires will be sent to, and stored at, the H*f*H office in Dublin, which is located in the Irish Hospice Foundation, 32 Nassau Street, Dublin 2. These questionnaires will be computerised and access to the computerised audit dataset will be controlled by the Evaluation Coordinator of the H*f*H Programme. Once the questionnaires have been computerised, the hard copies will be shredded. All members of the research team (see Section 12 below) will have access to the computerised dataset, and their computers will be password protected. Given that all of the data is anonymised, nothing in this electronic database could be used to identify any hospital, ward, doctor, nurse, patient or relative. The computerised dataset will be stored until at least 2012 when a follow-up audit is undertaken.

9. Data Analysis

The audit system is designed to provide a comprehensive dataset on end-of-life care where individual-level and hospital-level data are seamlessly integrated. In order to make the most of this data, taking into account the needs of different stakeholders, it will be necessary to produce detailed descriptive statistics for each variable in the system, as well as more advanced statistical analysis of how selected dependent variables – such as end-of-life care and end-of-life experience – are influenced by the range of independent variables within the system. In addition, since data will be collected at baseline and follow-up, it will be necessary to calculate the extent and significance of change between these two waves of

data collection. This implies that a range of statistical approaches will be necessary to analyse and report the results of the baseline and follow-up studies.

9.1 Baseline Study

Beginning with the baseline study, we will undertake two broad types of analyses: the first will involve producing descriptive statistics, and the second will involve statistical analysis. Most audit reports – whether of dying[56], strokes[57] or cancer[58] – are based on descriptive statistics such as frequencies, measures of central tendency (mean, median, mode), and dispersion (standard deviation, inter-quartile range, box plots); in addition to these, our audit report will also contain the results of statistical analysis.

The descriptive statistics will comprise a comprehensive set of tables which report on the frequency, mean and range scores for each variable, broken down by whether the hospital is H*f*H or non- H*f*H, and by whether it is acute or community. In this way, the scores for each individual hospital can be compared with the total for all hospitals, and with the sub-category to which each hospital belongs. To ascertain the practical significance in observed differences in means and standard deviations for each statistic, we will employ measures of effect size (see footnote above). This, in turn, will allow statistical norms for end-of-life care to be established – in addition to the normative H*f*H standards for end-of-life care – and for the hospital's position to be indicated for each individual variable. The output of this work will be presented in an overall Statistical Report and in Individual Hospital Reports, and will also inform the analysis in the Main Report (see Section 8 below). Most audits reports – whether of dying, strokes or cancer – typically confine the analysis to these descriptive statistics. Audit data typically involves the use of relatively simple statistics –As such, it is much less sensitive to sample size compared to the more advanced statistics required for research and evaluation (such as regression and multi-level modelling).

The statistical analysis will also involve an exploration of the relationships between various output and outcome variables – notably the quality and experience of end-of-life care – and the range of individual-level and hospital-level characteristics within the dataset. We will use Multilevel Modelling to undertake this analysis, since it provides the statistical techniques necessary in order to study the data produced by a clustered sample design such as that proposed in this Manual. This also facilitates the inclusion of scales comprising multiple indicators. The results of this analysis will be used in the Main Report to elucidate the multi-level influences on end-of-life care and to identify pathways for improving the quality of care.

The overall rationale for the statistical analysis is that it will make maximum use of the data in order to create the most inclusive and sensitive indicators possible of end-of-life care, while also helping to inform the change-strategy of the H*f*H Programme by identifying the variables which are shown to have the most direct or indirect influence on end-of-life care, at both individual-level and hospital-level.

9.2 Follow-up Study

Turning to the follow-up study, this will involve the same types of analysis as before: descriptive statistics, statistical tests and statistical models. The descriptive statistics will comprise a comprehensive set of tables giving the frequency and mean scores for each variable at both baseline and follow-up, broken down by the category of hospital (H*f*H or non- H*f*H, acute or community). The differences between baseline and follow-up will be expressed in terms of effect sizes and evaluated using standard statistical tests. This will enable us to assess whether programme implementation in the H*f*H hospitals was associated with a statistically significant improvement in the quality or experience of end-of-

56 Marie Curie Palliative Care Institute Liverpool, 2008
57 Irish National Audit of Stroke Care Research Team, 2008
58 Cancer Audit Programme Team St. James's Hospital, 2008

life care. During this phase we will explore a range of different techniques for controlling for the differences between sub-groups of hospitals at baseline and during programme implementation. The output of this work will be presented in the Statistical Report and in Individual Hospital Reports, and will also inform the analysis in the Main Report (see Section 10 below).

The statistical analysis of the follow-up data will, once again, involve an exploration of the relationships between various output and outcome variables and the range of individual-level and hospital-level characteristics within the dataset, this time including data on the implementation of the H*f*H Programme. We will once again use Multilevel Modelling to undertake this analysis. The results of this analysis will be written up in the Main Report and will further inform the change-strategy of the H*f*H Programme by identifying the variables which represent the most effective levers of change in end-of-life care at hospital level.

10. Outputs of National Audit of End-of-Life Care

In keeping with the rationale for the audit system, as described above (see Section 3), a range of outputs will be created to assist in implementing the H*f*H Programme. These outputs will deepen understanding of the factors associated with end-of-life care, and evaluate the extent of change in end-of-life care over the course of the programme. Three core outputs will be produced following the baseline and follow-up studies:

(i) A **Main Report**, including a **Summary Report**. This will provide an overview of results for all hospitals combined, including a descriptive account of the context, quality of care and patient experiences of end-of-life care; a statistical analysis of the factors associated with end-of-life care; and a considered assessment of the implications for policy and practice. In the follow-up study, we will also analyse the factors associated with any changes in end-of-life care since baseline.

(ii) A **Statistical Report**. This will comprise a comprehensive set of tables on the frequency and mean scores for each variable, cross-tabulated by the confidential ID number of each individual hospital, and whether the hospital is H*f*H or non-H*f*H, acute or community. In the follow-up study, these tables will also indicate the extent of change between baseline and follow-up.

(iii) An **Individual Hospital Report**. This will comprise 4-5 pages of text and charts, highlighting the performance of each H*f*H hospital relative to the established standards for end-of-life care, and relative to the average performance of other hospitals. This report represents a precious resource to facilitate the hospital in meeting H*f*H standards for end-of-life care, and will be provided in accordance with agreed confidentiality requirements.

In addition to these core outputs, it is the intention of the programme to disseminate the results of the baseline and follow-up studies as widely as possible, and in a form to suit different audiences. This intention is outlined in the publication and dissemination strategy, which has been adopted by the H*f*H Evaluation Sub-Committee (See Appendix Twelve). The strategy states: "It is important that outputs from the evaluation are widely disseminated and appear in a variety of journals and other publications. Some significant 'summative' papers are expected to appear in high impact factor, peer-reviewed journals. It is also important that evaluation results are published in professional journals, magazines, newsletters, conference symposia, etc., even though these may not be peer-reviewed".

11. Timeframe for Implementing National Audit End-of-Life Care

The timeframe for implementing the audit system is summarised in Table 9. Given that the baseline study is the top priority for 2008/9, we have given greater consideration to timetabling the specific tasks associated with this. Most hospitals will start the audit in February 2009, and that is the start-date indicated in Table 9; however a small number of hospitals are starting the audit in November 2008.

Table 9 Timetable for Implementing National Audit of End-of-Life Care, 2008-2012

Task	Start Date	Finish Date
**Baseline Audit**		
Complete Questionnaire 1: patient profile (nurse version)	February 09*	May 09*
Complete Questionnaire 2: patient profile (doctor version) at same time	February 09*	May 09*
Complete Questionnaire 6: hospital profile, based on 2008 data	February 09	February 09
Complete Questionnaire 3: bereaved relatives	May 09**	September 09**
Complete Questionnaire 4: ward perceptions	March 08	June 08
Complete Questionnaire 5: hospital perceptions	April 08	April 08
Computerisation of data	January 09	September 09
Data analysis and preliminary reports	October 09	December 09
Preparation of Reports	October 09	March 10
Launch of Main Report	March 10	March 10
**Follow-up Audit**		
Repeat the same tasks as in baseline audit	June 2011	December 2012
**Other Studies**		
Case studies of end-of-life journeys	January 2009	December 2012
Qualitative study of H*f*H from an advocacy perspective	January 2009	December 2012
Review of deaths in hospitals using a case study approach	January 2009	December 2012

Notes to Table 9:
*There are three start dates for the audit of patient deaths: 1-11-2008, 1-12-2008, and 1-2-2009. The corresponding completion dates are four months later namely, 1-3-2009, 1-4-2009, and 1-5-2009.
**There are three start dates for the survey of bereaved relatives: 1-2- 2009, 1-3-2009, and 1-5- 2009. The corresponding completion dates are four months later namely, 1-6-2009, 1-7-2009, 1-9-2009.

12. Research Team

The Research Team, working closely with the Evaluation Sub-committee and with staff in the H*f*H Programme, has the experience and expertise to ensure that the audit system is carried out to the highest scientific standards. The Research Team comprises:

- Dr. Kieran McKeown, Evaluation Coordinator
- Mr. Trutz Haase, Data Analyst
- Dr. Jonathan Pratschke, Statistical Consultant

12.1 Kieran McKeown, Evaluation Coordinator

Kieran McKeown, B.Soc.Sc., M.A.(Econ), PhD., is a social and economic research consultant, and is the Principal Investigator on the study. He has carried out over 100 different research projects, many of them large-scale, and involving evaluations of programmes and services. He has worked for almost every Government Department in Ireland, many statutory and voluntary bodies, and the EU Commission. His work has covered the full spectrum of research methodologies including: evaluation of programmes based on comparing pre-intervention and post intervention data, also using data from matched non-programme groups; designing randomised control trials; carrying out surveys based on national and local samples using structured questionnaires; familiarity with a range of statistical techniques; qualitative research based on in-depth interviews and focus groups. He has considerable experience of leading research projects and organising the process of data gathering, analysis, interpretation, and write-up. A more detailed profile and CV is available on request.

As Evaluation Coordinator, Kieran McKeown's role will be to ensure that the objectives of the audit system, as stated in this Manual, are achieved to the highest standards, and in the most cost effective and ethical manner. This will be achieved by working closely with all members of the H*f*H team, and with the participating hospitals, to ensure that each step of the research process is valid and reliable, and contributes directly to the study's objectives. He is responsible for designing the audit and writing the Manual, preparing the questionnaires, recruiting and managing the research team, ensuring that all fieldwork is carried out as planned, overseeing the storage and management of data, contributing to the analysis of data, writing reports, drafting articles for publication, and providing regular updates and progress reports to the H*f*H National Steering Committee and Evaluation Sub-Committee.

12.2 Trutz Haase, Data Analyst

Mr. Trutz Haase, B.A., is the Data Analyst for the study. His speciality is the analysis of complex datasets. In addition to multivariate regression techniques and their extension to non-linearity and interaction effects, his interests embrace structural equation modelling and multi-level modelling, which can be used to predict behavioural outcomes. He has worked for a number of Irish Government Departments, Northern Ireland Statistics & Research Agency, Local Authorities, non-governmental agencies, as well as the OECD. He is best known for his statistical analysis of national databases – notably the census of population - and the development of an Irish Index of Relative Affluence and Deprivation which features in the current Irish Regional and Local Development Plans. A more detailed profile is available on request.

12.3 Jonathan Pratschke, Statistical Consultant

Dr. Jonathan Pratschke B.A., M.A., PhD., is a University Lecturer in Sociology and a Research Consultant on Statistical Modelling. Dr. Pratschke has specialist training and extensive experience in research design, the analysis of institutional and administrative data sources and the construction and estimation of statistical models, including complex structural and hierarchical linear models. His role in the team will be to develop, estimate and interpret the statistical models used to explore the impacts of the H*f*H Programme, and the factors that influence end-of-life care in the study hospitals.

References

Addington-Hall, J., Walker, L. Jones, C., Karlsen, S., and McCarthy, M., 1998. A randomised controlled trial of postal versus interviewer administration of a questionnaire measuring satisfaction with, and use of, services received in the year before death, *J Epidemiology Community Health,* 1998;**52**:802–807.

Andec Communications, 2007. Communications Training: Trainer's Pack, Unpublished. Dublin: Hospice Friendly Hospitals Programme and Andec Communications.

Barry, LC., Kasl, SV., and Prigerson, HG., 2002. Psychiatric Disorders Among Bereaved Persons: The Role of Perceived Circumstances of Death and Preparedness for Death, *American Journal of Geriatric Psychiatry,* 10:4, July-August, pp.447-457.

Bonanno, GA., Wortman, CB., et al, 2002. Resilience to Loss and Chronic Grief: A Prospective Study from Preloss to 18-Months Postloss, Journal of personality and social Psychology, Vol 83, No 5, pp.1150-1164.

Burnett, P., Middleton, W., Raphael, B., and Martinek, N., 1997. Measuring Core Bereavement Phenomena, *Psychological Medicine,* 27, 49-57.

Cancer Audit Programme Team St. James's Hospital, 2008. Six-Year Cancer Audit Report, St. James's Hospital, 2001-2006, Dublin: St. James's Hospital.

Central Statistics Office, 2006. Report on Vital Statistics 2004, December. Dublin: Stationery Office. Available at http://www.cso.ie/releasespublications/documents/vitalstats/2004/annualreport_2004.pdf

Centre for Health Promotion Studies, 2003. The National Health and Lifestyle Surveys: Survey of Lifestyle, Attitudes and Nutrition (SÁN) & The Irish Health Behaviour in School-Aged Children Survey (HBSC), Galway: Centre for Health Promotion Studies.

Cohen, J., 1988. Statistical Power Analysis for the Behavioural Sciences, Second Edition, New Jersey: LEA.

Cohen, J., 1992. "A Power Primer", Psychological Bulletin, Vol 112, No 1, pp.155-159.

Cohen Fineberg, I., and Hughes, N., 2007. Literature Review of "Integrated Care" for the Irish Hospice Foundation's Hospice Friendly Hospitals Programme, April, Unpublished, Dublin: Hospice Friendly Hospitals Programme.

Cohen Fineberg, I., and Miller, PK., 2008. Literature Review of "Communication" for the Irish Hospice Foundation's Hospice Friendly Hospitals Programme, February, Unpublished, Dublin: Hospice Friendly Hospitals Programme.

Commission on Patient Safety and Quality Assurance, 2008. Building a Culture of Patient Safety: Report of the Commission on Patient Safety and Quality Assurance, August, Dublin: Stationery Office.

Connor, S. R., Teno, J., Spence, C., & Smith, N., 2005. Family evaluation of hospice care: results from voluntary submission of data via website. *J Pain Symptom Manage,* 30(1), 9-17.

Costantini, M., Beccaro, M., & Merlo, F. 2005. The last three months of life of Italian cancer patients. Methods, sample characteristics and response rate of the Italian Survey of the Dying of Cancer (ISDOC). *Palliat Med,* 19(8), 628-638.

Curtis, J. R., Patrick, D. L., Engelberg, R. A., Norris, K., Asp, C., & Byock, I., 2002. A measure of the quality of dying and death. Initial validation using after-death interviews with family members. *J Pain Symptom Manage,* 24(1), 17-31.

Data Protection Commissioner, 2007. Data Protection Guidelines on Research in the Health Sector, Portarlington: Office of the Data Protection Commissioner. Available at: http://www.dataprotection.ie/documents/guidance/Health_research.pdf

Department of Health and Children, 2008. Discussion Paper on Proposed Health Informaiton Bill, June. Available at: http://www.dohc.ie/issues/hib/

Dublin Hospitals Group Risk Management Forum, 2006. Guidelines in relation to Obtaining Patient Consent, Dublin: Dublin Hospitals Group Risk Management Forum.

European Commission, 2008. EU Research Programme FP7, Work Programme 2009, Theme 1: Health, March, Unpublished.

Fahey, T., Hayes, BC., and Sinnott, R., 2005. Conflict and Consensus: A Study of Values and Attitudes in the Republic of Ireland and Northern Ireland, Dublin: Institute of Public Administration.

Fahey, T., 2007. 'How do we feel? Economic Boom and Happiness', in Fahey, T., Russell, H., and Whelan CT., (Editors), Best of Times? The Social Impact of the Celtic Tiger, Dublin: Institute of Public Administration, pp.11-26.

Ferrand, E., Jabre, P., Vincent-Genod, C., et al, 2008. Circumstances of Death in Hospitalized Patients and Nurses' Perceptions, *Arch Intern Med,* 168(8), 28 April, 867-875.

Health Information and Quality Authority, 2008. National Quality Standards for Residential Care Settings for Older People in Ireland, March, Dublin: Health Information and Quality Authority. Available at: http://www.hiqa.ie/news_releases/080311_hiqa_national_quality_standards_residential_care.asp

Health Service Executive, 2006. Transformation Programme, 2007-2010, December, Dublin: Health Service Executive.

Health Service Executive and the Irish Hospice Foundation, 2008. Palliative Care for All: Integrating Palliative Care into Disease Management Frameworks, Report for Consultation, June, Dublin: Health Service Executive and the Irish Hospice Foundation.

Hodde, N. M., Engelberg, R. A., Treece, P. D., Steinberg, K. P., & Curtis, J. R., 2004. Factors associated with nurse assessment of the quality of dying and death in the intensive care unit. *Crit Care Med,* 32(8), 1648-1653.

Hospice Friendly Hospitals Programme, 2008. Design and Dignity Guidelines for Physical Environments of Hospitals Supporting End-of-Life Care, June, Dublin: Hospice Friendly Hospitals Programme. Available at http://www.hospicefriendlyhospitals.net

Hugodot, A., and Normand, C., 2007. Design, Dignity and Privacy in Care at the End of Life in Hospital: A Review of the Literature, TCD Centre for Health Policy and Management,

Unpublished. November, Dublin: Hospice Friendly Hospitals Programme. Available at http://www.hospicefriendlyhospitals.net

Hugodot, A., and Normand, C., 2008. Cost-effectiveness of Palliative Care in Hospital: A Review of the Literature, TCD Centre for Health Policy and Management, February, Unpublished. Dublin: Hospice Friendly Hospitals Programme.

Hugodot, A., 2008. Review of Deaths System(s) in Hospital: A Review of the Literature, April, Unpublished. Dublin: Hospice Friendly Hospitals Programme.

Ingleton, C., Morgan, J., Hughes, P., Noble, B., Evans, A., & Clark, D. 2004. Carer satisfaction with end-of-life care in Powys, Wales: a cross-sectional survey. *Health Soc Care Community,* 12(1), 43-52.

Irish National Audit of Stroke Care Research Team, 2008. National Audit of Stroke Care, April, Dublin: Irish Heart Foundation.

Madden, D., 2006. Report of Dr. Deirdre Madden on Post Mortem Practice and Procedures, January, Dublin: Stationery Office. Available at: http://www.dohc.ie/publications/madden.html

Marie Curie Palliative Care Institute Liverpool, 2008. National Care of the Dying Audit – Hospitals (NCDAH) – Generic Report 2006/2007, Liverpool: Marie Curie Palliative Care Institute Liverpool. Available at: www.mcpcil.org.uk

McCarthy, S. O'Boyle C., 2007. Literature Review for Survey of Bereaved Relatives. Unpublished. Dublin: Hospice Friendly Hospitals Programme.

McDowell, I., 2006. Measuring Health: A Guide to Rating Scales and Questionnaires, New York: Oxford University Press, pp.350-358.

Mularski, R. A., Heine, C. E., Osborne, M. L., Ganzini, L., & Curtis, J. R., 2005. Quality of dying in the ICU: ratings by family members. *Chest, 128*(1), 280-287.

Murray, E., Sweeney, C., Smyth, C., and Conolly, E., 2006. A Baseline Study on the Provision of Hospice / Specialist Palliative Care Services in Ireland, March, Dublin: The Irish Hospice Foundation.

Murray, E., 2008. Has Ireland's National Policy Benefited Palliative Care? European Journal of Palliative Care, 15(2), pp.72-76.

National Advisory Committee on Palliative Care, 2001. Report, Dublin: Department of Health & Children.

National Institute of Clinical Excellence, 2002. Principles for Best Practice in Clinical Audit 2002, Oxford: Radcliff Medical Press.

O'Shea, E., Murphy, K., Larkin, P., Payne, S., et al, 2008. End-of-Life Care for Older People in Acute and Long-Stay Care Settings in Ireland, April, Dublin: National Council on Ageing and Older People and The Irish Hospice Foundation.

PA Consulting Group, 2007. Acute Hospital Bed Capacity Review: A Preferred Health System in Ireland to 2020, Detailed Report, September, Dublin: Health Service Executive.

Prospectus, 2008. Business Case for the Development of an All-Ireland Institute for Hospice and Palliative Care. Final Version – March. Unpublished. Dublin: Irish Hospice Foundation.

Parsley, K., and Corrigan, P., 1999. Quality Improvement in Healthcare - Putting Evidence into Practice Second Edition: Cheltenham, UK: Nelson Thornes.

Rasbash, J., Browne, W., Goldstein, H., *et al*. 2001. A User's Guide to MLwiN Centre for Multilevel Modelling. London: Institute of Education, University of London.

Rhodes, RL., Mitchell, SI., Miller, SC., Connor, SR., and Teno, JM., 2008. Bereaved Family Members' Evaluation of Hospice Care: What Factors Influence Overall Satisfaction with Services?, *J Pain Symptom Manage,* 35(4), 365-371.

Sheikh, AA., 2008. The Data Protection Acts 1988 and 2003: Some Implications for Public Health and Medical Research, HRB Discussion Document, July, Dublin: Health Research Board. Available at: http://www.hrb.ie/storage/researchfunding/fundingpolicies/DataProtectionOpinionV6.pdf

Snijders, T. A. B. & Bosker, R. J. 1993. Standard errors and sample sizes in two-level research. *Journal of Educational Statistics*, 18(3), 237-260.

Snijders, T. A. B. 2005 Power and Sample Size in Multilevel Linear Models. In B.S. Everitt and D.C. Howell (eds.), *Encyclopedia of Statistics in Behavioral Science*. Volume 3, 1570–1573. Chicester: Wiley.

Teno, J. M., Clarridge, B., Casey, V., Edgman-Levitan, S., & Fowler, J., 2001. Validation of Toolkit After-Death Bereaved Family Member Interview. *J Pain Symptom Manage,* 22(3), 752-758.

Teno, JM., Janet, E., Shu, BS, Casarett, D., Spence, C., Rhodes, R., and Connor, S., 2007. Timing of Referral to Hospice and Quality of Care: Length of Stay and Bereaved Family Members' Perceptions of the Timing of Hospice Referral, *Journal of Pain and Symptom Management*, Vol. 34, No. 2, August, pp.120-125.

Tribal, 2007. Design and Dignity Baseline Review, November, Dublin: Hospice Friendly Hospitals Programme. Available at: http://www.hospicefriendlyhospitals.net

Van Belle, G., 2002. Statistical Rules of Thumb, New York and London: John Wiley and Sons.

Watson, M. Ó Brannagáin, D., and Keegan, O., 2007. 'Ireland attempts to change culture for care of the dying', *European Journal of Palliative Care, 14(6).*

Watson, D., Clark, L., and Tellegen, A., 1988. "Development and Validation of Brief Measures of Positive and Negative Affect: The PANAS Scales", Journal of Personality and Social Psychology, 54(6), 1063-1070.

Weafer and Associates Research and Consultancy Ltd, 2007. Untitled and Unpublished. October, Dublin: Hospice Friendly Hospitals Programme.

Working Group on Post Mortem Practice, 2006. Report of Working Group on Post Mortem Practice November, Dublin: Department of Health and Children. Available at: http://www.dohc.ie/publications/post_mortem_2006.html

Yeates, N., 2006. 'Changing Places: Ireland in the International Division of Reproductive Labour, Translocations', Autumn, Volume 1, Issue 1, pp.5-21.

Appendix 1

Questionnaire 1

Patient Profile (Nurse Version)

(Green)

Questionnaire 1:
Patient Profile - Nurse Version

Guide to Instruments

Question	Concept / Domain	Instrument
	Hospital ID	This number will be allocated to each hospital by the H*f*H Programme, and will remain confidential to each hospital.
	Ward ID	This number (from one onwards) will be allocated by the designated audit manager in each hospital, with a separate number allocated to each ward in the audit.
	Patient ID	This number (from one onwards) will be allocated by the designated audit manager in each hospital, with a separate number allocated to each patient in the audit.
PART ONE: **PATIENT PROFILE**		
**SECTION A: BACKGROUND INFORMATION ON PATIENT**		
A1	Gender	Standard question
A2	Patient's age	Standard question
A3	Date of last admission to hospital	HIPE question
A4.1-2	Time and data of patient's death	HIPE question
A5	Route of admission	Bespoke question
A6	Elective or emergency admission	HIPE question
A7	Where living prior to hospital	Bespoke question
A8	Living alone	Bespoke question
A9.1-2	Type of ward or unit where patient died	Standard question
A10	Time in A&E or intensive care	Standard question
A11.1-2	Primary and secondary illness	Standard question
A12	Was admission following serious trauma or accident	Bespoke question
A13	Was death expected or sudden	Bespoke question
A14	Did patient have dementia	Bespoke question
A15.1-2	Was post-mortem carried out	Bespoke question
A16.1-2	Medical Card and private health insurance	Standard question; socio-economic indicator
A17	Marital status	Question from CSO, 2006 Census of Population
A18	Nationality	Question from CSO, 2006 Census of Population
A19	Ethnic or cultural background	Question from CSO, 2006 Census of Population
A20	Religion	Question from CSO, 2006 Census of Population
**SECTION B: PLACE OF DEATH IN HOSPITAL**		
B1-2	Beds in the ward where the patient died	Bespoke questions
B3	Number of times moved within hospital	Bespoke questions
B4.1-2	Type of room where patient died / spent last week	Bespoke questions
B5.1	Hospital immune infection or immuno-suppression	Bespoke questions
B5.2	Time spent in a single room	Bespoke questions
B6.1-3	Size, occupancy and gender of multi-occupancy room	Bespoke questions

B7.1-15	Quality of physical space where patient spent most of the time during last week	Bespoke questions. Same question used in Survey of Bereaved Relatives, B5.1-16 and Ward Perceptions Questionnaire (C7.1-16).
**SECTION C: AWARENESS THAT PATIENT WAS DYING**		
C1	Had medical team diagnosed dying	Bespoke questions. Same questions used in Patient Profile Questionnaire (Doctor Version A1-5).
C2	Is this documented	
C3	Duration of medical team's awareness of dying	
C4	Awareness of nursing staff that patient was dying	
C5.1-4	Multidisciplinary team-working	
C6	Could patient have died at home	Bespoke question. Same question used in Patient Profile Questionnaire (Doctor Version A6), and Survey of Bereaved Relatives (C26).
**SECTION D: COMMUNICATION WITH PATIENT**		
D1.1-8	Was dying discussed with patient	Bespoke questions
D2.1-10	If no discussion on dying, why not	Bespoke questions
D3	Who initiated and attended the discussion	Bespoke questions
D4	How long before death was it discussed	Bespoke questions
D5.1-7	How patient experienced discussion	Bespoke questions
D6.1-12	Patient concerns about dying and death	Bespoke questions
D7.1-12	Were patient's concerns and wishes addressed	Bespoke questions
D8	Were end-of-life preferences documented	Bespoke questions
**SECTION E: COMMUNICATION WITH RELATIVES**		
E1.1-8	Was dying discussed with relatives	Bespoke questions
E2.1-2	Was patient aware of discussion, and gave consent for it	Bespoke questions
E3.1-8	If no discussion, why not	Bespoke questions
E4.1-6	Who initiated and attended the discussion	Bespoke questions
E5	How long before death was it discussed	Bespoke questions
E6.1-7	How relative experienced discussion	Bespoke questions
E7.1-12	Concerns and wishes expressed by relative	Bespoke questions
E8.1-12	Were patient's concerns and wishes addressed	Bespoke questions
E9.1-6	Services offered to relatives	Bespoke questions
**SECTION F: DECISIONS ABOUT TREATMENT**		
F1.1-11	Decisions made about treatment	Bespoke questions; question on resuscitation (E1.10) was also used in survey of death in French hospitals[59]. Same question used in Patient Profile Questionnaire (Doctor Version B1.1-10)
F2.1-6	Experience and management of specific symptoms	Bespoke question. Same question used in Patient Profile Questionnaire (Doctor Version B2.1-6).
**SECTION G: SPECIALIST PALLIATIVE CARE**		
G1	Is there specialist palliative care service in hospital	Bespoke question. Same question used in Patient Profile Questionnaire (Doctor Version C1-4).
G2	Did patient receive specialist palliative care	
G3	Duration of involvement of palliative care	
G4	Benefits of earlier palliative care	
**SECTION H: QUALITY OF LIFE IN LAST WEEK OF LIFE**		
H1-25	Assessment of patient's experiences during last week of life	Quality of Dying and Death Instrument (QODD)[60]: Significant Other After Death Interview / Seven Day Version. All but eight items included. Same question is used in Survey of Bereaved Relatives, C1-C25.

59 Ferrand, Jabre, Vincent-Genod, et al, 2008.
60 Developed by, and available from, the University of Washington End of Life Care Research Program at: http://depts.washington.edu/eolcare/instruments/index.html. The Quality of Dying and Death Instrument (QODD) was developed by Donald Patrick, Ruth Engleberg and Randall Curtis (Patrick, Engleberg and Curtis 2001) and has been validated in three studies (Curtis, Patrick, Engleberg, Norris, Asp, and Byock, 2002; Hodde, Engelberg, Treece, Steinberg, and Curtis, 2004; Mularski, Heine, Osborne, Ganzini, and Curtis, 2005).

**SECTION J: QUALITY OF CARE**		
J1-5	Overall quality of care	Family Evaluation of Hospice Care (FEHC)[61]: Complete Survey, Compressed Version. Six items selected. Same question used in Patient Profile Questionnaire (Doctor Version D1-5) and in Survey of Bereaved Relatives, D1-C5.
J6.1-3	Who was at bedside at time of death	Based on question in survey of death in French hospitals[62].
J7	Was anyone present from pastoral care team	Bespoke questions
J8.1-2	Acceptability of circumstances in which patient died in hospital	Based on question used in survey of death in French hospitals[63]. Response format changed from 'yes/no' to a 10-point scale. Same question – focused on individual patients - used in Patient Profile Questionnaire (Nurse Version J8.1-2) , Patient Profile Questionnaire (Doctor Version D6.1-2) and Survey of Bereaved Relatives (D6-7). Same question –focused on ward - used in Patient Profile Questionnaire (Nurse Version E17.1-2) and Survey of Ward Perceptions (E17.1-2). Same question –focused on hospital - used in Survey of Hospital Perceptions (D1.1-2).
**SECTION K: TIME AFTER DEATH**		
K1.1-6	Were any rituals held after death	Bespoke questions
K2	Were relatives given time to be with deceased	Bespoke questions
K3	Did staff have enough time to be with relatives	Bespoke questions
K4.1-3	Who accompanied the body to the mortuary?	Bespoke questions
K5.1-9	Information or advice offered to relatives	Bespoke questions
K6	Was GP informed?	Bespoke question
K7.1-2	Time taken to certify and issue death certificate	Bespoke questions
**SECTION L: PERSONAL BELONGINGS OF PATIENT**		
L1	Who assembled the personal belongings?	Bespoke question
L2	Type of bag used for personal belongings	Bespoke question
**SECTION M: REVIEW OF PATIENT'S DYING AND DEATH**		
M1-2	Was there any discussion of the patient's death at ward level.	Bespoke question.
M1-2	Was there any discussion of the patient's death at ward level.	Bespoke question.
M3	Did any staff experience particular upset following the patient's death	Bespoke question.
M4	Were staff given the opportunity to discuss the upset	Bespoke question.
M5	Where did the discussion take place	Bespoke question.
M6.1	What went well?	Open-ended question. Same question is used in Patient Profile Questionnaire (Doctor Version E5.1), and in Survey of Bereaved Relatives, F1.
M6.2	What did not go so well?	Open-ended question. Same question is used in Patient Profile Questionnaire (Doctor Version E5.2), and in Survey of Bereaved Relatives, F2.
M6.3	What could have been better?	Open-ended question. Same question is used in Patient Profile Questionnaire (Doctor Version E5.3), and in Survey of Bereaved Relatives, F3.

PART TWO: **NURSE PROFILE**

61 Developed by, and available from, the National Hospice and Palliative Care Organisation (NHPCO), based on Virginia in the US at: http://www.nhpco.org/i4a/pages/Index.cfm?pageid=4397. The Family Evaluation of Hospice Care (FEHC) was developed by Joan Teno and Stephen Connor at Brown University in the US (Connor, Teno, Spence and Smith, 2005) based on a previously validated scale, Toolkit After-Death Bereaved Family Member Interview (Teno, Clarridge, Casey, Edgman-Levitan and Fowler, 2001).
62 Ferrand, Jabre, Vincent-Genod, et al, 2008.
63 Ferrand, Jabre, Vincent-Genod, et al, 2008.

**SECTION A: BACKGROUND INFORMATION ON NURSE**		
A1	Position within the ward	Bespoke question. Same question as used in Patient Profile Questionnaire (Nurse Version, Section A), Patient Profile Questionnaire (Doctor Version, Section F), Ward Perceptions Questionnaire (Section A), and Hospital Perceptions Questionnaire (Section A).
A2-3	Length of time working in hospital and ward	
A4	Country where brought up	Categories taken from Yeates[64] which summarises the country of origin of nurses in Ireland. Same question as used in Patient Profile Questionnaire (Nurse Version, Section A), Patient Profile Questionnaire (Doctor Version, Section F), Ward Perceptions Questionnaire (Section A), and Hospital Perceptions Questionnaire (Section A).
A5	Gender of respondent	Bespoke question. Same question as used in Patient Profile Questionnaire (Nurse Version, Section A), Patient Profile Questionnaire (Doctor Version, Section F), Ward Perceptions Questionnaire (Section A), and Hospital Perceptions Questionnaire (Section A).
A6	Age of respondent	
A7	Is English your first language?	
**SECTION B: GENERAL ATTITUDES TO DYING AND DEATH**		
B1	Feeling comfortable about discussing death or dying	Based on questions used in 2004 nationwide survey of public attitudes and experiences regarding death and dying[65]. Same questions used in Survey of Ward Perceptions (B1-4), and in Survey of Hospital Perceptions (B1-4).
B2	Feeling comfortable about discussing bereavement	
B3	Preferred place of care if dying	
B4	Impression of care for people who are dying or terminally ill in Irish hospitals	
**SECTION C: CHARACTERISTICS OF WARD**		
C1	Bed occupancy	Bespoke questions. Same questions used in Survey of Ward Perceptions (C1-6).
C2	Patient turnover	
C3	Patient dependency levels	
C4-5	Level and turnover of staff	
C6	Frequency of death on ward	
**SECTION C: WORKING ENVIRONMENT**		
D1-6	Aspects of working environment	Bespoke questions. Same questions used in Survey of Ward Perceptions (D1-6).
D7	Satisfaction with current work situation	Bespoke question. Same question used in Survey of Ward Perceptions (D7).
**SECTION E: END-OF-LIFE CARE ON THIS WARD**		
E1-16	Rating of ward on different aspects of end-of-life care	Bespoke question but informed by standards for end-of-life care[66], including draft H*f*H standards; studies on end-of-life care[67], and the general public's preferences for end-of-life care[68]. Same question used in Survey of Ward Perceptions (E1-16).
E17.1-2	Acceptability of circumstances in which patients generally die in this ward	Based on question used in survey of death in French hospitals[69]. Response format changed from 'yes/no' to a 10-point scale. Same question – focused on individual patients - used in Patient Profile Questionnaire (Nurse Version J8.1-2) , Patient Profile Questionnaire (Doctor Version D6.1-2) and Survey of Bereaved Relatives (D6-7). Same question –focused on ward - used in Patient Profile Questionnaire (Nurse Version E17.1-2) and Survey of Ward Perceptions (E17.1-2). Same question – focused on hospital - used in Survey of Hospital Perceptions (D1.1-2).
E18	One suggestion that would significantly improve end-of-life care in this ward	Open-ended question. Same question used in Survey of Ward Perceptions (E18). Same question – but

64 Yeates, 2006:15
65 Weafer and Associates Research, 2004:8-9.
66 Health Information and Quality Authority, 2008: 23-24.
67 O'Shea, et al, 2008.
68 Weafer and Associates Research, 2004:23-24.
69 Ferrand, Jabre, Vincent-Genod, et al, 2008.

		focused on hospital rather than ward - used in Survey of Hospital Perceptions (D2).
**SECTION F: PROFESSIONAL & PERSONAL PREPARATION FOR END-OF-LIFE CARE**		
F1-4	Indicators of professional and personal preparedness for end-of-life care, including receipt of training	Bespoke question. Same question used in Survey of Ward Perceptions (F1-4) and Survey of Hospital Perceptions (E1-4).
**SECTION G: EXPERIENCES FOLLOWING THE DEATH OF A PATIENT**		
G1	Any experience of particular upset in the past year	Bespoke question. Same question used in Survey of Ward Perceptions (G1), and in Survey of Hospital Perceptions (F1)
G2	Did you need to talk to someone about this experience	Same question used in Survey of Ward Perceptions (G2), and in Survey of Hospital Perceptions (F2)
G3	Did you talk to someone about this experience	Same question used in Survey of Ward Perceptions (G3), and in Survey of Hospital Perceptions (F3)
G4.1-6	Availability of supports if staff experience particular upset	Bespoke question. Same question used in Survey of Ward Perceptions (G3), and in Survey of Hospital Perceptions (F.1-6)
**SECTION H: EDUCATION, TRAINING AND OTHER SUPPORTS FOR END-OF-LIFE CARE**		
H1-11	Adequacy of education, training and other supports for staff to deal with end-of-life care	Bespoke question but informed by standards for end-of-life care[70], including draft H*f*H standards; studies on end-of-life care[71], and studies of the practice environment for nurses[72]. Same question used in Survey of Hospital Perceptions (G1-11), and in Survey of Ward Perceptions (H1-11).
**SECTION J: HOSPITAL PRIORITIES**		
J1-13	Ranking of various hospital priorities	Bespoke question. Same question used in Survey of Ward Perceptions (J1-13), and in Survey of Hospital Perceptions (H1-13)
J14	Classification of hospital's religious ethos	Bespoke question. Same question used in Survey of Ward Perceptions (J14), and in Survey of Ward Perceptions (H14)

70 Health Information and Quality Authority, 2008: 23-24.
71 O'Shea, et al, 2008.
72 Yu-Fang Li, et al., 2007.

Questionnaire 1
Patient profile - Nurse version

Thank you for taking the time to complete this questionnaire. The questionnaire is part of a hospital audit of its end-of-life care. Its main focus is on the services offered to patients in the final week of their life, but it also covers the experiences of relatives and staff. This audit has been ethically approved, and is fully endorsed by the hospital's management team. The audit was developed by the Hospice Friendly Hospitals (H*f*H) Programme, an initiative of the Irish Hospice Foundation. It is part of a national audit of end-of-life care in acute and community hospitals.

After the death of a particular patient, the ward manager (CNM2) will choose or designate a registered nurse who will have responsibility for completing this questionnaire. Ideally, this nurse will have given much of the care during the patient's last week of life. A number of other nurses, including specialist nurses, may also have been involved in the patient's care during his or her last week. The designated nurse may talk with this group if they need to, but the designated nurse has overall responsibility for completing the questionnaire. Once completed, the designated nurse will put the questionnaire in the stamped-addressed envelope, seal it, and return it to the ward manager.

The questionnaire asks for information from the nurse's perspective about the patient and the hospital services the patient got during their last week of life, including care after death (Part one: Patient profile). The questionnaire also collects information about perceptions of the nurse who is completing the questionnaire on a range of end-of-life issues (Part two: Nurse's perceptions).

The H*f*H Programme will analyse the information in this audit and they will write a confidential report for the hospital. They will not use any names or other signifiers in any published reports which could link this information to any particular hospital, to any staff, to any patients, or to their relatives. The value of the audit depends on the quality of the information you give in this questionnaire. For this reason, it is important that you fill out the questionnaire carefully, with accurate and honest answers to each question.

Your answers will remain confidential. You should not write your name on the questionnaire. You should complete the questionnaire as soon as possible after the patient has died, preferably within one week of their death.

Before you begin answering the questionnaire, read these instructions:

- ✓ Check the bottom of this page to make sure it has this information: Hospital ID, Ward ID, Patient ID (for the audit, the patient's ID is different from patient's hospital number). If not, contact the Ward Manager (CNM2) immediately. **Do not start unless the questionnaire has this information.**
- ✓ You may need to have the patient's hospital chart to complete some of the questions.
- ✓ If you are unsure about some part of the patient's care, talk to the Ward Manager (CNM2) or other nursing staff on the ward.
- ✓ Try not to answer 'Don't know', unless you really need to.
- ✓ Make sure that you tick the boxes clearly, and write clearly in the text boxes (especially M6.1 to M6.3).
- ✓ Check that you have answered every question before putting the questionnaire in the envelope provided.
- ✓ Put the completed questionnaire in the stamped-addressed envelope provided, seal it, and return to the Ward Manager (CNM2). They will pass it on to the designated Hospital Audit Manager who will post it to the H*f*H office in Dublin.

Thank you for contributing to your hospital's audit of end-of-life care by completing this questionnaire.

Hospital ID	Ward ID	Patient ID

Part one: Patient profile

Section A: Background information on patient

A1	**Was the patient male or female?**	☐1 Male	☐2 Female

A2	**What age was the patient when he or she died?**	(to the nearest year)

A3	**How long was the patient in hospital before dying? If less than a day, write the number of hours.**	Years	Months	Days	Hours

A4	**At what time did the patient die?**	☐1	1.00-2.00	☐4	7.00-8.00	☐7	13.00-14.00	☐10	19.00-20.00
		☐2	3.00-4.00	☐5	9.00-10.00	☐8	15.00-16.00	☐11	21.00-22.00
		☐3	5.00-6.00	☐6	11.00-12.00	☐9	17.00-18.00	☐12	23.00-24.00

If the patient died in a community hospital, go to A8.

A5	**If the patient died in an acute hospital, how was the patient admitted to hospital?**	Outpatient department ☐1	Accident and Emergency department (A & E) ☐2	Day services ☐3	Medical admission unit ☐4

A6	**If the patient died in an acute hospital, was the admission elective or emergency?**	☐1 Elective	☐2 Emergency	☐3 Other

A7	**Before entering the hospital, where was the patient living?**	Home	Nursing home	Acute hospital	Non-acute hospital	Hospice	Psychiatric unit	Other
		☐1	☐2	☐3	☐4	☐5	☐6	☐7

A8	**If the patient lived at home before entering the hospital, did he or she live alone or with others?**	☐1 Alone	☐2 With others	☐9 Don't know

A9.1	**In what type of ward or unit did the patient die?**	☐1 A & E	☐2 Intensive care*	☐9 Other ward

*Intensive care includes ICU, ITU, CCU, HDU, NITU, operating theatre, etc.

A9.2	**If the patient died in a ward, not including A&E and intensive care*, what type of ward was it?**	Surgical ☐1	Medical ☐2	Oncology ☐3	Geriatric ☐4	Other ☐5

*Intensive care includes ICU, ITU, CCU, HDU, NITU, operating theatre, etc.

A10	**If the patient died in A&E or intensive care, how many days did the patient spend there?**	days

*Intensive care includes ICU, ITU, CCU, HDU, NITU, operating theatre, etc.

Which of these illnesses describes the patient's primary and secondary disease or illness at their last admission to hospital?		Cancer – all types	Heart and circulatory diseases	Lung and breathing diseases	Kidney diseases	Liver diseases	Strokes	Dementia and Alzheimer's	AIDS and infectious diseases	Frailty and decline due to old age	Other
A11.1	**Primary illness** (Tick one only)	☐1	☐2	☐3	☐4	☐5	☐6	☐7	☐8	☐9	☐10
A11.2	**Secondary illness** (Tick one only)	☐1	☐2	☐3	☐4	☐5	☐6	☐7	☐8	☐9	☐10

A12	If the patient died in an acute hospital, was he or she admitted to this hospital after a serious trauma or accident?	☐1 Yes	☐0 No	☐9 Don't know

A13	Was the patient's death expected or sudden?	☐1 Expected	☐2 Sudden	☐9 Don't know

A14	Did the patient have dementia?	☐1 Yes	☐0 No	☐9 Don't know

Was a post-mortem requested after the patient's death, and was it carried out?							
		Post-mortem requested?			Post-mortem carried out?		
A15.1	**Requested by hospital**	☐1 Yes	☐0 No	☐9 Don't know	☐1 Yes	☐0 No	☐9 Don't know
A15.2	**Requested by coroner**	☐1 Yes	☐0 No	☐9 Don't know	☐1 Yes	☐0 No	☐9 Don't know

A16.1	Did the patient have a Medical Card?	☐1 Yes	☐0 No	☐9 Don't know

A16.2	Did the patient have private health insurance?	☐1 Yes	☐0 No	☐9 Don't know

A17	**Was the patient single, married, etc.?**	Single	Married	Living with a partner	Separated	Divorced	Widow or widower	Other	Don't know
		☐1	☐2	☐3	☐4	☐5	☐6	☐7	☐9

A18	**What was the patient's nationality?**	☐1 Irish	☐2 Other EU State	☐3 Other country	☐4 Don't know

A19	**What was the patient's ethnic or cultural background?**	Irish	Other white	African	Other black	Chinese	Other Asian	Other	Don't know
		☐1	☐2	☐3	☐4	☐5	☐6	☐7	☐9

A20	**What was the patient's religion?**	Roman Catholic	Church of Ireland	Other Christian	Muslim	Other (write details)	No religion	Don't know
		☐1	☐2	☐3	☐4	☐5	☐6	☐9

Section B: Patient's place of death in hospital

B1	**How many beds are in the ward there the patient died?**	In-patient beds	Day beds	Total beds

B2	**In this ward, how many in patient beds are in single and multi-occupancy rooms?**	In-patient beds in single rooms	In-patient beds in multi-occupancy rooms	Total in-patient beds

B3	**How many times was the patient moved during their stay in hospital before dying?** Add together (1) the number of times the patient was moved to a different bed and (2) the number of times the patient was moved to a different room?	number of times

B4.1	**Where did the patient die?**	☐1 Single room	☐2 Multi-occupancy room
B4.2	**Where did the patient spend most of the time during their last week of life?**	☐1 Single room	☐2 Multi-occupancy room

B5.1a	**If the patient died in a single room, did he or she have a hospital acquired infection?**	☐1 Yes	☐0 No	☐9 Don't know
B5.1b	**If the patient died in a single room, did he or she have immuno-suppression therapy?**	☐1 Yes	☐0 No	☐9 Don't know

B5.2	**If the patient died in a single room, how much time did he or she spend there during their last week of life?**	days	hours

B6.1	**If the patient died in a multi-occupancy room, how many beds were in that room?**	number of beds
B6.2	**If the patient died in a multi-occupancy room, how many beds were occupied when the patient died?**	number of beds

B6.3	**If the patient died in a multi-occupancy room, was it male only, female only, or mixed?**	☐1 Male only	☐2 Female only	☐3 Mixed

Based on your answer to B4.2 – tell what you think objectively about the type of room the patient spent most of the time during their last week of life.		Very poor 1	2	3	4	5	6	7	8	9	Excellent 10
B7.1	Patients can have private conversations with hospital staff	☐	☐	☐	☐	☐	☐	☐	☐	☐	☐
B7.2	Staff can have private conversations with patients and family members	☐	☐	☐	☐	☐	☐	☐	☐	☐	☐
B7.3	Family members can stay as long as they want	☐	☐	☐	☐	☐	☐	☐	☐	☐	☐
B7.4	Patients have dignity when getting personal care	☐	☐	☐	☐	☐	☐	☐	☐	☐	☐
B7.5	Patients can have easy access to toilet, and shower or bath	☐	☐	☐	☐	☐	☐	☐	☐	☐	☐
B7.6	Patients can choose company, or to be alone	☐	☐	☐	☐	☐	☐	☐	☐	☐	☐
B7.7	Patients can see nature	☐	☐	☐	☐	☐	☐	☐	☐	☐	☐
B7.8	Patients can see natural daylight	☐	☐	☐	☐	☐	☐	☐	☐	☐	☐
B7.9	Patients can experience quiet	☐	☐	☐	☐	☐	☐	☐	☐	☐	☐
B7.10	Patients can listen to TV or radio without disturbing others	☐	☐	☐	☐	☐	☐	☐	☐	☐	☐
B7.11	Patients can personalise their space	☐	☐	☐	☐	☐	☐	☐	☐	☐	☐
B7.12	Patients can turn the TV or radio on or off	☐	☐	☐	☐	☐	☐	☐	☐	☐	☐
B7.13	Patients can control the room temperature	☐	☐	☐	☐	☐	☐	☐	☐	☐	☐
B7.14	Patients can control the light in the room	☐	☐	☐	☐	☐	☐	☐	☐	☐	☐
B7.15	Patients can control the air in the room	☐	☐	☐	☐	☐	☐	☐	☐	☐	☐

Section C: Awareness that the patient was dying

C1	**Had the medical team diagnosed that this patient was dying?**	☐1 Yes	☐0 No

C2	**If yes, was this documented in the patient's hospital chart?**	☐1 Yes	☐0 No

		weeks	days	hours
C3	**If yes, how long before the patient's death were the medical team aware that the patient was dying?** (If this was less than a day, write the number of hours)			

C4	**Were the nursing staff aware that the patient was dying?**	☐1 Yes	☐0 No	☐9 Don't know

If the staff generally knew that the patient was dying, did any of these things happen?				
C5.1	**The multidisciplinary team (all health care professionals involved in the care of the patient) had a meeting to talk about and review the aims of the patient's care**	☐1 Yes	☐0 No	☐9 Don't know
C5.2	**The medical and nursing staff involved in the care of this patient had a meeting to talk about and review the aims of the patient's care**	☐1 Yes	☐0 No	☐9 Don't know
C5.3	**The patient or family was involved in the meeting to talk about and review the aims of the patient's care**	☐1 Yes	☐0 No	☐9 Don't know
C5.4	**The patient or family were told later about the result of the meeting to talk about and review the aims of the patient's care**	☐1 Yes	☐0 No	☐9 Don't know

C6	**Depending on the patient's condition during their last week of life, do you think the patient could have been allowed to die at home, if they got enough home care support?**	☐1 Yes	☐0 No	☐9 Don't know

Section D: Communication with patient

**Did staff on this ward have a discussion with the patient about any of these issues? If yes, is this discussion documented?**		**Did discussion happen?**		**Was discussion documented?**	
D1.1	**Patient's situation and prognosis**	☐1 Yes	☐0 No	☐1 Yes	☐0 No
D1.2	**The need to review aims of their care (curative or palliative)**	☐1 Yes	☐0 No	☐1 Yes	☐0 No
D1.3	**The need to review the patient's current treatment and its benefit versus burden**	☐1 Yes	☐0 No	☐1 Yes	☐0 No
D1.4	**What to expect as the patient's condition gets worse (when the patient has asked for this discussion)**	☐1 Yes	☐0 No	☐1 Yes	☐0 No
D1.5	**Getting the patient to talk about any of their concerns and / or preferences**	☐1 Yes	☐0 No	☐1 Yes	☐0 No
D1.6	**Active resuscitation of the patient**	☐1 Yes	☐0 No	☐1 Yes	☐0 No
D1.7	**Where the patient would prefer to be cared for**	☐1 Yes	☐0 No	☐1 Yes	☐0 No
D1.8	**Where the patient would prefer to die**	☐1 Yes	☐0 No	☐1 Yes	☐0 No

**If staff on this ward did not talk with the patient about the issues listed in D1, what was the reason for this?**				
D2.1	**The patient died suddenly or death was unexpected**	☐1 Yes	☐0 No	☐9 Don't know
D2.2	**The patient had dementia**	☐1 Yes	☐0 No	☐9 Don't know
D2.3	**The patient was too ill to have a discussion**	☐1 Yes	☐0 No	☐9 Don't know
D2.4	**There was no medical diagnosis or awareness that the patient was dying**	☐1 Yes	☐0 No	☐9 Don't know
D2.5	**The patient did not want to talk about dying**	☐1 Yes	☐0 No	☐9 Don't know
D2.6	**Relatives did not want the patient to be told that he or she was dying**	☐1 Yes	☐0 No	☐9 Don't know
D2.7	**There was not enough privacy on the ward to talk about dying**	☐1 Yes	☐0 No	☐9 Don't know
D2.8	**Medical staff had little experience or training to have a discussion of issues about dying and death**	☐1 Yes	☐0 No	☐9 Don't know
D2.9	**Nursing staff had little experience or training to have a discussion of issues about dying and death**	☐1 Yes	☐0 No	☐9 Don't know
D2.10	**Other reasons (write details):**	☐1 Yes	☐0 No	☐9 Don't know

If there was any discussion about the issues listed in D1, who started, and who took part in that discussion?					
		Started discussion?		Took part in discussion with the patient about any of the following?	
D3.1	**Doctor**	☐1 Yes	☐0 No	☐1 Yes	☐0 No
D3.2	**Specialist doctor**	☐1 Yes	☐0 No	☐1 Yes	☐0 No
D3.3	**Nurse**	☐1 Yes	☐0 No	☐1 Yes	☐0 No
D3.4	**Specialist nurse**	☐1 Yes	☐0 No	☐1 Yes	☐0 No
D3.5	**Patient**	☐1 Yes	☐0 No	☐1 Yes	☐0 No
D3.6	**Relative friend**	☐1 Yes	☐0 No	☐1 Yes	☐0 No

D4	If there was any discussion about the issues listed in D1, how long before the patient died did this discussion happen?	weeks	days	hours

If there was a discussion about the issues listed in D1, tell us what you think the patient felt about that discussion by rating these statements.		Poor									Excellent	Does not apply
		1	2	3	4	5	6	7	8	9	10	99
D5.1	**The discussion was sensitive to the patient's needs and understanding of his or her situation**	☐	☐	☐	☐	☐	☐	☐	☐	☐	☐	☐
D5.2	**The discussion was open and honest**	☐	☐	☐	☐	☐	☐	☐	☐	☐	☐	☐
D5.3	**The discussion was reassuring for the patient**	☐	☐	☐	☐	☐	☐	☐	☐	☐	☐	☐
D5.4	**The patient had an opportunity to talk about their concerns**	☐	☐	☐	☐	☐	☐	☐	☐	☐	☐	☐
D5.5	**The patient had an opportunity to talk about their preferences**	☐	☐	☐	☐	☐	☐	☐	☐	☐	☐	☐
D5.6	**The patient had an opportunity to ask questions**	☐	☐	☐	☐	☐	☐	☐	☐	☐	☐	☐
D5.7	**The patient was involved in making decisions about his or her care**	☐	☐	☐	☐	☐	☐	☐	☐	☐	☐	☐

If there was any discussion about the issues listed in D1, did the patient talk about their worries or wishes about dying or death? Were these documented in the patient's file?		Patient talked about worries or wishes?			Worries or wishes documented?	
		Yes	No	Don't know	Yes	No
D6.1	**Patient wanted to know how long he or she had to live**	☐1	☐0	☐9	☐1	☐0
D6.2	**Patient wanted their pain and other symptoms controlled**	☐1	☐0	☐9	☐1	☐0
D6.3	**Patient did not want to be actively resuscitated**	☐1	☐0	☐9	☐1	☐0

If there was any discussion about the issues listed in D1, did the patient talk about their worries or wishes about dying or death? Were these documented in the patient's file?		Patient talked about worries or wishes?			Worries or wishes documented?	
		Yes	No	Don't know	Yes	No
D6.4	Patient wanted to contact specific relatives before dying	☐1	☐0	☐9	☐1	☐0
D6.5	Patient said they preferred a single room	☐1	☐0	☐9	☐1	☐0
D6.6	Patient said they preferred to be cared for at home	☐1	☐0	☐9	☐1	☐0
D6.7	Patient was worried about being a financial burden on the family	☐1	☐0	☐9	☐1	☐0
D6.8	Patient wanted to have contact with pastoral care or their own spiritual adviser	☐1	☐0	☐9	☐1	☐0
D6.9	Patient wanted to make or change a will	☐1	☐0	☐9	☐1	☐0
D6.10	Patient wanted to arrange their funeral	☐1	☐0	☐9	☐1	☐0
D6.11	Patient wanted to donate his or her organs	☐1	☐0	☐9	☐1	☐0
D6.12	Other (write details):	☐1	☐0	☐9	☐1	☐0

If the patient talked about any worries or wishes (D6), in your view, how much did the hospital staff deal with any of these worries or wishes below?		Not at all ↓									Completely ↓	Does not apply
		1	2	3	4	5	6	7	8	9	10	99
D7.1	Patient wanted to know how long he or she had to live	☐	☐	☐	☐	☐	☐	☐	☐	☐	☐	☐
D7.2	Patient wanted their pain and other symptoms controlled	☐	☐	☐	☐	☐	☐	☐	☐	☐	☐	☐
D7.3	Patient did not want to be actively resuscitated	☐	☐	☐	☐	☐	☐	☐	☐	☐	☐	☐
D7.4	Patient wanted to contact specific relatives before dying	☐	☐	☐	☐	☐	☐	☐	☐	☐	☐	☐
D7.5	Patient said they preferred a single room	☐	☐	☐	☐	☐	☐	☐	☐	☐	☐	☐
D7.6	Patient said they preferred to be cared for at home	☐	☐	☐	☐	☐	☐	☐	☐	☐	☐	☐
D7.7	Patient was worried about being a financial burden on the family	☐	☐	☐	☐	☐	☐	☐	☐	☐	☐	☐
D7.8	Patient wanted to have contact with pastoral care or their own spiritual adviser	☐	☐	☐	☐	☐	☐	☐	☐	☐	☐	☐
D7.9	Patient wanted to make or change a will	☐	☐	☐	☐	☐	☐	☐	☐	☐	☐	☐
D7.10	Patient wanted to arrange their funeral	☐	☐	☐	☐	☐	☐	☐	☐	☐	☐	☐
D7.11	Patient wanted to donate his or her organs	☐	☐	☐	☐	☐	☐	☐	☐	☐	☐	☐
D7.12	Other (write details):	☐	☐	☐	☐	☐	☐	☐	☐	☐	☐	☐

D8	Did the patient document any preferences for their end-of-life care?	☐1 Yes	☐0 No

Section E: Communication with relatives or friends

Did staff on this ward talk to the relatives or friends about any of these issues? If yes, is this discussion documented?		Was there a discussion with the patient about any of the following?		Was discussion with the patient about any of the following documented?	
E1.1	Patient's situation and prognosis	☐1 Yes	☐0 No	☐1 Yes	☐0 No
E1.2	The need to review aims of the patient's curative or palliative care	☐1 Yes	☐0 No	☐1 Yes	☐0 No
E1.3	The need to review the patient's current treatment and its benefit versus burden	☐1 Yes	☐0 No	☐1 Yes	☐0 No
E1.4	What to expect as the patient's condition gets worse (when the patient has asked for this discussion)	☐1 Yes	☐0 No	☐1 Yes	☐0 No
E1.5	Getting the patient to talk about any worries and / or preferences	☐1 Yes	☐0 No	☐1 Yes	☐0 No
E1.6	Active resuscitation of the patient	☐1 Yes	☐0 No	☐1 Yes	☐0 No
E1.7	Where the patient would prefer to be cared for	☐1 Yes	☐0 No	☐1 Yes	☐0 No
E1.8	Where the patient's would prefer to die	☐1 Yes	☐0 No	☐1 Yes	☐0 No

If there was a discussion about any of the issues listed in E1:					
E2.1	Did the patient know about this discussion?	☐1 Yes	☐0 No	☐9 Don't know	☐99 No discussion
E2.2	Did the staff ask the patient's permission for this discussion?	☐1 Yes	☐0 No	☐9 Don't know	☐99 No discussion

If hospital staff did not talk about the possibility of dying with the patient's relatives or friends, what was the reason?				
E3.1	The patient died suddenly or their death was unexpected	☐1 Yes	☐0 No	☐9 Don't know
E3.2	There was no medical diagnosis or awareness that the patient was dying	☐1 Yes	☐0 No	☐9 Don't know
E3.3	The patient did not want relatives to know that he or she was dying	☐1 Yes	☐0 No	☐9 Don't know
E3.4	The relatives did not want to talk about the fact that the patient was dying	☐1 Yes	☐0 No	☐9 Don't know
E3.5	Not enough privacy on the ward to discuss dying	☐1 Yes	☐0 No	☐9 Don't know
E3.6	Medical staff had little experience or training to have a discussion of issues about dying and death	☐1 Yes	☐0 No	☐9 Don't know
E3.7	Nursing staff had little experience or training to have a discussion of issues about dying and death	☐1 Yes	☐0 No	☐9 Don't know
E3.8	Other reasons (write details):	☐1 Yes	☐0 No	☐9 Don't know

If there was any discussion about the issues listed in E1, who started it and who was part of that discussion?					
		**Started discussion?**		**Part of discussion with the patient about any of the following?**	
E4.1	**Doctor**	☐1 Yes	☐0 No	☐1 Yes	☐0 No
E4.2	**Specialist doctor**	☐1 Yes	☐0 No	☐1 Yes	☐0 No
E4.3	**Nurse**	☐1 Yes	☐0 No	☐1 Yes	☐0 No
E4.4	**Specialist nurse**	☐1 Yes	☐0 No	☐1 Yes	☐0 No
E4.5	**Patient**	☐1 Yes	☐0 No	☐1 Yes	☐0 No
E4.6	**Relative or friend**	☐1 Yes	☐0 No	☐1 Yes	☐0 No

E5	**If there was any discussion about the issues listed in E1, how long before the patient died did this discussion happen?**	weeks	days	hours

If there was any discussion about the issues listed in E1, tell us what you think the relatives felt about that discussion by rating these statements.		Poor ↓									Excellent ↓	Does not apply
		**1**	**2**	**3**	**4**	**5**	**6**	**7**	**8**	**9**	**10**	**99**
E6.1	**The discussion was sensitive to the needs of relatives and their understanding of the patient's situation**	☐	☐	☐	☐	☐	☐	☐	☐	☐	☐	☐
E6.2	**The discussion was open and honest**	☐	☐	☐	☐	☐	☐	☐	☐	☐	☐	☐
E6.3	**The discussion was reassuring for relatives**	☐	☐	☐	☐	☐	☐	☐	☐	☐	☐	☐
E6.4	**The relatives had an opportunity to talk about their worries**	☐	☐	☐	☐	☐	☐	☐	☐	☐	☐	☐
E6.5	**The relatives had an opportunity to talk about their preferences**	☐	☐	☐	☐	☐	☐	☐	☐	☐	☐	☐
E6.6	**The relatives had an opportunity to ask questions**	☐	☐	☐	☐	☐	☐	☐	☐	☐	☐	☐
E6.7	**The relatives were appropriately involved in decisions about the patient's care**	☐	☐	☐	☐	☐	☐	☐	☐	☐	☐	☐

If there was any discussion about the issues listed in E1, did relatives talk about their worries or wishes about dying or death, such as these? Were these documented in the patient's chart?		The relatives talk about their worries or wishes?			Worries or wishes documented?	
		Yes	No	Don't know	Yes	No
E7.1	Relatives wanted a single room for the patient	☐1	☐0	☐9	☐1	☐0
E7.2	Relatives were worried about the patient's pain and overall symptom management	☐1	☐0	☐9	☐1	☐0
E7.3	Relatives wanted to be told if the patient's condition deteriorated	☐1	☐0	☐9	☐1	☐0
E7.4	Relatives wanted to be told if the patient's death was very soon	☐1	☐0	☐9	☐1	☐0
E7.5	Relatives wanted more information about the process of dying	☐1	☐0	☐9	☐1	☐0
E7.6	Relatives were worried about active resuscitation	☐1	☐0	☐9	☐1	☐0
E7.7	Relatives wanted to stay by the patient's bedside	☐1	☐0	☐9	☐1	☐0
E7.8	Relatives asked for a religious service or prayers at the patient's bedside	☐1	☐0	☐9	☐1	☐0
E7.9	Relatives wanted the patient's organs to be donated	☐1	☐0	☐9	☐1	☐0
E7.10	Relatives wanted advice on how to arrange a funeral	☐1	☐0	☐9	☐1	☐0
E7.11	Relatives wanted support on how to deal with their grief	☐1	☐0	☐9	☐1	☐0
E7.12	Other (write details):	☐1	☐0	☐9	☐1	☐0

If relatives talked about their worries or wishes (E7), how much did the hospital staff deal with any of these?		Not at all ↓									Completely ↓	Does not apply
		1	2	3	4	5	6	7	8	9	10	99
E8.1	Relatives wanted a single room for the patient	☐	☐	☐	☐	☐	☐	☐	☐	☐	☐	☐
E8.2	Relatives said they were worried about the patient's pain and overall symptom management	☐	☐	☐	☐	☐	☐	☐	☐	☐	☐	☐
E8.3	Relatives wanted to be told if the patient's condition deteriorated	☐	☐	☐	☐	☐	☐	☐	☐	☐	☐	☐
E8.4	Relatives wanted to be told if the patient's death was very soon	☐	☐	☐	☐	☐	☐	☐	☐	☐	☐	☐
E8.5	Relatives wanted more information about the process of dying	☐	☐	☐	☐	☐	☐	☐	☐	☐	☐	☐
E8.6	Relatives said they were worried about active resuscitation	☐	☐	☐	☐	☐	☐	☐	☐	☐	☐	☐
E8.7	Relatives wanted to stay by the patient's bedside	☐	☐	☐	☐	☐	☐	☐	☐	☐	☐	☐
E8.8	Relatives asked for a religious service or prayers at the patient's bedside	☐	☐	☐	☐	☐	☐	☐	☐	☐	☐	☐
E8.9	Relatives wanted the patient's organs to be donated	☐	☐	☐	☐	☐	☐	☐	☐	☐	☐	☐
E8.10	Relatives wanted advice on how to arrange a funeral	☐	☐	☐	☐	☐	☐	☐	☐	☐	☐	☐
E8.11	Relatives wanted support on how to deal with their grief	☐	☐	☐	☐	☐	☐	☐	☐	☐	☐	☐
E8.12	Other (write details):	☐	☐	☐	☐	☐	☐	☐	☐	☐	☐	☐

E9.1	**Were any relatives offered the opportunity to stay overnight in the hospital?**	☐1 Yes	☐0 No
E9.2	**Did any relatives stay overnight in the hospital?**	☐1 Yes	☐0 No
E9.3	**Were relatives free to visit at any time?**	☐1 Yes	☐0 No
E9.4	**Were relatives offered free food and drink?**	☐1 Yes	☐0 No
E9.5	**Were relatives offered preferential or free car parking?**	☐1 Yes	☐0 No
E9.6	**Were relatives offered information leaflets on dying, death and bereavement?**	☐1 Yes	☐0 No

Section F: Decisions about treatment

At any time during the patient's last week of life, was there a decision to do any of these?		Was a decision made?			Was the decision documented?		
		Yes	No	Does not apply*	Yes	No	Does not apply*
F1.1	**Review whether the aim of care was curative or palliative**	☐1	☐0	☐9	☐1	☐0	☐9
F1.2	**Change or start medical treatments to optimise comfort and symptom management of the patient**	☐1	☐0	☐9	☐1	☐0	☐9
F1.3	**Review medication, route of administration, and stop non-essential medication**	☐1	☐0	☐9	☐1	☐0	☐9
F1.4	**Stop blood tests that have no effect on treatment or care**	☐1	☐0	☐9	☐1	☐0	☐9
F1.5	**Stop antibiotics if they were not adding to comfort or supportive care**	☐1	☐0	☐9	☐1	☐0	☐9
F1.6	**Review artificial hydration (IV or SC fluids) in terms of benefit versus burden**	☐1	☐0	☐9	☐1	☐0	☐9
F1.7	**Stop invasive monitoring**	☐1	☐0	☐9	☐1	☐0	☐9
F1.8	**Withhold treatment, for example a decision not to start or increase a life-sustaining intervention**	☐1	☐0	☐9	☐1	☐0	☐9
F1.9	**Withdraw treatment, for example a decision to stop or decrease a life-sustaining intervention**	☐1	☐0	☐9	☐1	☐0	☐9
F1.10	**Talk about and record resuscitation status with patient or relative**	☐1	☐0	☐9	☐1	☐0	☐9
F1.11	**Assess skin integrity of patient and take appropriate measures (for example, electric bed or mattress)**	☐1	☐0	☐9	☐1	☐0	☐9

*This question does not apply if the patient's death was sudden or unexpected.

During their last week of life, patients sometimes have these symptoms. For each symptom, how often the patient had this in their last week of life?						If the patient had this symptom at any time during their last week of life, how well it was managed by the hospital team to keep the patient comfortable? For each symptom, rate how well the team managed it on this scale. If the patient did not have this symptom, tick the NA (Not applicable) box.										
		All the time	Most of the time	Some of the time	None of the time	Very badly ↓									Excellent ↓	NA
						1	2	3	4	5	6	7	8	9	10	99
F2.1	**Pain**	☐1	☐2	☐3	☐4	☐	☐	☐	☐	☐	☐	☐	☐	☐	☐	☐
F2.2	**Nausea and / or vomiting**	☐1	☐2	☐3	☐4	☐	☐	☐	☐	☐	☐	☐	☐	☐	☐	☐
F2.3	**Breathing difficulties**	☐1	☐2	☐3	☐4	☐	☐	☐	☐	☐	☐	☐	☐	☐	☐	☐
F2.4	**Increased secretions**	☐1	☐2	☐3	☐4	☐	☐	☐	☐	☐	☐	☐	☐	☐	☐	☐
F2.5	**Restlessness or agitation**	☐1	☐2	☐3	☐4	☐	☐	☐	☐	☐	☐	☐	☐	☐	☐	☐
F2.6	**Anxiety or fear**	☐1	☐2	☐3	☐4	☐	☐	☐	☐	☐	☐	☐	☐	☐	☐	☐

Section G: Specialist palliative care

G1	**Is there a specialist palliative care service available in this hospital?**	☐1 Yes	☐0 No	☐9 Don't know

If there is <u>no</u> palliative care service, go to Section H.

G2	**Did the patient get any contribution from a specialist palliative care service after admission to hospital?**	☐1 Yes	☐0 No	☐9 Don't know

		Weeks	Days	Hours
G3	**If yes, how long before death did the patient get this contribution from a specialist palliative care service?** (If less than a day, write the number of hours)			

G4	**If the patient did <u>not</u> get any contribution from a specialist palliative care service, or if there is no such service in the hospital, do you think he or she might have benefited from the input of a specialist palliative care service?**	☐1 Yes	☐0 No	☐9 Don't know

Section H: Quality of life in the last week

These questions are about the patient's experience during their last week of life. Answer each question, even if the patient was unconscious for some or all of the time.

Base your answer on what you think, rather than how you think your patient would have rated this experience. If you do not know enough to rate the experience, then tick the 'Don't know' box.

H1a	**Did the patient have physical pain?**	None of the time	A little bit of the time	Some of the time	A good bit of the time	Most of the time	All of the time	Don't know
		☐1	☐2	☐3	☐4	☐5	☐6	☐9

H1b	**How would you rate this part of the patient's experience?**	Unsatisfactory ↓									Satisfactory ↓	Don't know
		☐1	☐2	☐3	☐4	☐5	☐6	☐7	☐8	☐9	☐10	☐99

H2a	**Was the patient able to eat or drink?**	None of the time	A little bit of the time	Some of the time	A good bit of the time	Most of the time	All of the time	Don't know
		☐1	☐2	☐3	☐4	☐5	☐6	☐9

H2b	**How would you rate this part of the patient's experience?**	Unsatisfactory ↓									Satisfactory ↓	Not Applicable
		☐1	☐2	☐3	☐4	☐5	☐6	☐7	☐8	☐9	☐10	☐99

H3a	**Did the patient have breathing problems?**	None of the time	A little bit of the time	Some of the time	A good bit of the time	Most of the time	All of the time	Don't know
		☐1	☐2	☐3	☐4	☐5	☐6	☐9

H3b	**How would you rate this part of the patient's experience?**	Unsatisfactory ↓									Satisfactory ↓	Don't know
		☐1	☐2	☐3	☐4	☐5	☐6	☐7	☐8	☐9	☐10	☐99

H4a	**Did the patient seem comfortable and at ease?**	None of the time	A little bit of the time	Some of the time	A good bit of the time	Most of the time	All of the time	Don't know
		☐1	☐2	☐3	☐4	☐5	☐6	☐9

H4b	**How would you rate this part of the patient's experience?**	Unsatisfactory ↓									Satisfactory ↓	Don't know
		☐1	☐2	☐3	☐4	☐5	☐6	☐7	☐8	☐9	☐10	☐99

H5a	**Did the patient seem afraid or anxious?**	None of the time	A little bit of the time	Some of the time	A good bit of the time	Most of the time	All of the time	Don't know
		☐1	☐2	☐3	☐4	☐5	☐6	☐9

H5b	How would you rate this part of the patient's experience?	Unsatisfactory ⬇									Satisfactory ⬇	Don't know
		☐1	☐2	☐3	☐4	☐5	☐6	☐7	☐8	☐9	☐10	☐99

H6a	Did the patient smile, laugh or show any signs of enjoyment in their last week?	None of the time	A little bit of the time	Some of the time	A good bit of the time	Most of the time	All of the time	Don't know
		☐1	☐2	☐3	☐4	☐5	☐6	☐9

H6b	How would you rate this part of the patient's experience?	Unsatisfactory ⬇									Satisfactory ⬇	Don't know
		☐1	☐2	☐3	☐4	☐5	☐6	☐7	☐8	☐9	☐10	☐99

H7a	Did the patient seem to have the energy to do most of the things that he or she wanted to do?	None of the time	A little bit of the time	Some of the time	A good bit of the time	Most of the time	All of the time	Don't know
		☐1	☐2	☐3	☐4	☐5	☐6	☐9

H7b	How would you rate this part of the patient's experience?	Unsatisfactory ⬇									Satisfactory ⬇	Don't know
		☐1	☐2	☐3	☐4	☐5	☐6	☐7	☐8	☐9	☐10	☐99

H8a	Could the patient control physically when they went to the toilet?	None of the time	A little bit of the time	Some of the time	A good bit of the time	Most of the time	All of the time	Don't know
		☐1	☐2	☐3	☐4	☐5	☐6	☐9

H8b	How would you rate this part of the patient's experience?	Unsatisfactory ⬇									Satisfactory ⬇	Don't know
		☐1	☐2	☐3	☐4	☐5	☐6	☐7	☐8	☐9	☐10	☐99

H9a	Did the patient seem worried about causing strain to his or her loved ones?	None of the time	A little bit of the time	Some of the time	A good bit of the time	Most of the time	All of the time	Don't know
		☐1	☐2	☐3	☐4	☐5	☐6	☐9

H9b	How would you rate this part of the patient's experience?	Unsatisfactory ⬇									Satisfactory ⬇	Don't know
		☐1	☐2	☐3	☐4	☐5	☐6	☐7	☐8	☐9	☐10	☐99

H10a	Was the patient's dignity and self-respect maintained?	None of the time	A little bit of the time	Some of the time	A good bit of the time	Most of the time	All of the time	Don't know
		☐1	☐2	☐3	☐4	☐5	☐6	☐9

H10b	How would you rate this part of the patient's experience?	Unsatisfactory ⬇									Satisfactory ⬇	Don't know
		☐1	☐2	☐3	☐4	☐5	☐6	☐7	☐8	☐9	☐10	☐99

If the patient had a living husband, wife or partner:		None of the time	A little bit of the time	Some of the time	A good bit of the time	Most of the time	All of the time	Don't know
H11a	Was the patient helped to spend time with his or her husband, wife or partner in the way they wanted to?	☐1	☐2	☐3	☐4	☐5	☐6	☐9

If the patient had a living husband, wife or partner:		Unsatisfactory ↓									Satisfactory ↓	Don't know
H11b	How would you rate this part of the patient's experience?	☐1	☐2	☐3	☐4	☐5	☐6	☐7	☐8	☐9	☐10	☐99

If the patient had children:		None of the time	A little bit of the time	Some of the time	A good bit of the time	Most of the time	All of the time	Don't know
H12a	Was the patient helped to spend time with his or her children in the way they wanted to?	☐1	☐2	☐3	☐4	☐5	☐6	☐9

If the patient had children:		Unsatisfactory ↓									Satisfactory ↓	Don't know
H12b	How would you rate this part of the patient's experience?	☐1	☐2	☐3	☐4	☐5	☐6	☐7	☐8	☐9	☐10	☐99

H13a	Was the patient helped to spend time with family members and friends in the way they wanted to?	None of the time	A little bit of the time	Some of the time	A good bit of the time	Most of the time	All of the time	Don't know
		☐1	☐2	☐3	☐4	☐5	☐6	☐9

H13b	How would you rate this part of the patient's experience?	Unsatisfactory ↓									Satisfactory ↓	Don't know
		☐1	☐2	☐3	☐4	☐5	☐6	☐7	☐8	☐9	☐10	☐99

H14a	Was the patient helped to spend time alone in the way he or she wanted to?	None of the time	A little bit of the time	Some of the time	A good bit of the time	Most of the time	All of the time	Don't know
		☐1	☐2	☐3	☐4	☐5	☐6	☐9

H14b	How would you rate this part of the patient's experience?	Unsatisfactory ↓									Satisfactory ↓	Don't know
		☐1	☐2	☐3	☐4	☐5	☐6	☐7	☐8	☐9	☐10	☐99

H15a	Did the patient appear to have meaning and purpose in their last week of life?	☐1 Yes	☐0 No	☐9 Don't know

H15b	How would you rate this part of the patient's experience?	Unsatisfactory ↓									Satisfactory ↓	Don't know
		☐1	☐2	☐3	☐4	☐5	☐6	☐7	☐8	☐9	☐10	☐99

H16a	**Did the patient know that his or her loved ones were there in their last week of life?**	☐1 Yes	☐0 No	☐9 Don't know

H16b	**How would you rate this part of the patient's experience?**	Unsatisfactory ↓									Satisfactory ↓	Don't know
		☐1	☐2	☐3	☐4	☐5	☐6	☐7	☐8	☐9	☐10	☐99

H17a	**Did the patient have any money worries, such as the cost of health care?**	☐1 Yes	☐0 No	☐9 Don't know

H17b	**How would you rate this part of the patient's experience?**	Unsatisfactory ↓									Satisfactory ↓	Don't know
		☐1	☐2	☐3	☐4	☐5	☐6	☐7	☐8	☐9	☐10	☐99

H18a	**Did the patient have an opportunity to say goodbye to their loved ones?**	☐1 Yes	☐0 No	☐9 Don't know

H18b	**How would you rate this part of the patient's experience?**	Unsatisfactory ↓									Satisfactory ↓	Don't know
		☐1	☐2	☐3	☐4	☐5	☐6	☐7	☐8	☐9	☐10	☐99

H19a	**Did the patient have one or more visits from a religious or spiritual advisor, such as a priest?**	☐1 Yes	☐0 No	☐9 Don't know

H19b	**How would you rate this part of the patient's experience?**	Unsatisfactory ↓									Satisfactory ↓	Don't know
		☐1	☐2	☐3	☐4	☐5	☐6	☐7	☐8	☐9	☐10	☐99

H20a	**Did the patient have a spiritual service or ceremony before his or her death?**	☐1 Yes	☐0 No	☐9 Don't know

H20b	**How would you rate this part of the patient's experience?**	Unsatisfactory ↓									Satisfactory ↓	Don't know
		☐1	☐2	☐3	☐4	☐5	☐6	☐7	☐8	☐9	☐10	☐99

H21a	**Was anyone with the patient at the moment of his or her death?**	☐1 Yes	☐0 No	☐9 Don't know

H21b	**How would you rate this part of the patient's experience?**	Unsatisfactory ↓									Satisfactory ↓	Don't know
		☐1	☐2	☐3	☐4	☐5	☐6	☐7	☐8	☐9	☐10	☐99

H22a	**In the moment before the patient died, was he or she ...**	alert	semi-conscious but could speak	unconscious	Don't know
		☐1	☐2	☐3	☐9

H22b	**How would you rate this part of the patient's experience?**	Unsatisfactory ↓									Satisfactory ↓	Don't know
		☐1	☐2	☐3	☐4	☐5	☐6	☐7	☐8	☐9	☐10	☐99

H23	Overall, how would you rate the patient's quality of the moment of death?	Unsatisfactory ↓									Satisfactory ↓	Don't know
		☐1	☐2	☐3	☐4	☐5	☐6	☐7	☐8	☐9	☐10	☐99

H24	Overall, how would you rate the patient's quality of life during their last week?	Unsatisfactory ↓									Satisfactory ↓	Don't know
		☐1	☐2	☐3	☐4	☐5	☐6	☐7	☐8	☐9	☐10	☐99

H25	Overall, how would you rate the patient's quality of dying during their last week?	Unsatisfactory ↓									Satisfactory ↓	Don't know
		☐1	☐2	☐3	☐4	☐5	☐6	☐7	☐8	☐9	☐10	☐99

Section J: Quality of care

J1	How well do you think the hospital team communicated with the patient about his or her situation and their likely prognosis?	Not well ↓									Very well ↓	Don't know
		☐1	☐2	☐3	☐4	☐5	☐6	☐7	☐8	☐9	☐10	☐99

J2	How well do you think the hospital team provided end-of-life care that respected the patient's wishes?	Not well ↓									Very well ↓	Don't know
		☐1	☐2	☐3	☐4	☐5	☐6	☐7	☐8	☐9	☐10	☐99

J3	How well do you think the hospital team communicated with the relatives or friends about the patient's illness and the likely prognosis?	Not well ↓									Very well ↓	Don't know
		☐1	☐2	☐3	☐4	☐5	☐6	☐7	☐8	☐9	☐10	☐99

J4	How well do you think the hospital team managed the patient's symptoms, such as pain, to a level that was acceptable to him or her?	Not well ↓									Very well ↓	Don't know
		☐1	☐2	☐3	☐4	☐5	☐6	☐7	☐8	☐9	☐10	☐99

J5	How well do you think the hospital team gave emotional support for the family or friends of the patient?	Not well ↓									Very well ↓	Don't know
		☐1	☐2	☐3	☐4	☐5	☐6	☐7	☐8	☐9	☐10	☐99

Who was with the patient at the moment of their death?				
J6.1	Relatives or friends	☐1 Yes	☐0 No	☐9 Don't know
J6.2	Hospital staff	☐1 Yes	☐0 No	☐9 Don't know
J6.3	Other (write details):	☐1 Yes	☐0 No	☐9 Don't know

J7	At the time of the patient's death, or immediately after their death, was anyone from the pastoral care team present?	At time of death	Immediately after death	No one from pastoral care team	No pastoral care team
		☐1	☐2	☐0	☐9

Do you feel the way this patient died in hospital would be acceptable for you, or for your family or friends?		Definitely not acceptable ↓								Very acceptable ↓	Don't know
		1	2	3	4	5	6	7	8	9	10	0
J8.1	**Acceptable for you?**	☐	☐	☐	☐	☐	☐	☐	☐	☐	☐	☐
J8.2	**Acceptable for your family or friends?**	☐	☐	☐	☐	☐	☐	☐	☐	☐	☐	☐

Section K: Time after death

**In the time after the patient's death, were any rituals held in the ward or room around the body to mark the death, such as these?**		A) Ritual held in the ward or room immediately after the moment of patient's death?			B) Ritual held in the ward or room when the body was being taken away?		
		Yes	No	Don't know	Yes	No	Don't know
K1.1	**Prayers were said**	☐1	☐0	☐9	☐1	☐0	☐9
K1.2	**A moment of silence was held**	☐1	☐0	☐9	☐1	☐0	☐9
K1.3	**Candles were lit**	☐1	☐0	☐9	☐1	☐0	☐9
K1.4	**Sympathy was offered to the family**	☐1	☐0	☐9	☐1	☐0	☐9
K1.5	**Tea was offered**	☐1	☐0	☐9	☐1	☐0	☐9
K1.6	**Other:**	☐1	☐0	☐9	☐1	☐0	☐9

K2	**Immediately after the patient's death, were relatives given all the time they wanted to be with the patient who has died?**	☐1 Yes	☐0 No

K3	**Immediately after the patient's death, did staff have enough time to be available for the relatives?**	☐1 Yes	☐0 No

**Which hospital staff went with the body to the mortuary?**				
K4.1	**Porter**	☐1 Yes	☐0 No	☐9 Don't know
K4.2	**Nurse**	☐1 Yes	☐0 No	☐9 Don't know
K4.3	**Other**	☐1 Yes	☐0 No	☐9 Don't know

At any time after the patient's death, were relatives offered any of these kinds of information or advice?		Yes	No	Don't know	Not needed or not relevant
K5.1	**Information on how the person who has died will be moved to the mortuary**	☐1	☐0	☐9	☐99
K5.2	**Information on how the person who has died may be taken home**	☐1	☐0	☐9	☐99
K5.3	**Information on mortuary access and viewing times**	☐1	☐0	☐9	☐99
K5.4	**Information on arranging the funeral or contacting funeral directors**	☐1	☐0	☐9	☐99
K5.5	**Information on collecting the patient's personal belongings**	☐1	☐0	☐9	☐99
K5.6	**Information on how to register the death**	☐1	☐0	☐9	☐99
K5.7	**Written information on post-mortems**	☐1	☐0	☐9	☐99
K5.8	**Written information on bereavement and bereavement services**	☐1	☐0	☐9	☐99
K5.9	**Other information (write details)**	☐1	☐0	☐9	☐99

K6	**Did hospital staff tell the GP of the patient's death?**	☐1 Yes	☐0 No	☐9 Don't know

K7.1	**How much time was there between the time of patient's death and a doctor certifying that the patient was dead?**	hours	days	☐9 Don't know

K7.2	**How much time was there between the time of death and the issuing of the death notification certificate by the hospital?**	hours	days	☐9 Don't know

Section L: Personal belongings of patient

L1	**Who gathered the personal belongings of the patient in the hospital after he or she died?**	Nurse	Care assistant	Relative	Other person
		☐1	☐2	☐3	☐4

L2	**What type of bag or container held the personal belongings of the patient after he or she died?**	Patient's own bag	Bag supplied by relative	Plastic bag	Family hand-over bag	Other
		☐1	☐2	☐3	☐4	☐5

Section M: Review of patient's dying and death

M1	**Was there any discussion at ward level about how the dying and death of this patient was managed?**	☐1 Yes	☐0 No	☐9 Don't know

If yes, was this:				
M2.1	**A formal discussion facilitated by a senior member of staff?**	☐1 Yes	☐0 No	☐9 Don't know
M2.2	**An informal discussion where staff spoke with their peers?**	☐1 Yes	☐0 No	☐9 Don't know

M3	**Did any staff involved in the care of this patient feel very upset after the patient's death?**	☐1 Yes	☐0 No	☐9 Don't know

M4	**If yes, did these staff have the opportunity to talk about how this patient's death affected them?**	☐1 Yes	☐0 No	☐9 Don't know

		Yes, in the hospital	Yes, outside the hospital	No discussion	Don't know
M5	**If yes, where did the discussion take place?**	☐1	☐2	☐0	☐9

M6.1 **What went well in the care of this patient during their last week of life?**

____

____

____

M6.2 **What did not go well in the care of this patient during their last week of life?**

____

____

____

M6.3 **What could have been done better in the care of this patient during their last week of life?**

____

____

____

Part two: **Nurse's perceptions**

Section A: Background

A1	**What is your position in the ward?**	Clinical nurse manager, CNS, ANP, etc. ☐1	Staff nurse ☐2	Other ☐3

A2	**How long have you been working in this hospital?**	(to the nearest year)

A3	**How long have you been working on this ward?**	(to the nearest year)

A4	**Where were you brought up?**	Australia ☐1	India ☐2	Ireland ☐3	Middle East ☐4	Nigeria ☐5	New Zealand ☐6
		Pakistan ☐7	Philippines ☐8	South Africa ☐9	UK ☐10	US ☐11	Other ☐12

A5	**Are you male or female?**	☐1 Male	☐2 Female

A6	**How old are you?**	(to the nearest year)

A7	**Is English your first language?**	☐1 Yes	☐2No

Section B: General attitudes to dying and death

		Not at all comfortable	Not very comfortable	Relatively comfortable	Very comfortable	Completely comfortable	Don't know
B1	**How comfortable are you personally with talking about death or dying?**	☐1	☐2	☐3	☐4	☐5	☐9
B2	**How comfortable are you personally with talking to people who have been recently bereaved?**	☐1	☐2	☐3	☐4	☐5	☐9

B3	**Where would you want to be cared for if you were dying?**	In a hospital ☐1	In my own home ☐2	In a hospice ☐3	Nursing Home ☐4	Other ☐5	Don't know ☐9

B4	**How would you describe the overall care that is given to people who die in Irish hospitals?**	Very poor ☐1	Poor ☐2	Fair ☐3	Good ☐4	Excellent ☐5	Don't know ☐9

Section C: Characteristics of ward

C1	**How would you describe the bed occupancy rate of this ward?**	Very low ☐1	Low ☐2	Average ☐3	High ☐4	Very high ☐5

C2	**How would you describe patient turnover rates in this ward?**	Very low ☐1	Low ☐2	Average ☐3	High ☐4	Very high ☐5

C3	**How would you describe patient dependency levels in this ward?**	Very low ☐1	Low ☐2	Average ☐3	High ☐4	Very high ☐5

C4	**How would you describe the level of staff turnover in this ward?**	Very low ☐1	Low ☐2	Average ☐3	High ☐4	Very high ☐5

C5	**In your opinion, are there enough nursing staff in this ward?**	Definitely not enough	Not enough	Just enough	Definitely enough
		☐1	☐2	☐3	☐4

C6	**How often does a patient die on this ward?**	Nearly every day ☐1	Nearly every week ☐2	Nearly every two weeks ☐3	Nearly every three weeks ☐4	Nearly every month ☐5	Less than once a month ☐9

Section D: Working environment

**In general, how would you rate these aspects of this ward?**		Very poor ↓ 1	2	3	4	5	6	7	8	9	Excellent ↓ 10
D1	**What is the ward like as a place to work?**	☐	☐	☐	☐	☐	☐	☐	☐	☐	☐
D2	**What is the quality of relationships between all staff on this ward?**	☐	☐	☐	☐	☐	☐	☐	☐	☐	☐
D3	**How well equipped is this ward, such as facilities, equipment etc.?**	☐	☐	☐	☐	☐	☐	☐	☐	☐	☐
D4	**What is the standard of care given to patients on this ward?**	☐	☐	☐	☐	☐	☐	☐	☐	☐	☐
D5	**What is this ward like as a place to deliver end-of-life care?**	☐	☐	☐	☐	☐	☐	☐	☐	☐	☐
D6	**What is the quality of ward management?**	☐	☐	☐	☐	☐	☐	☐	☐	☐	☐

		Very dissatisfied ↓ 1	2	3	4	5	6	7	8	9	Very satisfied ↓ 10
D7	**Overall, how satisfied are you with your current work situation?**	☐	☐	☐	☐	☐	☐	☐	☐	☐	☐

Section E: End-of-life care on this ward

From your experience of working in this ward, how would you rate these parts of the ward's end-of-life care?		Very poor 1	2	3	4	5	6	7	8	9	Very good 10
E1	Recognising when a patient needs palliative care rather than curative care	☐	☐	☐	☐	☐	☐	☐	☐	☐	☐
E2	Communicating with patients and relatives in a sensitive, truthful and reassuring way	☐	☐	☐	☐	☐	☐	☐	☐	☐	☐
E3	Communicating and sharing information effectively among hospital staff	☐	☐	☐	☐	☐	☐	☐	☐	☐	☐
E4	Making sure that the patient's end-of-life care is coordinated	☐	☐	☐	☐	☐	☐	☐	☐	☐	☐
E5	Giving patients an opportunity to talk about their worries and wishes	☐	☐	☐	☐	☐	☐	☐	☐	☐	☐
E6	Giving relatives or friends an opportunity to talk about their worries and wishes	☐	☐	☐	☐	☐	☐	☐	☐	☐	☐
E7	Making sure the patient's preferences are respected	☐	☐	☐	☐	☐	☐	☐	☐	☐	☐
E8	Making sure the patient is comfortable, and properly managing their pain and other symptoms	☐	☐	☐	☐	☐	☐	☐	☐	☐	☐
E9	Comforting a patient who is afraid of dying	☐	☐	☐	☐	☐	☐	☐	☐	☐	☐
E10	Supporting relatives or friends to spend time with the dying patient	☐	☐	☐	☐	☐	☐	☐	☐	☐	☐
E11	Creating a sense of dignity and respect around the moment of the patient's death	☐	☐	☐	☐	☐	☐	☐	☐	☐	☐
E12	Respecting the spiritual needs of people from different religious traditions about death	☐	☐	☐	☐	☐	☐	☐	☐	☐	☐
E13	Removing the person who has died respectfully from the ward	☐	☐	☐	☐	☐	☐	☐	☐	☐	☐
E14	Providing a mortuary that respects the dead	☐	☐	☐	☐	☐	☐	☐	☐	☐	☐
E15	Supporting bereaved relatives with information, advice and counselling as they need it	☐	☐	☐	☐	☐	☐	☐	☐	☐	☐
E16	Having clear policies and procedures for end-of-life care	☐	☐	☐	☐	☐	☐	☐	☐	☐	☐

Based on your experience of working in this ward, do you feel the way patients die in this ward would be acceptable for you, or for your family or friends?		Definitely not acceptable 1	2	3	4	5	6	7	8	9	Very acceptable 10
E17.1	Acceptable for you?	☐	☐	☐	☐	☐	☐	☐	☐	☐	☐
E17.2	Acceptable for your family or friends?	☐	☐	☐	☐	☐	☐	☐	☐	☐	☐

E18 **Could you suggest one thing that would make the end-of-life care in this ward much better?**

____

____

____

Section F: Professional and personal preparation for end-of-life care

F1	**Since qualifying have you gone on a formal training course on end-of-life care or palliative care?**	☐1 Yes	☐0 No

**If yes:**			
F2.1	**What was the name of the course?**		
F2.2	**How long did the course last?**	days	hours
F2.3	**Was the course given by the hospital where you currently work?**	☐1 Yes	☐0 No

F3	**How prepared do you feel, professionally, for dealing with the death of a patient?**	Completely unprepared ☐1	Fairly unprepared ☐2	Reasonably prepared ☐3	Completely prepared ☐4

F4	**How prepared do you feel, personally or emotionally, for dealing with the death of a patient?**	Completely unprepared ☐1	Fairly unprepared ☐2	Reasonably prepared ☐3	Completely prepared ☐4

Section G: Experiences after the death of a patient

G1	**In the past year, have you been very upset after a patient's death?**	☐1 Yes	☐0 No

G2	**If yes, did you feel like you needed to talk to someone about it?**	☐1 Yes	☐0 No

G3	**If yes, did you talk to someone?**	Yes, to someone in the hospital	Yes, to someone outside the hospital	No
		☐1	☐2	☐0

**In the future if you were very upset after the death of a patient, what supports could you get?**				
G4.1	**Counselling organised by hospital**	☐1 Yes	☐0 No	☐9 Don't know
G4.2	**Colleagues are available and willing to talk and listen to me**	☐1 Yes	☐0 No	☐9 Don't know
G4.3	**Managers are available and willing to talk and listen to me**	☐1 Yes	☐0 No	☐9 Don't know
G4.4	**I could change shift patterns**	☐1 Yes	☐0 No	☐9 Don't know
G4.5	**I could take time off work**	☐1 Yes	☐0 No	☐9 Don't know
G4.6	**Other support (write details):**	☐1 Yes	☐0 No	☐9 Don't know

Section H: Education, training and other supports for end-of-life care

From your experience of working in this hospital, rate these supports offered to staff who are dealing with end-of-life care?		Inadequate ↓									Adequate ↓	Don't know
		1	2	3	4	5	6	7	8	9	10	0
H1	**Hospital offers training on the care of patients and family at the end-of-life**	☐	☐	☐	☐	☐	☐	☐	☐	☐	☐	☐
H2	**Hospital offers training in communication skills about dying, death, and bereavement, including breaking bad news to people**	☐	☐	☐	☐	☐	☐	☐	☐	☐	☐	☐
H3	**Hospital offers training in what people from different cultures expect at death**	☐	☐	☐	☐	☐	☐	☐	☐	☐	☐	☐
H4	**Hospital offers courses on understanding the effects of loss, grief and bereavement on people**	☐	☐	☐	☐	☐	☐	☐	☐	☐	☐	☐
H5	**Hospital offers courses on understanding the legal and ethical issues around end-of-life care**	☐	☐	☐	☐	☐	☐	☐	☐	☐	☐	☐
H6	**Hospital offers specialist knowledge and support through its palliative care service**	☐	☐	☐	☐	☐	☐	☐	☐	☐	☐	☐
H7	**Hospital offers opportunities for debriefing, reflection and counseling**	☐	☐	☐	☐	☐	☐	☐	☐	☐	☐	☐
H8	**Hospital encourages positive inter-disciplinary team working**	☐	☐	☐	☐	☐	☐	☐	☐	☐	☐	☐
H9	**Hospital holds post-death reviews**	☐	☐	☐	☐	☐	☐	☐	☐	☐	☐	☐
H10	**Managers show leadership in making end-of-life care better**	☐	☐	☐	☐	☐	☐	☐	☐	☐	☐	☐
H11	**Hospital has clear policies and procedures on dying, death and bereavement**	☐	☐	☐	☐	☐	☐	☐	☐	☐	☐	☐

Section J: Hospital priorities

From your experience of working in this hospital, how much attention does the hospital pay to these things?		Very little attention ↓ 1	2	3	4	5	6	7	8	9	A lot of attention ↓ 10	Don't know 0
J1	**Active treatment of illness**	☐	☐	☐	☐	☐	☐	☐	☐	☐	☐	☐
J2	**Optimising the quality of life for each patient**	☐	☐	☐	☐	☐	☐	☐	☐	☐	☐	☐
J3	**Ensuring the quality of its end-of-life care**	☐	☐	☐	☐	☐	☐	☐	☐	☐	☐	☐
J4	**Developing a person-centred approach to patients**	☐	☐	☐	☐	☐	☐	☐	☐	☐	☐	☐
J5	**Developing a person-centred approach to staff**	☐	☐	☐	☐	☐	☐	☐	☐	☐	☐	☐
J6	**increasing patient independence and decision-making**	☐	☐	☐	☐	☐	☐	☐	☐	☐	☐	☐
J7	**Making sure that all patients are treated equally**	☐	☐	☐	☐	☐	☐	☐	☐	☐	☐	☐
J8	**Giving staff opportunities to develop their career**	☐	☐	☐	☐	☐	☐	☐	☐	☐	☐	☐
J9	**Supporting staff who provide end-of-life care**	☐	☐	☐	☐	☐	☐	☐	☐	☐	☐	☐
J10	**Making sure that the hospital's beliefs and principles are respected**	☐	☐	☐	☐	☐	☐	☐	☐	☐	☐	☐
J11	**Avoiding legal risks and being open to legal claims**	☐	☐	☐	☐	☐	☐	☐	☐	☐	☐	☐
J12	**Carrying out innovative research**	☐	☐	☐	☐	☐	☐	☐	☐	☐	☐	☐
J13	**Maximising the hospital's financial situation**	☐	☐	☐	☐	☐	☐	☐	☐	☐	☐	☐

J14	**Overall, how would you describe the religious ethos of this hospital?**	Non-religious ☐1	Fairly religious ☐2	Very religious ☐3

Thank you very much for taking the time to complete this questionnaire.

Please remove the front sheet as indicated, put questionnaire in the pre-paid H*f*H envelope, seal the envelope, and return to the Ward Manager (CNM2) who will return it to the designated Hospital Audit Manager for posting to the H*f*H Programme.

Appendix 2

Questionnaire 2

Patient Profile (Doctor Version)

(Yellow)

Questionnaire 2: Patient Profile - Doctor Version

Guide to Instruments

Question	Concept / Domain	Instrument
	Hospital ID	This number will be allocated to each hospital by the H*f*H Programme, and will remain confidential to each hospital.
	Ward ID	This number (from one onwards) will be allocated by the designated audit manager in each hospital, with a separate number allocated to each ward in the audit.
	Patient ID	This number (from one onwards) will be allocated by the designated audit manager in each hospital, with a separate number allocated to each patient in the audit.
**SECTION A: AWARENESS THAT PATIENT WAS DYING**		
A1	Awareness of medical team that patient was dying	Bespoke questions. Same as questions in Section C of Patient Profile Questionnaire (Nurse Version).
A2	Is awareness documented	
A3	Duration of awareness	
A4	Awareness of nursing staff that patient was dying	
A5.1-4	Multidisciplinary team-working	
A6	Could patient have died at home	
**SECTION B: DECISIONS ABOUT TREATMENT**		
B1.1-12	Decisions made about treatment	Bespoke questions. Same as questions as in Section F of Patient Profile Questionnaire (Nurse Version). The question on resuscitation (B1.10) was also used in survey of death in French hospitals[73].
B2.1-6	Experience and management of specific symptoms	
**SECTION C: SPECIALIST PALLIATIVE CARE**<br>Note:		
C1	Is there specialist palliative care service in hospital	Bespoke questions. Same as questions in Section G of Patient Profile Questionnaire (Nurse Version).
C2	Did patient receive specialist palliative care	
C3	Duration of involvement of palliative care	
C4	Benefits of earlier palliative care	
**SECTION D: QUALITY OF CARE**		
D1.1-5	Overall quality of care	Family Evaluation of Hospice Care (FEHC)[74]: Complete Survey, Compressed Version. Six items selected. Same as questions used in Patient Profile Questionnaire (Nurse Version J1-5) and in Survey of Bereaved Relatives, D1-C5.

73 Ferrand, Jabre, Vincent-Genod, et al, 2008.
74 Developed by, and available from, the National Hospice and Palliative Care Organisation (NHPCO), based on Virginia in the US at: http://www.nhpco.org/i4a/pages/Index.cfm?pageid=4397. The Family Evaluation of Hospice Care (FEHC) was developed by Joan Teno and Stephen Connor at Brown University in the US (Connor, Teno, Spence and Smith, 2005) based on a previously validated scale, Toolkit After-Death Bereaved Family Member Interview (Teno, Clarridge, Casey, Edgman-Levitan and Fowler, 2001).

D2.1-2	Acceptability of circumstances in which patient died in hospital	Based on question used in survey of death in French hospitals[75]. Response format changed from 'yes/no' to a 10-point scale. Same question –focused on individual patients - used in Patient Profile Questionnaire (Nurse Version J8.1-2) and Survey of Bereaved Relatives (D6-7). Same question –focused on ward - used in Patient Profile Questionnaire (Nurse Version E17.1-2) and Survey of Ward Perceptions (E17.1-2). Same question –focused on hospital - used in Survey of Hospital Perceptions (D1.1-2).
**SECTION E: REVIEW OF PATIENT'S DYING AND DEATH**		
E5.1	What went well?	Open-ended question. Same question is used in Patient Profile Questionnaire (Nurse Version M6.1), and in Survey of Bereaved Relatives, F1.
E5.2	What did not go so well?	Open-ended question. Same question is used in Patient Profile Questionnaire (Nurse Version M6.2), and in Survey of Bereaved Relatives, F2.
E5.3	What could have been better?	Open-ended question. Same question is used in Patient Profile Questionnaire (Nurse Version M6.3), and in Survey of Bereaved Relatives, F3.
**SECTION F: BACKGROUND INFORMATION OF DOCTOR**		
F1	Position within the hospital	Bespoke question. Same question as used in Patient Profile Questionnaire (Nurse Version, Section A), Ward Perceptions Questionnaire (Section A), and Hospital Perceptions Questionnaire (Section A).
F2	Length of time working in hospital	
F3	Country where brought up	Categories taken from Yeates[76] which summarises the country of origin of nurses in Ireland. Same question as used in Patient Profile Questionnaire (Nurse Version, Section A), Ward Perceptions Questionnaire (Section A), and Hospital Perceptions Questionnaire (Section A).
F4	Gender of respondent	Bespoke question. Same question as used in Patient Profile Questionnaire (Nurse Version, Section A), Ward Perceptions Questionnaire (Section A), and Hospital Perceptions Questionnaire (Section A).
F5	Age of respondent	
F6	Is English your first language?	

75 Ferrand, Jabre, Vincent-Genod, et al, 2008.
76 Yeates, 2006:15

Questionnaire 2
Patient profile - Doctor version

Thank you for taking the time to complete this questionnaire. The questionnaire is part of a hospital audit of its end-of-life care. Its main focus is on the services offered to patients in their final week of life. This audit has been ethically approved, and is fully endorsed by the hospital's management team. The audit was developed by the Hospice Friendly Hospitals (H*f*H) Programme, an initiative of the Irish Hospice Foundation. It is part of a national audit of end-of-life care in acute and community hospitals.

The questionnaire should be completed by the consultant doctor who primarily looked after the patient, or by a hospital doctor designated by the consultant, such as a specialist registrar or registrar. The questionnaire asks for information about the hospital services the patient got during their last week of life.

The H*f*H Programme will analyse the information in this audit and they will write a confidential report for the hospital. They will not use any names or other signifiers in any published reports which could link this information to any particular hospital, to any staff, to any patients, or to their relatives. The value of the audit depends on the quality of the information you give in this questionnaire. So, it is important that you carefully fill out the questionnaire with accurate and honest answers to each question.

Your answers will stay completely confidential. You should not write your name on the questionnaire.

You should complete the questionnaire as soon as possible after the patient has died, preferably within one week of their death.

Before beginning to answer the questionnaire, read these instructions:

- ✓ Check the bottom of this page to make sure it has this information: Hospital ID, Ward ID, and Patient ID (for the audit, the patient's ID is different from patient's hospital number). If not, contact the Ward Manager (CNM2) immediately. **Do not start unless the questionnaire has this information.**
- ✓ You may need to have the patient's hospital chart to complete some of the questions.
- ✓ Try not to answer 'Don't know', unless you really need to.
- ✓ Make sure you tick the boxes clearly, and write clearly in the text boxes (especially E5.1 to E5.3).
- ✓ Check that you have answered every question before putting the questionnaire in the envelope provided.
- ✓ Put the completed questionnaire in the stamped-addressed envelope provided, seal it, and return to the Ward Manager (CNM2) of the ward where the patient died. They will pass it on to the designated Hospital Audit Manager who will post it to the H*f*H office in Dublin.

Thank you for contributing to your hospital's audit of end-of-life care by completing this questionnaire.

Hospital ID	Ward ID	Patient ID

Section A: Awareness that the patient was dying

A1	**Had the medical team diagnosed that this patient was dying?**	☐1 Yes	☐0 No

A2	**If yes, was this documented in the patient's hospital chart?**	☐1 Yes	☐0 No

A3	**If yes, how long before the patient's death were the medical team aware that the patient was dying?** (If less than a day, write the number of hours)	Weeks	Days	Hours

A4	**Were the nursing staff aware that the patient was dying?**	☐1 Yes	☐0 No	☐9 Don't know

**If the staff were aware that the patient was dying, did any of these things happen?**				
A5.1	**The multidisciplinary team (all health care professionals involved in the care of the patient) had a meeting to talk about and review the aims of the patient's care**	☐1 Yes	☐0 No	☐9 Don't know
A5.2	**The medical and nursing staff involved in the care of this patient had a meeting to talk about and review the aims of the patient's care**	☐1 Yes	☐0 No	☐9 Don't know
A5.3	**The patient or family were involved in the meeting to talk about and review the aims of the patient's care**	☐1 Yes	☐0 No	☐9 Don't know
A5.4	**The patient or family were told later about the result of the meeting to talk about and review the aims of the patient's care**	☐1 Yes	☐0 No	☐9 Don't know

A6	**Depending on the patient's condition during their last week of life, do you think the patient could have been allowed to die at home, if they got enough home care support?**	☐1 Yes	☐0 No	☐9 Don't know

Section B: Decisions about treatment

**At any time during the patient's last week of life, was a decision made to do any of these things?**		**Was a decision made?**			**Was the decision documented?**		
		Yes	No	Doesn't apply*	Yes	No	Doesn't apply*
B1.1	**Review whether the aim of patient's care was curative or palliative**	☐1	☐0	☐9	☐1	☐0	☐9
B1.2	**Change medical treatments to optimise patient's comfort and manage symptoms**	☐1	☐0	☐9	☐1	☐0	☐9
B1.3	**Review medication, route of administration, and stop non-essential medication**	☐1	☐0	☐9	☐1	☐0	☐9
B1.4	**Stop blood tests that have no connection to the patient's treatment or care**	☐1	☐0	☐9	☐1	☐0	☐9
B1.5	**Stop antibiotics if they are not adding to the patient's comfort or supportive care**	☐1	☐0	☐9	☐1	☐0	☐9
B1.6	**Review artificial hydration (IV or SC fluids) in terms of benefit versus burden**	☐1	☐0	☐9	☐1	☐0	☐9

At any time during the patient's last week of life, was a decision made to do any of these things?		Was a decision made?			Was the decision documented?		
		Yes	No	Doesn't apply*	Yes	No	Doesn't apply*
B1.7	**Stop invasive monitoring**	☐1	☐0	☐9	☐1	☐0	☐9
B1.8	**Hold back treatment, for example a decision not to start or not to increase a life-sustaining intervention**	☐1	☐0	☐9	☐1	☐0	☐9
B1.9	**Stop giving treatment, for example a decision to stop or to decrease a life-sustaining intervention**	☐1	☐0	☐9	☐1	☐0	☐9
B1.10	**Discuss and record resuscitation status with patient or relative**	☐1	☐0	☐9	☐1	☐0	☐9

*This question does not apply if the patient's death was sudden or unexpected.

During their last week of life, patients sometimes have these symptoms. For each symptom, how often did the patient have this in their last week of life?						If the patient had this symptom at any time during their last week of life, how well it was managed by the hospital team to keep the patient comfortable? For each symptom, rate how well it was managed on this scale. If the patient did not have this symptom, tick the NA (Not Applicable) box.										
		All the time	Most of the time	Some of the time	None of the time	Very badly ⬇ 1	2	3	4	5	6	7	8	9	Excellent ⬇ 10	NA 99
B2.1	**Pain**	☐1	☐2	☐3	☐4	☐	☐	☐	☐	☐	☐	☐	☐	☐	☐	☐
B2.2	**Nausea and / or vomiting**	☐1	☐2	☐3	☐4	☐	☐	☐	☐	☐	☐	☐	☐	☐	☐	☐
B2.3	**Breathing difficulties**	☐1	☐2	☐3	☐4	☐	☐	☐	☐	☐	☐	☐	☐	☐	☐	☐
B2.4	**Increased secretions**	☐1	☐2	☐3	☐4	☐	☐	☐	☐	☐	☐	☐	☐	☐	☐	☐
B2.5	**Restlessness or agitation**	☐1	☐2	☐3	☐4	☐	☐	☐	☐	☐	☐	☐	☐	☐	☐	☐
B2.6	**Anxiety or fear**	☐1	☐2	☐3	☐4	☐	☐	☐	☐	☐	☐	☐	☐	☐	☐	☐

Section C: Specialist palliative care

C1	**Is there a specialist palliative care service in this hospital?**	☐1 Yes	☐0 No	☐9 Don't know

If there is no palliative care service, go to F5.

C2	**Did the patient get any contribution from a specialist palliative care service after they were admitted to hospital?**	☐1 Yes	☐0 No	☐9 Don't know

		Weeks	Days	Hours
C3	**If yes, how long before death did the patient get this contribution from a specialist palliative care service?** (If less than a day, write the number of hours)			

C4	If the patient did not get any contribution from a specialist palliative care service, or if there is no such team in the hospital, do you think he or she would have benefited from this?	☐1 Yes	☐0 No	☐9 Don't know

Section D: Quality of care

D1	How well do you think the hospital team communicated with the patient about his or her situation and their likely prognosis?	Not well ↓									Very well ↓	Don't know
		☐1	☐2	☐3	☐4	☐5	☐6	☐7	☐8	☐9	☐10	☐99

D2	How well do you think the hospital team communicated with the relatives or friends about the patient's illness and the likely prognosis?	Not well ↓									Very well ↓	Don't know
		☐1	☐2	☐3	☐4	☐5	☐6	☐7	☐8	☐9	☐10	☐99

D3	How well do you think the hospital team provided end-of-life care that respected the patient's wishes?	Not well ↓									Very well ↓	Don't know
		☐1	☐2	☐3	☐4	☐5	☐6	☐7	☐8	☐9	☐10	☐99

D4	How well do you think the hospital team managed the patient's symptoms, such as pain, to a level that was acceptable to him or her?	Not well ↓									Very well ↓	Don't know
		☐1	☐2	☐3	☐4	☐5	☐6	☐7	☐8	☐9	☐10	☐99

D5	How well do you think the hospital team provided emotional support for the family or friends of the patient?	Not well ↓									Very well ↓	Don't know
		☐1	☐2	☐3	☐4	☐5	☐6	☐7	☐8	☐9	☐10	☐99

Do you feel the way this patient died in hospital would be acceptable for you, or for your family or friends?		Definitely not acceptable ↓									Very acceptable ↓	Don't know
		1	2	3	4	5	6	7	8	9	10	0
D6.1	Acceptable for you?	☐	☐	☐	☐	☐	☐	☐	☐	☐	☐	☐
D6.2	Acceptable for your family or friends?	☐	☐	☐	☐	☐	☐	☐	☐	☐	☐	☐

Section E: Review of patient's dying and death

E5.1 **What went well in the care of this patient during their last week of life?**

____

____

____

E5.2 **What did not go well in the care of this patient during their last week of life?**

____

____

____

E5.3 **What could have been done better in the care of this patient during their last week of life?**

____

____

____

Section F: Background characteristics of doctor

F1	**What is your position in the hospital?**	☐1 Consultant doctor	☐2 Non-consultant doctor

F2	**How long have you been working in this hospital?**	(to the nearest year)

F3	**Where were you brought up?**	Australia	India	Ireland	Middle East	Nigeria	New Zealand
		☐1	☐2	☐3	☐4	☐5	☐6
		Pakistan	Philippines	South Africa	UK	US	Other
		☐7	☐8	☐9	☐10	☐11	☐12

F4	**Are you male or female?**	☐1 Male	☐2 Female

F5	**How old are you?**	(to the nearest year)

F6	**Is English your first language?**	☐1 Yes	☐2 No

Thank you very much for taking the time to complete this questionnaire.

Please remove the identifying front sheet as indicated, put the questionnaire in the pre-paid H*f*H envelope, seal the envelope, and return to the Ward Manager (CNM2) who will return it to the designated Hospital Audit Manager who will post it to the H*f*H Programme.

Appendix 3

Questionnaire 3

Bereaved Relatives

(Blue)

Questionnaire 3:
Survey of Bereaved Relatives & Friends

Guide to Instruments

Question	Concept / Domain	Instrument
	Hospital ID	This number will be allocated to each hospital by the H*f*H Programme, and will remain confidential to each hospital.
	Ward ID	This number (from one onwards) will be allocated by the audit manager in each hospital, with a separate number allocated to each ward in the audit.
	Patient ID	This number (from one onwards) will be allocated by the audit manager in each hospital, with a separate number allocated to each patient in the audit.
**SECTION A: BACKGROUND INFORMATION**		
A1	Relationship to relative	Response categories taken from Family Evaluation of Hospice Care (FEHC)[77] and Quality of Dying and Death Instrument (QODD)[78].
A2-A3	Gender and Age	Standard questions
A4	Carer of relative / friend	Bespoke question
**SECTION B: EXPERIENCE OF HOSPITAL AND WARD**		
B1-B2	Type of room	Bespoke questions. Same questions are used in Patient Profile Questionnaire (Nurse Version B4.1-2) and Ward Perceptions Questionnaire (C7.1-16).
B3-B4	Preferences for single room	Bespoke questions.
B5.1-16	Assessment of physical space where patient spent most of the time during last week	Bespoke questions. Same questions are used in Patient Profile Questionnaire (Nurse Version B7.1-16).
B6.1-10	Assessment of hospital facilities	Bespoke questions.
B7.1-2	Adequacy of number and quality of medical staff	Bespoke questions.
B8.1-2	Adequacy of number and quality of nursing staff	Bespoke questions.
B9.1-2	Adequacy of number and quality of other staff	Bespoke questions.
B10	Was ward well organised?	Bespoke questions.
B11	How well did staff respond to requests	Bespoke questions.
B12	Rating of end-of-life care in ward	Bespoke questions.
**SECTION C: QUALITY OF LIFE IN THE LAST WEEK**		
C1-B25	Relative's assessment of patient's experiences during last week of life	QODD: Significant Other After Death Interview / Seven Day Version. All but six items included. Same question is used in the Patient Profile Questionnaire (Nurse Version H1-J25).

77 Developed by, and available from, the National Hospice and Palliative Care Organisation (NHPCO), based on Virginia in the US at: http://www.nhpco.org/i4a/pages/Index.cfm?pageid=4397. The Family Evaluation of Hospice Care (FEHC) was developed by Joan Teno and Stephen Connor at Brown University in the US (Connor, Teno, Spence and Smith, 2005) based on a previously validated scale, Toolkit After-Death Bereaved Family Member Interview (Teno, Clarridge, Casey, Edgman-Levitan and Fowler, 2001).

78 Developed by, and available from, the University of Washington End of Life Care Research Program at: http://depts.washington.edu/eolcare/instruments/index.html. The Quality of Dying and Death Instrument (QODD) was developed by Donald Patrick, Ruth Engleberg and Randall Curtis (Patrick, Engleberg and Curtis 2001) and has been validated in three studies (Curtis, Patrick, Engleberg, Norris, Asp, and Byock, 2002; Hodde, Engelberg, Treece, Steinberg, and Curtis, 2004; Mularski, Heine, Osborne, Ganzini, and Curtis, 2005).

C26	Could patient have died at home	Bespoke question. Same question is used in Patient Profile Questionnaire (Nurse Version, C6 and Doctor Version A6),.
C27	Did patient wish to die at home	Bespoke question.
C28	Did relative wish patient to be cared for at home	Bespoke questions
**SECTION D: QUALITY OF CARE**		
D1-C5	Overall quality of care	FEHC: Complete Survey, Compressed Version. Six items selected. Same question is used in Patient Profile Questionnaire (Nurse Version J1-J5).
D6.1-2	Acceptability of circumstances in which patient died in hospital	Based on question used in survey of death in French hospitals[79]. Response format changed from 'yes/no' to a 10-point scale. Same question – focused on individual patients - used in Patient Profile Questionnaire (Nurse Version J8.1-2) , Patient Profile Questionnaire (Doctor Version D6.1-2) and Survey of Bereaved Relatives (C6.1-2). Same question –focused on ward - used in Patient Profile Questionnaire (Nurse Version E17.1-2) and Survey of Ward Perceptions (E17.1-2). Same question –focused on hospital - used in Survey of Hospital Perceptions (D1.1-2).
**SECTION E: POST MORTEM**		
E1	Was a post mortem held	Bespoke questions, but informed by the Report of Dr. Deirdre Madden on Post Mortem Practice and Procedures (January 2006)[80], and the Report of the Working Group on Post Mortem Practice (November 2006)[81].
E2	Was it requested by hospital or coroner	
E3	Was information offered	
E4.1-3	Was information satisfactory	
**SECTION F: COMMENTS**		
F1	What went well?	Open-ended questions. Same question is used in Patient Profile Questionnaire (Nurse Version M5.1 and Doctor Version E5.1).
F2	What did not go so well?	Open-ended questions. Same question is used in Patient Profile Questionnaire (Nurse Version M5.2 and Doctor Version E5.2).
F3	What could have been done better?	Open-ended questions. Same question is used Patient Profile Questionnaire (Nurse Version M5.3 and Doctor Version E5.3).
**SECTION G: FINAL THOUGHTS**		
G1	Quality of end-of-life care in Irish hospitals	Based on question used in 2004 nationwide survey of public attitudes and experiences regarding death and dying[82].
G2.1-8	The three most important things about end-of-life care	Based on question used in 2004 nationwide survey of public attitudes and experiences regarding death and dying[83].

79 Ferrand, Jabre, Vincent-Genod, et al, 2008.
80 Madden, 2006
81 Working Group on Post Mortem Practice, 2006
82 Weafer and Associates Research, 2004:16-17.
83 Weafer and Associates Research, 2004:16-17.

Questionnaire 3
Survey of bereaved relatives and friends

Thank you for taking the time to fill out this survey. We are doing this survey so that the hospital where your relative or friend was cared for can review and improve its services for patients, especially for patients who spend their last days in hospital. We value your views on your relative's or friend's experiences in hospital, particularly during their last week of life.

We will carefully analyse your views on the services which the hospital offered your relative or friend, and we will use this information to improve these services. **All of the information you put in this questionnaire is absolutely confidential. We will never tell anyone who you are or who your relative or friend is.**

We would like you to answer every question. However, if you find that some of the questions bring back some upsetting memories, please feel free to skip them and move on. We have enclosed a list of bereavement support services and their contact details, if you need to use them.

Guidelines for filling out the questionnaire:

1) The questionnaire should be filled out by the relative or friend who knows most about the hospital care the person got during their last week of life. You might want to talk to other family members to help you answer some of the questions.
2) Please answer each question by ticking one box that best describes your experience and the experience of your relative or friend during their last stay in hospital. You can ignore the numbers beside the boxes, we use these to help us analyse your answers.
3) At the end of the questionnaire there is space for you to add comments about the care your relative or friend got while they were in hospital. Please write your comments clearly.
4) When you have finished the questionnaire, please put it in the pre-paid Hospice friendly Hospitals envelope which we've enclosed and put it in the post. You do not need a stamp.

☐ **By filling out the questionnaire and ticking this box you are indicating that you have read our letters about the study and are agreeing to participate voluntarily.**

Thank you very much for completing the questionnaire.

For administrative use only:

Hospital ID	Ward ID	Patient ID

Please answer by ticking one box for each question.

Section A: Background information

A1	What is your relationship to the person who died in hospital?	I am his or her …							
		husband or wife ☐1	partner ☐2	child ☐3	parent ☐4	brother or sister ☐5	other relative ☐6	friend ☐7	other ☐8

A2	Are you male or female?	Male ☐1	Female ☐2

A3	How old are you?	years

A4	In the time before your relative's or friend's final admission to hospital, did you provide care for this person?	Yes ☐1	No ☐0

Section B: Experience of the hospital and ward

Type of room

B1	In what type of room, did your relative or friend die?	Single room ☐1	Shared room ☐2

B2	If your relative or friend died in a shared room, would they have preferred to die in a single room?	Yes ☐1	No ☐0	Don't know ☐9

B3	If your relative or friend died in a shared room, would you have preferred if they had died in a single room?	Yes ☐1	No ☐0	Don't know ☐9

B4	Where did your relative or friend spend most of the time during their last week of life?	Single room ☐1	Shared room ☐2

Please rate the room where your relative or friend spent most of their time during the last week of their life.		Very poor ↓ 1	2	3	4	5	6	7	8	9	Excellent ↓ 10
B5.1	Patients and family members could have private conversations with hospital staff	☐	☐	☐	☐	☐	☐	☐	☐	☐	☐
B5.2	Patients could have private conversations with family members	☐	☐	☐	☐	☐	☐	☐	☐	☐	☐
B5.3	Family members could stay as long as they want	☐	☐	☐	☐	☐	☐	☐	☐	☐	☐
B5.4	Patients could have dignity during their personal care, like baths.	☐	☐	☐	☐	☐	☐	☐	☐	☐	☐
B5.5	The patient had easy access to a toilet and shower or bath	☐	☐	☐	☐	☐	☐	☐	☐	☐	☐
B5.6	Patients could choose company or to be alone	☐	☐	☐	☐	☐	☐	☐	☐	☐	☐

Please rate the room where your relative or friend spent most of their time during the last week of their life.		Very poor 1	2	3	4	5	6	7	8	9	Excellent 10
B5.7	Patients could see nature	☐	☐	☐	☐	☐	☐	☐	☐	☐	☐
B5.8	Patients could see natural daylight	☐	☐	☐	☐	☐	☐	☐	☐	☐	☐
B5.9	Patients could experience quiet	☐	☐	☐	☐	☐	☐	☐	☐	☐	☐
B5.10	Patients could listen to TV / radio without disturbing others	☐	☐	☐	☐	☐	☐	☐	☐	☐	☐
B5.11	Patients could personalise the space	☐	☐	☐	☐	☐	☐	☐	☐	☐	☐
B5.12	Patients could turn on or off the TV or radio	☐	☐	☐	☐	☐	☐	☐	☐	☐	☐
B5.13	Patients could control the room temperature	☐	☐	☐	☐	☐	☐	☐	☐	☐	☐
B5.14	Patients could control the light in the room	☐	☐	☐	☐	☐	☐	☐	☐	☐	☐
B5.15	Patients could control the air in the room	☐	☐	☐	☐	☐	☐	☐	☐	☐	☐

Hospital facilities

Please rate your experience of the hospital's facilities where your relative or friend died.		Poor	Fair	Good	Very good	Excellent	Not available
B6.1	Car parking	☐1	☐2	☐3	☐4	☐5	☐6
B6.2	Finding your way around the hospital	☐1	☐2	☐3	☐4	☐5	☐6
B6.3	A meeting room for private conversations with staff	☐1	☐2	☐3	☐4	☐5	☐6
B6.4	A quiet sitting room for patients and relatives	☐1	☐2	☐3	☐4	☐5	☐6
B6.5	A child-friendly TV lounge	☐1	☐2	☐3	☐4	☐5	☐6
B6.6	A relative's room with shower and toilet	☐1	☐2	☐3	☐4	☐5	☐6
B6.7	A storage facility for patients' belongings	☐1	☐2	☐3	☐4	☐5	☐6
B6.8	A multi-faith space	☐1	☐2	☐3	☐4	☐5	☐6
B6.9	Being able to get hot and cold drinks, food etc.	☐1	☐2	☐3	☐4	☐5	☐6
B6.10	Access to toilets	☐1	☐2	☐3	☐4	☐5	☐6

What do you think about the hospital staff?

B7	During the last week of life of your relative or friend, how would you rate the way the staff responded to your requests?	Very poor ☐1	Poor ☐2	Average ☐3	Good ☐4	Very good ☐5	Don't know ☐9

Medical staff

B8.1	Were there enough medical staff on the ward where your relative or friend died?	Definitely not enough ☐1	Not enough ☐2	Enough ☐3	Definitely enough ☐4	Don't know ☐9

B8.2	What did you think of the quality of the medical staff on the ward where your relative or friend died?	Very poor ☐1	Poor ☐2	Average ☐3	Good ☐4	Don't know ☐9

Nursing staff

B9.1	**Were there enough nursing staff on the ward where your relative or friend died?**	Definitely not enough ☐1	Not enough ☐2	Enough ☐3	Definitely enough ☐4	Don't know ☐9

B9.2	**What did you think of the quality of nursing staff on the ward where your relative or friend died?**	Very poor ☐1	Poor ☐2	Average ☐3	Good ☐4	Don't know ☐9

B9.3	**Did you have any problems getting nursing staff on the ward to understand you?**	A lot of problems ☐1	Some problems ☐2	No problems ☐3	Don't know ☐9

Other hospital staff

B10.1	**Were there enough other staff on the ward where your relative or friend died?**	Definitely not enough ☐1	Not enough ☐2	Enough ☐3	Definitely enough ☐4	Don't know ☐9

B10.2	**What did you think about the quality of other staff in this ward?**	Very poor ☐1	Poor ☐2	Average ☐3	Good ☐4	Don't know ☐9

Ward

B11	**Did you think the ward was well-organised?**	Very disorganised ☐1	Fairly disorganised ☐2	Relatively organised ☐3	Very well organised ☐4	Don't know ☐9

B12	**Overall, how would you rate this ward as a place for someone to get care at the end of their life?**	Very poor ☐1	Poor ☐2	Average ☐3	Good ☐4	Very good ☐5	Don't know ☐9

Section C: Quality of life in the person's last week

The following questions are about your relative's or friend's experience during the last week of their life.
Please answer each question, even if they were unconscious for some or all of the time.
If you don't know how to answer, please tick the box marked "Don't know".

C1a	**Did your relative or friend have physical pain?**	None of the time ☐1	A little bit of the time ☐2	Some of the time ☐3	A good bit of the time ☐4	Most of the time ☐5	All of the time ☐6	Don't know ☐9

C1b	**How would you rate this part of your relative's or friend's experience?**	Unsatisfactory ☐1	☐2	☐3	☐4	☐5	☐6	☐7	☐8	☐9	Satisfactory ☐10	Don't know ☐99

C2a	**Was your relative or friend able to eat or drink?**	None of the time ☐1	A little bit of the time ☐2	Some of the time ☐3	A good bit of the time ☐4	Most of the time ☐5	All of the time ☐6	Don't know ☐9

C2b	How would you rate this part of your relative's or friend's experience?	Unsatisfactory ☐1	☐2	☐3	☐4	☐5	☐6	☐7	☐8	☐9	Satisfactory ☐10	Don't know ☐99

C3a	Did your relative or friend have breathing problems?	None of the time ☐1	A little bit of the time ☐2	Some of the time ☐3	A good bit of the time ☐4	Most of the time ☐5	All of the time ☐6	Don't know ☐9

C3b	How would you rate this part of your relative's or friend's experience?	Unsatisfactory ☐1	☐2	☐3	☐4	☐5	☐6	☐7	☐8	☐9	Satisfactory ☐10	Don't know ☐99

C4a	Did your relative or friend seem comfortable and at ease?	None of the time ☐1	A little bit of the time ☐2	Some of the time ☐3	A good bit of the time ☐4	Most of the time ☐5	All of the time ☐6	Don't know ☐9

C4b	How would you rate this part of your relative's or friend's experience?	Unsatisfactory ☐1	☐2	☐3	☐4	☐5	☐6	☐7	☐8	☐9	Satisfactory ☐10	Don't know ☐99

C5a	Did your relative or friend seem afraid or anxious?	None of the time ☐1	A little bit of the time ☐2	Some of the time ☐3	A good bit of the time ☐4	Most of the time ☐5	All of the time ☐6	Don't know ☐9

C5b	How would you rate this part of your relative's or friend's experience?	Unsatisfactory ☐1	☐2	☐3	☐4	☐5	☐6	☐7	☐8	☐9	Satisfactory ☐10	Don't know ☐99

C6a	Did your relative or friend smile, laugh or show any signs of enjoyment in their last week?	None of the time ☐1	A little bit of the time ☐2	Some of the time ☐3	A good bit of the time ☐4	Most of the time ☐5	All of the time ☐6	Don't know ☐9

C6b	How would you rate this part of your relative's or friend's experience?	Unsatisfactory ☐1	☐2	☐3	☐4	☐5	☐6	☐7	☐8	☐9	Satisfactory ☐10	Don't know ☐99

C7a	Did your relative or friend seem to have the energy to do most of the things that they wanted to do?	None of the time ☐1	A little bit of the time ☐2	Some of the time ☐3	A good bit of the time ☐4	Most of the time ☐5	All of the time ☐6	Don't know ☐9

C7b	How would you rate this part of your relative's or friend's experience?	Unsatisfactory ☐1	☐2	☐3	☐4	☐5	☐6	☐7	☐8	☐9	Satisfactory ☐10	Don't know ☐99

C8a	Was your relative or friend able to physically control when they went to the toilet?	None of the time ☐1	A little bit of the time ☐2	Some of the time ☐3	A good bit of the time ☐4	Most of the time ☐5	All of the time ☐6	Don't know ☐9

C8b	How would you rate this part of your relative's or friend's experience?	Unsatisfactory ☐1	☐2	☐3	☐4	☐5	☐6	☐7	☐8	☐9	Satisfactory ☐10	Don't know ☐99

C9a	Was your relative's or friend's dignity and self-respect maintained?	None of the time ☐1	A little bit of the time ☐2	Some of the time ☐3	A good bit of the time ☐4	Most of the time ☐5	All of the time ☐6	Don't know ☐9

C9b	How would you rate this part of your relative's or friend's experience?	Unsatisfactory ☐1	☐2	☐3	☐4	☐5	☐6	☐7	☐8	☐9	Satisfactory ☐10	Don't know ☐99

Spending time with family and friends

If the patient had a living husband, wife or partner:

C10a	Was your relative or friend helped to spend time with their husband, wife or partner in the way they wanted to?	None of the time ☐1	A little bit of the time ☐2	Some of the time ☐3	A good bit of the time ☐4	Most of the time ☐5	All of the time ☐6	Don't know ☐9

C10b	How would you rate this part of your relative's or friend's experience?	Unsatisfactory ☐1	☐2	☐3	☐4	☐5	☐6	☐7	☐8	☐9	Satisfactory ☐10	Don't know ☐99

If the patient had children:

C11a	Was your relative or friend helped to spend time with their children in the way they wanted to?	None of the time ☐1	A little bit of the time ☐2	Some of the time ☐3	A good bit of the time ☐4	Most of the time ☐5	All of the time ☐6	Don't know ☐9

C11b	How would you rate this part of your relative's or friend's experience?	Unsatisfactory ☐1	☐2	☐3	☐4	☐5	☐6	☐7	☐8	☐9	Satisfactory ☐10	Don't know ☐99

C12a	Was your relative or friend helped to spend time with family members and friends in the way they wanted to?	None of the time ☐1	A little bit of the time ☐2	Some of the time ☐3	A good bit of the time ☐4	Most of the time ☐5	All of the time ☐6	Don't know ☐9

C12b	How would you rate this part of your relative's or friend's experience?	Unsatisfactory ☐1	☐2	☐3	☐4	☐5	☐6	☐7	☐8	☐9	Satisfactory ☐10	Don't know ☐99

C13a	Was your relative or friend helped to spend time alone in the way they wanted to?	None of the time ☐1	A little bit of the time ☐2	Some of the time ☐3	A good bit of the time ☐4	Most of the time ☐5	All of the time ☐6	Don't know ☐9

C13b	How would you rate this part of your relative's or friend's experience?	Unsatisfactory ☐1	☐2	☐3	☐4	☐5	☐6	☐7	☐8	☐9	Satisfactory ☐10	Don't know ☐99

C14a	Did your relative or friend seem worried about causing strain to his or her loved ones?	None of the time ☐1	A little bit of the time ☐2	Some of the time ☐3	A good bit of the time ☐4	Most of the time ☐5	All of the time ☐6	Don't know ☐9

C14b	How would you rate this part of your relative's or friend's experience?	Unsatisfactory ☐1	☐2	☐3	☐4	☐5	☐6	☐7	☐8	☐9	Satisfactory ☐10	Don't know ☐99

C15a	Did your relative or friend have his or her loved ones around them in the last week of their life?	Yes ☐1	No ☐0	Don't know ☐9

C15b	How would you rate this part of your relative's or friend's experience?	Unsatisfactory ☐1	☐2	☐3	☐4	☐5	☐6	☐7	☐8	☐9	Satisfactory ☐10	Don't know ☐99

C16a	Did your relative or friend say goodbye to their loved ones?	Yes ☐1	No ☐0	Don't know ☐9

C16b	How would you rate this part of your relative's or friend's experience?	Unsatisfactory ☐1	☐2	☐3	☐4	☐5	☐6	☐7	☐8	☐9	Satisfactory ☐10	Don't know ☐99

Other questions

C17a	Did your relative or friend seem to have meaning and purpose in the last week of their life?	Yes ☐1	No ☐0	Don't know ☐9

C17b	How would you rate this part of your relative's or friend's experience?	Unsatisfactory ☐1	☐2	☐3	☐4	☐5	☐6	☐7	☐8	☐9	Satisfactory ☐10	Don't know ☐99

C18a	Did your relative or friend have any money worries, such as the cost of health care?	Yes ☐1	No ☐0	Don't know ☐9

C18b	How would you rate this part of your relative's or friend's experience?	Unsatisfactory ☐1	☐2	☐3	☐4	☐5	☐6	☐7	☐8	☐9	Satisfactory ☐10	Don't know ☐99

C19a	Did your relative or friend have one or more visits from a religious or spiritual advisor, such as a priest?	Yes ☐1	No ☐0	Don't know ☐9

C19b	How would you rate this part of your relative's or friend's experience?	Unsatisfactory ☐1	☐2	☐3	☐4	☐5	☐6	☐7	☐8	☐9	Satisfactory ☐10	Don't know ☐99

C20a	Did your relative or friend have a religious or spiritual service or ceremony before their death?	Yes ☐1	No ☐0	Don't know ☐9

C20b	How would you rate this part of your relative's or friend's experience?	Unsatisfactory ☐1	☐2	☐3	☐4	☐5	☐6	☐7	☐8	☐9	Satisfactory ☐10	Don't know ☐99

Moment of death

C21a	**Was anyone there at the moment of your relative's or friend's death?**	Yes ☐1	No ☐0	Don't know ☐9

C21b	**How would you rate this part of your relative's or friend's experience?**	Unsatisfactory ↓ ☐1	☐2	☐3	☐4	☐5	☐6	☐7	☐8	☐9	Satisfactory ↓ ☐10	Don't know ☐99

C22a	**In the moment before your relative or friend died, was he or she ...**	alert ❑1	semi-conscious but able to speak ❑2	unconscious ❑3	Don't know ❑9

C22b	**How would you rate this part of your relative's or friend's experience?**	Unsatisfactory ↓ ☐1	☐2	☐3	☐4	☐5	☐6	☐7	☐8	☐9	Satisfactory ↓ ☐10	Don't know ☐99

C23	**Overall, how would you rate the quality of the moment of your relative's or friend's death?**	Unsatisfactory ↓ ☐1	☐2	☐3	☐4	☐5	☐6	☐7	☐8	☐9	Satisfactory ↓ ☐10	Don't know ☐99

Last week of life

C24	**Overall, how would you rate your relative's or friend's quality of life during their last week?**	Unsatisfactory ↓ ☐1	☐2	☐3	☐4	☐5	☐6	☐7	☐8	☐9	Satisfactory ↓ ☐10	Don't know ☐99

C25	**Overall, how would you rate your relative's or friend's quality of dying during their last week?**	Unsatisfactory ↓ ☐1	☐2	☐3	☐4	☐5	☐6	☐7	☐8	☐9	Satisfactory ↓ ☐10	Don't know ☐99

C26	**Depending on your relative's or friend's condition during their last week of life, do you think they could have been allowed to die at home if there was enough support?**	Yes ☐1	No ☐0	Don't know ☐9

C27	**During their last week of life, did your relative or friend say that they would like to die at home?**	Yes ☐1	No ☐0	Don't know ☐9

C28	**During their last week of life, would you have liked your relative or friend to be cared for at home?**	Yes ☐1	No ☐0	Don't know ☐9

Section D: Quality of care

D1	**How well did the hospital team communicate with your relative or friend about his or her illness and how it would develop?**	Poor ☐1	☐2 ☐3 ☐4 ☐5 ☐6 ☐7 ☐8 ☐9	Excellent ☐10	Don't know ☐99

D1	**How well did the hospital team communicate with your relative or friend about his or her illness and how it would develop?**	Poor ☐1	☐2 ☐3 ☐4 ☐5 ☐6 ☐7 ☐8 ☐9	Excellent ☐10	Don't know ☐99
D2	**How well did the hospital team communicate with the family about your relative's or friend's illness and how it would develop?**	Poor ☐1	☐2 ☐3 ☐4 ☐5 ☐6 ☐7 ☐8 ☐9	Excellent ☐10	Don't know ☐99
D3	**How well did the hospital team provide end-of-life care that respected your relative's or friend's wishes?**	Poor ☐1	☐2 ☐3 ☐4 ☐5 ☐6 ☐7 ☐8 ☐9	Excellent ☐10	Don't know ☐99
D4	**How well did the hospital team manage your relative's or friend's symptoms (such as pain) that was acceptable to him or her?**	Poor ☐1	☐2 ☐3 ☐4 ☐5 ☐6 ☐7 ☐8 ☐9	Excellent ☐10	Don't know ☐99
D5	**How well do you think the hospital team provided emotional support for you and the family of your relative or friend?**	Poor ☐1	☐2 ☐3 ☐4 ☐5 ☐6 ☐7 ☐8 ☐9	Excellent ☐10	Don't know ☐99
D6	**Do you feel the way your relative or friend died in hospital would be acceptable for you?**	Definitely not acceptable ☐1	☐2 ☐3 ☐4 ☐5 ☐6 ☐7 ☐8 ☐9	Very acceptable ☐10	Don't know ☐99
D7	**Do you feel the way your relative or friend died in hospital would be acceptable for your family and friends?**	Definitely not acceptable ☐1	☐2 ☐3 ☐4 ☐5 ☐6 ☐7 ☐8 ☐9	Very acceptable ☐10	Don't know ☐99

Section E: Post mortem

A post mortem is an examination of the person after they have died. It usually happens when the hospital is not sure why the person has died and they need to find out more information about this.

E1	**Was there a post mortem after the death of your relative or friend?**	Yes ☐1	No ☐0

If there was no post mortem, please go to the Section E.

E2	**If yes, do you know if the hospital or coroner asked for a post mortem?**	Asked by hospital ☐1	Asked by coroner ☐2	Don't know ☐9
E3	**If there was a post mortem after the death of your relative or friend, did anyone in the hospital explain why they needed to do it?**	Yes ☐1	No ☐0	Don't know ☐9

How satisfied are you with the way the hospital gave you information about the post mortem?		Poor ⬇								Excellent ⬇	No information given
E4.1	**It was clear and simple**	☐1	☐2	☐3	☐4	☐5	☐6	☐7	☐8	☐9	☐10	☐99
E4.2	**It was explained in a sensitive manner**	☐1	☐2	☐3	☐4	☐5	☐6	☐7	☐8	☐9	☐10	☐99
E4.3	**It was given without delay**	☐1	☐2	☐3	☐4	☐5	☐6	☐7	☐8	☐9	☐10	☐99

Section F: Comments on the care of your relative or friend

F1 **What went well in the care of your relative or friend during their last week of life?**

____

____

____

F2 **What did not go so well in the care of your relative or friend during their last week of life?**

____

____

____

F3 **What could the hospital have done better in the care of your relative or friend during their last week of life?**

____

____

____

Please write any other comments you have:

____

____

____

Section G: Final thoughts

G1	How would you generally describe the care that people get when they die in Irish hospitals?	Very poor ☐1	Poor ☐2	Average ☐3	Good ☐4	Very good ☐5	Don't know ☐9

If you were dying or in the last stages of a terminal illness, what do you think are the three most important things about your care? Write '1' opposite your first choice, '2' opposite your second choice, and '3' opposite your third choice.		
G2.1	To be free from pain	
G2.2	To be conscious and able to communicate	
G2.3	To be surrounded by people I love	
G2.4	To be at home	
G2.5	To be have medical and nursing support readily available	
G2.6	To be have spiritual support readily available	
G2.7	To be in a private space	
G2.8	Other (please give more details)	

Thank you very much for taking the time

to fill out this questionnaire.

Please put it in the pre-paid Hospice friendly Hospitals

envelope and post it today.

Appendix 4

Questionnaire 4

Ward Perceptions

(Pink)

Hospice friendly Hospitals PROGRAMME

Questionnaire 4: Survey of Ward Perceptions on End-of-Life Care

---

Guide to Instruments

Note: The Ward Perceptions Questionnaire is identical to Part Two of the Patient Profile Questionnaire (Nurse Version), except for the addition of C7.

Question	Concept / Domain	Instrument
	Hospital ID	This number will be allocated to each hospital by the H*f*H Programme, and will remain confidential to each hospital.
	Ward ID	This number (from one onwards) will be allocated by the designated audit manager in each hospital, with a separate number allocated to each ward in the audit.
	Questionnaire ID	This number (from one onwards) will be allocated by the designated audit manager in each hospital, with a separate number allocated to each questionnaire in the audit.
**SECTION A: BACKGROUND**		
A1	Position within the ward	Bespoke question. Same question as used in Patient Profile Questionnaire (Nurse Version, Section A), Patient Profile Questionnaire (Doctor Version, Section F), and Hospital Perceptions Questionnaire (Section A).
A2-3	Length of time working in hospital and ward	
A4	Country where brought up	Categories taken from Yeates[84] which summarises the country of origin of nurses in Ireland. Same question as used in Patient Profile Questionnaire (Nurse Version, Section A), and Hospital Perceptions Questionnaire (Section A).
A5	Gender of respondent	Bespoke question. Same question as used in Patient Profile Questionnaire (Nurse Version, Section A), Patient Profile Questionnaire (Doctor Version, Section F), and Hospital Perceptions Questionnaire (Section A).
A6	Age of respondent	
A7	Is English your first language?	
**SECTION B: GENERAL ATTITUDES TO DYING AND DEATH**		
B1	Feeling comfortable about discussing death or dying	Based on questions used in 2004 nationwide survey of public attitudes and experiences regarding death and dying[85]. Same questions used in Patient Profile Questionnaire (Nurse Perceptions B1-4), and in Survey of Hospital Perceptions (B1-4).
B2	Feeling comfortable about discussing bereavement	
B3	Preferred place of care if dying	
B4	Impression of care for people who are dying or terminally ill in Irish hospitals	

84 Yeates, 2006:15
85 Weafer and Associates Research, 2004:8-9.

**SECTION C: WARD ENVIRONMENT**		
C1	Bed occupancy	Bespoke questions. Same questions used in Patient Profile Questionnaire (Nurse Perceptions C1-6).
C2	Patient turnover	
C3	Patient dependency levels	
C4-5	Level and turnover of staff	
C6	Frequency of death on ward	
C7.1-15	Quality of physical space where patient spent most of the time during last week	Bespoke question. Same question used in Patient Profile Questionnaire (Nurse Version B7.1-16) and Bereaved Relatives Questionnaire (D5.1-16).
**SECTION D: WORKING ENVIRONMENT**		
D1-6	Rating of the ward environment	Bespoke questions. Same questions used in Patient Profile Questionnaire (Nurse Version D1-6).
D7	Satisfaction with current work situation	Bespoke question. Same question used in Patient Profile Questionnaire (Nurse Version D7).
**SECTION E: END-OF-LIFE CARE ON THIS WARD**		
E1-16	Rating of ward on different aspects of end-of-life care	Bespoke question but informed by standards for end-of-life care[86], including draft H*f*H standards; studies on end-of-life care[87], and the general public's preferences for end-of-life care[88]. Same question used in Patient Profile Questionnaire (Nurse Version E1-16).
E17.1-2	Acceptability of circumstances in which patients generally die on this ward	Based on question used in survey of death in French hospitals[89]. Response format changed from 'yes/no' to a 10-point scale. Same question – focused on individual patients - used in Patient Profile Questionnaire (Nurse Version J8.1-2) , Patient Profile Questionnaire (Doctor Version D6.1-2) and Survey of Bereaved Relatives (D6-7). Same question –focused on ward - used in Patient Profile Questionnaire (Nurse Version E17.1-2) and Survey of Ward Perceptions (E17.1-2). Same question – focused on hospital - used in Survey of Hospital Perceptions (D1.1-2).
E18	One suggestion that would significantly improve end-of-life care in this ward	Open-ended question. Same question used in Patient Profile Questionnaire (Nurse Version E18). Same question – but focused on hospital rather than ward - used in Survey of Hospital Perceptions (D2).
**SECTION F: PROFESSIONAL & PERSONAL PREPARATION FOR END-OF-LIFE CARE**		
F1-4	Indicators of professional and personal preparedness for end-of-life care, including receipt of training	Bespoke question. Same question used in Patient Profile Questionnaire (Nurse Version F1-4) and Survey of Hospital Perceptions (E1-4).
**SECTION G: EXPERIENCES FOLLOWING DEATH OF A PATIENT**		
G1	Any experience of particular upset in the past year following the death of a patient	Bespoke questions. Same questions used in Patient Profile Questionnaire (Nurse Version G1-4) and Survey of Hospital Perceptions (F1-4).
G2	Did you feel the need to discuss this experience	
G3	Where did this discussion take place	
G4.1-6	Availability of supports if staff experience particular upset	

86 Health Information and Quality Authority, 2008: 23-24.
87 O'Shea, et al, 2008.
88 Weafer and Associates Research, 2004:23-24.
89 Ferrand, Jabre, Vincent-Genod, et al, 2008.

**SECTION H: EDUCATION, TRAINING AND OTHER SUPPORTS FOR END-OF-LIFE CARE**		
H1-11	Adequacy of education, training and other supports for staff to deal with end-of-life care	Bespoke question but informed by standards for end-of-life care[90], including draft H*f*H standards; studies on end-of-life care[91], and studies of the practice environment for nurses[92]. Same question used in Patient Profile Questionnaire (Nurse Version H1-11) and in Survey of Hospital Perceptions (G1-11).
**SECTION J: HOSPITAL PRIORITIES**		
J1-13	Ranking of various hospital priorities	Bespoke question. Same question used in Patient Profile Questionnaire (Nurse Version G1-13) and Survey of Hospital Perceptions (G1-13).
J14	Classification of hospital's religious ethos	Bespoke question. Same question used in Patient Profile Questionnaire (Nurse Version G14) and Survey of Hospital Perceptions (G14).
**SECTION K: FINAL THOUGHTS**		
K1	The three most important things about end-of-life care	Based on question used in 2004 nationwide survey of public attitudes and experiences regarding death and dying[93]. Same question used in Survey of Hospital Perceptions (J1.1-8).

90 Health Information and Quality Authority, 2008: 23-24.
91 O'Shea, et al, 2008.
92 Yu-Fang Li, et al., 2007.
93 Weafer and Associates Research, 2004:16-17.

Hospice friendly Hospitals PROGRAMME

Questionnaire 4: Survey of ward perceptions on end-of-life care

Thank you for taking the time to complete this questionnaire. The questionnaire is part of a hospital audit of its end-of-life care which will improve the hospital's services for patients, especially patients who spend their last days in hospital. This is an official hospital audit which has been ethically approved and is fully endorsed by the hospital's management team. The audit was created by the Hospice Friendly Hospitals (H*f*H) Programme, which is an initiative of the Irish Hospice Foundation. It is part of a national audit of end-of-life care in acute and community hospitals.

The H*f*H Programme will analyse the information in this audit and they will write a confidential report for the hospital. They will not use any names or other signifiers in any published reports which could link this information to any particular hospital, to any staff, to any patients, or to their relatives.

The value of the audit depends on the quality of the information you give in this questionnaire. So, it is important that you carefully fill out this questionnaire with accurate and honest answers to each question. **Your answers will stay completely confidential. You should not write your name on the questionnaire.**

Before you begin answering the questionnaire, read these instructions:

- ✓ Check the bottom of this page to make sure it has this information: Hospital ID, Ward ID, Questionnaire ID. If not, contact the Ward Manager (CNM2) immediately. **Do not start unless the questionnaire has this information.**
- ✓ Try not to answer 'Don't know', unless you really need to.
- ✓ Make sure you tick the boxes clearly.
- ✓ Write clearly in the text box (E18).
- ✓ Before putting the questionnaire in the stamped-addressed envelope provided, check that you have answered every question.
- ✓ If you have difficulty completing any part of the questionnaire, contact the Ward Manager (CNM2).
- ✓ Put the completed questionnaire in the stamped-addressed envelope provided, seal it, and return to the Ward Manager (CNM2). They will pass it on to the designated Hospital Audit Manager who will post it to the H*f*H office in Dublin.

Thank you for contributing to your hospital's audit of end-of-life care by completing this questionnaire.

Hospital ID	Ward ID	Questionnaire ID

Section A: Background

A1	**What is your position in the ward?**	☐1 **Nurse manager**	☐2 **Nurse**	☐3 **Health care assistant**

A2	**How long have you been working in this hospital?**	(to the nearest year)

A3	**How long have you been working on this ward?**	(to the nearest year)

A4	**Where were you brought up?**	Australia ☐1	India ☐2	Ireland ☐3	Middle East ☐4	Nigeria ☐5	New Zealand ☐6
		Pakistan ☐7	Philippines ☐8	South Africa ☐9	UK ☐10	US ☐11	Other ☐12

A5	**Are you male or female?**	☐1 Male	☐2 Female

A6	**How old are you?**	(to the nearest year)

A7	**Is English your first language?**	☐1 Yes	☐2 No

Section B: General attitudes to dying and death

		Not at all comfortable	Not very comfortable	Relatively comfortable	Very comfortable	Completely comfortable	Don't know
B1	**How comfortable are you personally with talking about death or dying?**	☐1	☐2	☐3	☐4	☐5	☐9
B2	**How comfortable are you personally with talking to people who have been recently bereaved?**	☐1	☐2	☐3	☐4	☐5	☐9

		In a hospital	In my own home	In a hospice	Nursing home	Other	Don't know
B3	**Where would you want to be cared for if you were dying?**	☐1	☐2	☐3	☐4	☐5	☐9

		Very poor	Poor	Fair	Good	Excellent	Don't know
B4	**How would you describe the overall care given to people who die in Irish hospitals?**	☐1	☐2	☐3	☐4	☐5	☐9

Section C: Ward environment

		Very low	Low	Average	High	Very high
C1	**How would you describe the bed occupancy rate of this ward?**	☐1	☐2	☐3	☐4	☐5

C2	How would you describe patient turnover rates in this ward?	Very low ☐1	Low ☐2	Average ☐3	High ☐4	Very high ☐5

C3	How would you describe patient dependency levels in this ward?	Very low ☐1	Low ☐2	Average ☐3	High ☐4	Very high ☐5

C4	How would you describe the level of staff turnover in this ward?	Very low ☐1	Low ☐2	Average ☐3	High ☐4	Very high ☐5

C5	In your opinion, are there enough nursing staff in this ward?	Definitely not enough	Not enough	Just enough	Definitely enough
		☐1	☐2	☐3	☐4

C6	How often does a patient die on this ward?	Nearly every day	Nearly every week	Nearly every two weeks	Nearly every three weeks	Nearly every month	Less than once a month
		☐1	☐2	☐3	☐4	☐5	☐9

Tell us what you think objectively about these statements about the overall physical space in this ward.		Very poor ↓									Excellent ↓
		1	2	3	4	5	6	7	8	9	10
C7.1	Patients can have private conversations with hospital staff	☐	☐	☐	☐	☐	☐	☐	☐	☐	☐
C7.2	Staff can have private conversations with patients and family members	☐	☐	☐	☐	☐	☐	☐	☐	☐	☐
C7.3	Family members can stay as long as they want	☐	☐	☐	☐	☐	☐	☐	☐	☐	☐
C7.4	Patients can have dignity during their personal care	☐	☐	☐	☐	☐	☐	☐	☐	☐	☐
C7.5	Patients have easy access to the toilet and shower or bath	☐	☐	☐	☐	☐	☐	☐	☐	☐	☐
C7.6	Patients can choose company, or to be alone	☐	☐	☐	☐	☐	☐	☐	☐	☐	☐
C7.7	Patients can see nature	☐	☐	☐	☐	☐	☐	☐	☐	☐	☐
C7.8	Patients can see natural daylight	☐	☐	☐	☐	☐	☐	☐	☐	☐	☐
C7.9	Patients can experience quiet	☐	☐	☐	☐	☐	☐	☐	☐	☐	☐
C7.10	Patients can listen to TV or radio without disturbing others	☐	☐	☐	☐	☐	☐	☐	☐	☐	☐
C7.11	Patients can personalise their space	☐	☐	☐	☐	☐	☐	☐	☐	☐	☐
C7.12	Patients can turn the TV or radio on or off	☐	☐	☐	☐	☐	☐	☐	☐	☐	☐
C7.13	Patients can control the room temperature	☐	☐	☐	☐	☐	☐	☐	☐	☐	☐
C7.14	Patients can control the light in the room	☐	☐	☐	☐	☐	☐	☐	☐	☐	☐
C7.15	Patients can control the air in the room	☐	☐	☐	☐	☐	☐	☐	☐	☐	☐

Section D: Working environment

In general, how would you rate these aspects of this ward?		Very poor ↓ 1	2	3	4	5	6	7	8	9	Excellent ↓ 10
D1	What is this ward like as a place to work?	☐	☐	☐	☐	☐	☐	☐	☐	☐	☐
D2	What are the quality of relationships between all staff on this ward?	☐	☐	☐	☐	☐	☐	☐	☐	☐	☐
D3	How well equipped is this ward, such as facilities, equipment etc.?	☐	☐	☐	☐	☐	☐	☐	☐	☐	☐
D4	What is the standard of care given to patients on this ward?	☐	☐	☐	☐	☐	☐	☐	☐	☐	☐
D5	What is this ward like as a place to deliver end-of-life care?	☐	☐	☐	☐	☐	☐	☐	☐	☐	☐
D6	What is the quality of ward management?	☐	☐	☐	☐	☐	☐	☐	☐	☐	☐

		Very dissatisfied ↓ 1	2	3	4	5	6	7	8	9	Very satisfied ↓ 10
D7.1	Overall, how satisfied are you with your current work situation?	☐	☐	☐	☐	☐	☐	☐	☐	☐	☐

Section E: End-of-life care on this ward

From your experience of working in this ward, how would you rate these parts of the ward's end-of-life care?		Very poor ↓ 1	2	3	4	5	6	7	8	9	Very good ↓ 10	Don't know 0
E1	Recognising when a patient needs palliative care rather than curative care	☐	☐	☐	☐	☐	☐	☐	☐	☐	☐	☐
E2	Communicating with patients and relatives in a sensitive, truthful and reassuring way	☐	☐	☐	☐	☐	☐	☐	☐	☐	☐	☐
E3	Communicating and sharing information effectively among hospital staff	☐	☐	☐	☐	☐	☐	☐	☐	☐	☐	☐
E4	Making sure that the patient's end-of-life care is coordinated	☐	☐	☐	☐	☐	☐	☐	☐	☐	☐	☐
E5	Giving patients an opportunity to talk about their worries and wishes	☐	☐	☐	☐	☐	☐	☐	☐	☐	☐	☐
E6	Giving relatives or friends an opportunity to talk about their worries and wishes	☐	☐	☐	☐	☐	☐	☐	☐	☐	☐	☐
E7	Making sure the patient's preferences are respected	☐	☐	☐	☐	☐	☐	☐	☐	☐	☐	☐
E8	Making sure the patient is comfortable, and the ward is properly managing their pain and other symptoms	☐	☐	☐	☐	☐	☐	☐	☐	☐	☐	☐
E9	Comforting a patient who is afraid of dying	☐	☐	☐	☐	☐	☐	☐	☐	☐	☐	☐
E10	Supporting relatives or friends to spend time with the dying patient	☐	☐	☐	☐	☐	☐	☐	☐	☐	☐	☐

From your experience of working in this ward, how would you rate these parts of the ward's end-of-life care?		Very poor ⬇ 1	2	3	4	5	6	7	8	9	Very good ⬇ 10	Don't know 0
E11	Creating a sense of dignity and respect around the moment of the patient's death	☐	☐	☐	☐	☐	☐	☐	☐	☐	☐	☐
E12	Respecting the spiritual needs of people from different religious traditions around death	☐	☐	☐	☐	☐	☐	☐	☐	☐	☐	☐
E13	Removing the person who has died respectfully from the ward	☐	☐	☐	☐	☐	☐	☐	☐	☐	☐	☐
E14	Providing a mortuary that respects the dead	☐	☐	☐	☐	☐	☐	☐	☐	☐	☐	☐
E15	Supporting bereaved relatives with information, advice and counselling as they need it	☐	☐	☐	☐	☐	☐	☐	☐	☐	☐	☐
E16	Having clear policies and procedures for end-of-life care	☐	☐	☐	☐	☐	☐	☐	☐	☐	☐	☐

Generally, based on your experience of working in this ward, do you feel the way patients die in this ward would be acceptable for you, or for your family or friends?		Definitely not acceptable ⬇ 1	2	3	4	5	6	7	8	9	Very acceptable ⬇ 10
E17.1	Acceptable for you?	☐	☐	☐	☐	☐	☐	☐	☐	☐	☐
E17.2	Acceptable for your family or friends?	☐	☐	☐	☐	☐	☐	☐	☐	☐	☐

E18 **Could you suggest one thing that would significantly improve the end-of-life care in this ward?**

____

____

____

Section F: Professional and personal preparation for end-of-life care

F1	**Since qualifying have you gone on a formal training course on end-of-life care or palliative care?**	☐1 Yes	☐0 No

**If yes:**			
F2.1	**What was the name of the course?**		
F2.2	**How long did the course last?**	days	hours
F2.3	**Was the course given by the hospital where you currently work?**	☐1 Yes	☐0 No

F3	**How prepared do you feel, professionally, for dealing with the death of a patient?**	Completely unprepared	Fairly unprepared	Reasonably prepared	Completely prepared
		☐1	☐2	☐3	☐4

F4	**How prepared do you feel, personally or emotionally, for dealing with the death of a patient?**	Completely unprepared	Fairly unprepared	Reasonably prepared	Completely prepared
		☐1	☐2	☐3	☐4

Section G: Experiences after the death of a patient

G1	**In the past year, have you been very upset after a patient's death?**	☐1 Yes	☐0 No

G2	**If yes, did you feel the need to talk to someone about it?**	☐1 Yes	☐0 No

G3	**If yes, did you talk to someone?**	Yes, to someone in the hospital	Yes, to someone outside the hospital	No
		☐1	☐2	☐0

**In the future if you were very upset after the death of a patient, what supports could you get?**				
G4.1	**Counselling organised by hospital**	☐1 Yes	☐0 No	☐9 Don't know
G4.2	**Colleagues available and willing to talk and listen to me**	☐1 Yes	☐0 No	☐9 Don't know
G4.3	**Managers available and willing to talk and listen to me**	☐1 Yes	☐0 No	☐9 Don't know
G4.4	**I could change shift patterns**	☐1 Yes	☐0 No	☐9 Don't know
G4.5	**I could take time off work**	☐1 Yes	☐0 No	☐9 Don't know
G4.6	**Other support (write details):**	☐1 Yes	☐0 No	☐9 Don't know

Section H: Education, training and other supports for end-of-life care

**From your experience of working in this hospital, rate these supports offered to staff who are dealing with end-of-life care?**		Not good enough ↓									Good enough ↓	Don't know
		**1**	**2**	**3**	**4**	**5**	**6**	**7**	**8**	**9**	**10**	**0**
H1	**Hospital offers training on the care of patient and family at the patient's end-of-life**	☐	☐	☐	☐	☐	☐	☐	☐	☐	☐	☐
H2	**Hospital offers training in communication skills on dying, death, and bereavement, including breaking bad news to people**	☐	☐	☐	☐	☐	☐	☐	☐	☐	☐	☐
H3	**Hospital offers training in what people from different cultures expect at death**	☐	☐	☐	☐	☐	☐	☐	☐	☐	☐	☐
H4	**Hospital offers courses on understanding the effects of loss, grief and bereavement on people**	☐	☐	☐	☐	☐	☐	☐	☐	☐	☐	☐
H5	**Hospital offers courses on understanding the legal and ethical issues around end-of-life care**	☐	☐	☐	☐	☐	☐	☐	☐	☐	☐	☐
H6	**Hospital offers specialist knowledge and support through its palliative care service**	☐	☐	☐	☐	☐	☐	☐	☐	☐	☐	☐
H7	**Hospital offers opportunities for debriefing, reflection and counseling**	☐	☐	☐	☐	☐	☐	☐	☐	☐	☐	☐
H8	**Hospital encourages positive inter-disciplinary team working**	☐	☐	☐	☐	☐	☐	☐	☐	☐	☐	☐
H9	**Hospital holds post-death reviews**	☐	☐	☐	☐	☐	☐	☐	☐	☐	☐	☐
H10	**Managers show leadership in improving end-of-life care**	☐	☐	☐	☐	☐	☐	☐	☐	☐	☐	☐
H11	**Hospital has clear policies and procedures on dying, death and bereavement**	☐	☐	☐	☐	☐	☐	☐	☐	☐	☐	☐

Section J: Hospital priorities

From your experience of working in this hospital, how much attention does the hospital pay to these things?		Very little attention ↓ 1	2	3	4	5	6	7	8	9	A lot of attention ↓ 10	Don't know 0
J1	**Active treatment of patient's illness**	☐	☐	☐	☐	☐	☐	☐	☐	☐	☐	☐
J2	**Optimising the quality of life for each patient**	☐	☐	☐	☐	☐	☐	☐	☐	☐	☐	☐
J3	**Ensuring the quality of its end-of-life care**	☐	☐	☐	☐	☐	☐	☐	☐	☐	☐	☐
J4	**Controlling infection**	☐	☐	☐	☐	☐	☐	☐	☐	☐	☐	☐
J5	**Developing a person-centred approach to patients**	☐	☐	☐	☐	☐	☐	☐	☐	☐	☐	☐
J6	**Developing a person-centred approach to staff**	☐	☐	☐	☐	☐	☐	☐	☐	☐	☐	☐
J7	**Increasing patient independence and decision-making**	☐	☐	☐	☐	☐	☐	☐	☐	☐	☐	☐
J8	**Making sure that all patients are treated equally**	☐	☐	☐	☐	☐	☐	☐	☐	☐	☐	☐
J9	**Giving staff opportunities to develop their career**	☐	☐	☐	☐	☐	☐	☐	☐	☐	☐	☐
J10	**Supporting staff who give end-of-life care**	☐	☐	☐	☐	☐	☐	☐	☐	☐	☐	☐
J11	**Making sure the hospital's beliefs and principles are respected**	☐	☐	☐	☐	☐	☐	☐	☐	☐	☐	☐
J12	**Avoiding legal risks and being open to legal claims**	☐	☐	☐	☐	☐	☐	☐	☐	☐	☐	☐
J13	**Carrying out innovative research**	☐	☐	☐	☐	☐	☐	☐	☐	☐	☐	☐

J14	**Overall, how would you describe the religious ethos of this hospital?**	Non-religious ☐1	Fairly religious ☐2	Very religious ☐3

Section K: Final thoughts

What do you think are the three most important things about the care available to you if you were dying or in the last stages of a terminal illness?

Write the number '1' opposite your first choice, the number '2' opposite your second choice, and the number '3' opposite your third choice.

K1.1	**To be free from pain**	
K1.2	**To be conscious and able to communicate**	
K1.3	**To be surrounded by people I love**	
K1.4	**To be at home**	

K1.5	**To be have medical and nursing support readily available**	
K1.6	**To be have spiritual support readily available**	
K1.7	**To be in a private space**	
K1.8	**Other (write details)**	

Thank you very much for taking the time to complete this questionnaire.

Put it in the pre-paid H*f*H envelope, seal the envelope, and return to the Ward Manager (CNM2) who will return it to the designated Hospital Audit Manager who will post it to the H*f*H Programme.

Appendix 5

Questionnaire 5

Hospital Perceptions

(Purple)

Hospice friendly Hospitals PROGRAMME

Questionnaire 5:
Survey of Hospital Perceptions on End-of-Life Care

Guide to Instruments

Question	Concept / Domain	Instrument
	Hospital ID	This number will be allocated to each hospital by the H*f*H Programme, and will remain confidential to each hospital.
	Ward ID	This number (from one onwards) will be allocated by the designated audit manager in each hospital, with a separate number allocated to each ward in the audit.
	Questionnaire ID	This number (from one onwards) will be allocated by the designated audit manager in each hospital, with a separate number allocated to each questionnaire in the audit.
**SECTION A: BACKGROUND INFORMATION ON STAFF**<br>Note: Same questions as in Patient Profile Questionnaire (Nurse & Doctor Versions) and Ward Perceptions Questionnaire.		
A1	Position within the hospital	Bespoke question. Same question as used in Patient Profile Questionnaire (Nurse Version, Section A), Patient Profile Questionnaire (Doctor Version, Section F), Ward Perceptions Questionnaire (Section A), and Hospital Perceptions Questionnaire (Section A).
A2	Length of time working in hospital	
A3	Country where brought up	Categories taken from Yeates[94] which summarises the country of origin of nurses in Ireland. Same question as used in Patient Profile Questionnaire (Nurse Version, Section A), Ward Perceptions Questionnaire (Section A), and Hospital Perceptions Questionnaire (Section A).
A4	Gender of respondent	Bespoke question. Same question as used in Patient Profile Questionnaire (Nurse Version, Section A), Patient Profile Questionnaire (Doctor Version, Section F), Ward Perceptions Questionnaire (Section A), and Hospital Perceptions Questionnaire (Section A).
A5	Age of respondent	
A6	Is English your first language?	
**SECTION B: GENERAL ATTITUDES TO DYING AND DEATH**		
B1	Feeling comfortable about discussing death or dying	Based on questions used in 2004 nationwide survey of public attitudes and experiences regarding death and dying[95]. Same questions used in Patient Profile Questionnaire (Nurse Perceptions B1-4), and in Survey of Ward Perceptions (B1-4).
B2	Feeling comfortable about discussing bereavement	
B3	Preferred place of care if dying	
B4	Impression of care for people who are dying or terminally ill in Irish hospitals	

94 Yeates, 2006:15
95 Weafer and Associates Research, 2004:8-9.

**SECTION C: WORKING ENVIRONMENT**		
C1	Satisfaction with current work situation	Bespoke question. Same question used in Patient Profile Questionnaire (Nurse Version, D6) and Ward Perceptions Questionnaires (D6).
**SECTION D: END-OF-LIFE CARE ON THIS HOSPITAL**		
D1.1-2	Acceptability of circumstances in which patients generally die in hospital	Based on question used in survey of death in French hospitals[96]. Response format changed from 'yes/no' to a 10-point scale. Same question – focused on individual patients - used in Patient Profile Questionnaire (Nurse Version J8.1-2) , Patient Profile Questionnaire (Doctor Version D6.1-2) and Survey of Bereaved Relatives (D6-7). Same question – focused on ward - used in Patient Profile Questionnaire (Nurse Version E17.1-2) and Survey of Ward Perceptions (E17.1-2). Same question – focused on hospital - used in Survey of Hospital Perceptions (D1.1-2).
D2	One suggestion that would significantly improve end-of-life care in this hospital	Open-ended question. Same question – but focused on wards rather than hospital - used in Patient Profile Questionnaire (Nurse Version E18) and Survey of Ward Perceptions (E18).
**SECTION E: PROFESSIONAL & PERSONAL PREPARATION FOR END-OF-LIFE CARE**		
E1-4	Indicators of professional and personal preparedness for end-of-life care, including receipt of training	Bespoke question. Same question used in Patient Profile Questionnaire (Nurse Version F1-4) and Survey of Ward Perceptions (F1-4).
**SECTION F: EXPERIENCES FOLLOWING THE DEATH OF A PATIENT**		
F1	Any experience of particular upset in the past year	Bespoke questions. Same questions used in Survey of Ward Perceptions (G1-4), and in Patient Profile Questionnaire (Nurse Version G1-4).
F2	Did you feel the need to discuss this experience	
F3	Where did you discuss this experience	
F4.1-6	Availability of supports if staff experience particular upset	
**SECTION G: EDUCATION, TRAINING AND OTHER SUPPORTS FOR END-OF-LIFE CARE**		
G1-11	Adequacy of education, training and other supports for staff to deal with end-of-life care	Bespoke question but informed by standards for end-of-life care[97], including draft H*f*H standards; studies on end-of-life care[98], and studies of the practice environment for nurses[99]. Same question used in Survey of Ward Perceptions (G1-11), and in Patient Profile Questionnaire (Nurse Version H1-11).
**SECTION H: HOSPITAL PRIORITIES**		
H1-13	Ranking of various hospital priorities	Bespoke question. Same question used in Survey of Ward Perceptions (G1-13), and in Patient Profile Questionnaire (Nurse Version G1-13).
H14	Classification of hospital's religious ethos	Bespoke question. Same question used in Survey of Ward Perceptions (G14), and in Patient Profile Questionnaire (Nurse Version G14).
**SECTION J: FINAL THOUGHTS**		
J1.1-8	The three most important things about end-of-life care	Based on question used in 2004 nationwide survey of public attitudes and experiences regarding death and dying[100]. Same question used in Survey of Ward Perceptions (K1.1-8).

96 Ferrand, Jabre, Vincent-Genod, et al, 2008.
97 Health Information and Quality Authority, 2008: 23-24.
98 O'Shea, et al, 2008.
99 Yu-Fang Li, et al., 2007.
100 Weafer and Associates Research, 2004:16-17.

Questionnaire 5:
Survey of hospital perceptions on end-of-life care(v9)

Thank you for taking the time to complete this questionnaire. The questionnaire is part of a hospital audit of its end-of-life care which will improve the hospital's services for patients, especially patients who spend their last days in hospital. This is an official hospital audit which has been ethically approved and is fully endorsed by the hospital's management team. The audit was created by the Hospice Friendly Hospitals (H*f*H) Programme, an initiative of the Irish Hospice Foundation. It is part of a national audit of end-of-life care in acute and community hospitals.

The H*f*H Programme will analyse the information from this audit and they will write a confidential report for the hospital. They will not use any names or other signifiers in any published reports which could link this information to any particular hospital, to any staff, to any patients, or to their relatives.

The value of the audit depends on the quality of the information you give in this questionnaire. So, it is important that you carefully fill out the questionnaire, with accurate and honest answers to each question. **Your answers will stay completely confidential. You should not write your name on the questionnaire.**

Before you begin answering the questionnaire, read these instructions:

- ✓ Check the bottom of this page to make sure it has the Hospital ID and the Questionnaire ID. If not, contact the designated Hospital Audit Manager immediately. **Do not start answering the questions unless the questionnaire has this information.**
- ✓ Try not to answer 'Don't know', unless you really need to.
- ✓ Make sure you tick the boxes clearly and write clearly in the text box (D2).
- ✓ Before putting the questionnaire in the stamped-addressed envelope provided, check that you have answered every question.
- ✓ If you have difficulty completing any part of the questionnaire, contact the designated Hospital Audit Manager.
- ✓ Put the completed questionnaire in the stamped-addressed envelope provided, seal it, and return to the designated Hospital Audit Manager who will post it to the H*f*H office in Dublin.

Thank you for contributing to your hospital's audit of end-of-life care by completing this questionnaire.

Hospital ID	Questionnaire ID

Section A: Background

A1	What is your position or department in the hospital?			
	☐1	CEO or General manager	☐11	Catering staff
	☐2	Nursing management (DoN, Ass DoN,)	☐12	Household staff
	☐3	Clinical Nurse Managers / Clinical Nurse Specialists	☐13	Mortuary staff
	☐4	Consultant doctors	☐14	Security staff
	☐5	Non-consultant doctors	☐15	Complaints officer or Patient advice and liaison officer
	☐6	Allied health professionals<br>(including social worker, physiotherapist, occupational therapist, speech therapist)	☐16	Ward clerk
	☐7	Pastoral carers	☐17	Reception
	☐8	Bereavement coordinators	☐18	Administration<br>(excluding complaints officer or patient advice and liaison officer, ward clerk, and reception)
	☐9	End-of-life care coordinators	☐19	Other
	☐10	Porters		

A2	How long have you been working in this hospital?	(to the nearest year)

A4	Where were you brought up?	Australia	India	Ireland	Middle East	Nigeria	New Zealand
		☐1	☐2	☐3	☐4	☐5	☐6
		Pakistan	Philippines	South Africa	UK	US	Other
		☐7	☐8	☐9	☐10	☐11	☐12

A4	Are you male or female?	☐1Male	☐2Female

A5	How old are you?	(to the nearest year)

A6	Is English your first language?	☐1 Yes	☐2 No

Section B: General attitudes to dying and death

		Not at all comfortable	Not very comfortable	Relatively comfortable	Very comfortable	Completely comfortable	Don't know
B1	**How comfortable are you personally with talking about death or dying?**	☐1	☐2	☐3	☐4	☐5	☐9
B2	**How comfortable are you personally with talking to people who have been recently bereaved?**	☐1	☐2	☐3	☐4	☐5	☐9

		In a hospital	In my own home	In a hospice	Nursing home	Other	Don't know
B3	**Where would you want to be cared for if you were dying?**	☐1	☐2	☐3	☐4	☐5	☐9

		Very poor	Poor	Fair	Good	Excellent	Don't know
B4	**How would you describe the overall care given to people who die in Irish hospitals?**	☐1	☐2	☐3	☐4	☐5	☐9

Section C: Working environment

		1 Very dissatisfied	2	3	4	5	6	7	8	9	10 Very satisfied
C1	**Overall, how satisfied are you with your current work situation?**	☐	☐	☐	☐	☐	☐	☐	☐	☐	☐

Section D: End-of-life care in this hospital

**Generally, based on your experience of working in this hospital, do you feel the way patients die in this hospital would be acceptable for you, or for your family or friends?**		1 Definitely not acceptable	2	3	4	5	6	7	8	9	10 Very acceptable
D1.1	**Acceptable for you?**	☐	☐	☐	☐	☐	☐	☐	☐	☐	☐
D1.2	**Acceptable for your family or friends?**	☐	☐	☐	☐	☐	☐	☐	☐	☐	☐

D2 **Could you suggest one thing that would significantly improve end-of-life care in this hospital?**

____

____

____

Section E: Professional and personal preparation for end-of-life care

E1	**Since qualifying, have you gone on a formal training course on end-of-life care or palliative care?**	☐1 Yes	☐0 No

**If yes:**			
E2.1	**What was the name of the course?**		
E2.2	**How long did the course last?**	days	hours
E2.3	**Was the course given by the hospital where you currently work?**	☐1 Yes	☐0 No

E3	**How prepared do you feel, professionally, for dealing with the death of a patient?**	Completely unprepared ☐1	Fairly unprepared ☐2	Reasonably prepared ☐3	Completely prepared ☐4

E4	**How prepared do you feel, personally or emotionally, for dealing with the death of a patient?**	Completely unprepared ☐1	Fairly unprepared ☐2	Reasonably prepared ☐3	Completely prepared ☐4

Section F: Experiences after the death of a patient

F1	**In the past year, have you been very upset after a patient's death?**	☐1 Yes	☐0 No

F2	**If yes, did you feel the need to talk to someone about this?**	☐1 Yes	☐0 No

F3	**If yes, did you talk to someone?**	Yes, to someone in the hospital	Yes, to someone outside the hospital	No
		☐1	☐2	☐0

**In the future if you were very upset after the death of a patient, what supports could you get?**				
F4.1	**Counselling organised by hospital**	☐1 Yes	☐0 No	☐9 Don't know
F4.2	**Colleagues available and willing to talk and listen to me**	☐1 Yes	☐0 No	☐9 Don't know
F4.3	**Managers available and willing to talk and listen to me**	☐1 Yes	☐0 No	☐9 Don't know
F4.4	**I could change shift patterns**	☐1 Yes	☐0 No	☐9 Don't know
F4.5	**I could take time off work**	☐1 Yes	☐0 No	☐9 Don't know
F4.6	**Other support (write details):**	☐1 Yes	☐0 No	☐9 Don't know

Section G: Education, training and other supports for end-of-life care

From your experience of working in this hospital, rate these supports offered to staff who are dealing with end-of-life care?		Inadequate ↓									Adequate ↓	Don't know
		1	2	3	4	5	6	7	8	9	10	0
G1	**Hospital offers training on the care of patient and family at the patient's end-of-life**	☐	☐	☐	☐	☐	☐	☐	☐	☐	☐	☐
G2	**Hospital offers training in communication skills on dying, death, and bereavement, including breaking bad news to people**	☐	☐	☐	☐	☐	☐	☐	☐	☐	☐	☐
G3	**Hospital offers training in what people from different cultures expect at death**	☐	☐	☐	☐	☐	☐	☐	☐	☐	☐	☐
G4	**Hospital offers courses on understanding the effect of loss, grief and bereavement on people**	☐	☐	☐	☐	☐	☐	☐	☐	☐	☐	☐
G5	**Hospital offers courses on understanding the legal and ethical issues around end-of-life care**	☐	☐	☐	☐	☐	☐	☐	☐	☐	☐	☐
G6	**Hospital offers specialist knowledge and support through its palliative care service**	☐	☐	☐	☐	☐	☐	☐	☐	☐	☐	☐
G7	**Hospital offers opportunities for debriefing, reflection and counseling**	☐	☐	☐	☐	☐	☐	☐	☐	☐	☐	☐
G8	**Hospital encourages positive inter-disciplinary team working**	☐	☐	☐	☐	☐	☐	☐	☐	☐	☐	☐
G9	**Hospital holds post-death reviews**	☐	☐	☐	☐	☐	☐	☐	☐	☐	☐	☐
G10	**Managers show leadership in improving end-of-life care**	☐	☐	☐	☐	☐	☐	☐	☐	☐	☐	☐
G11	**Hospital has clear policies and procedures on dying, death and bereavement**	☐	☐	☐	☐	☐	☐	☐	☐	☐	☐	☐

Section H: Hospital priorities

From your experience of working in this hospital, how much attention does the hospital pay to these things?		Very little attention ↓									A lot of attention ↓	Don't know
		1	2	3	4	5	6	7	8	9	10	0
H1	**Active treatment of patient's illness**	☐	☐	☐	☐	☐	☐	☐	☐	☐	☐	☐
H2	**Optimising the quality of life for each patient**	☐	☐	☐	☐	☐	☐	☐	☐	☐	☐	☐
H3	**Ensuring the quality of its end-of-life care**	☐	☐	☐	☐	☐	☐	☐	☐	☐	☐	☐
H4	**Controlling infection**	☐	☐	☐	☐	☐	☐	☐	☐	☐	☐	☐
H5	**Developing a person-centred approach to patients**	☐	☐	☐	☐	☐	☐	☐	☐	☐	☐	☐
H6	**Developing a person-centred approach to staff**	☐	☐	☐	☐	☐	☐	☐	☐	☐	☐	☐
H7	**Increasing patient independence and decision-making**	☐	☐	☐	☐	☐	☐	☐	☐	☐	☐	☐
H8	**Making sure that all patients are treated equally**	☐	☐	☐	☐	☐	☐	☐	☐	☐	☐	☐
H9	**Giving staff opportunities to develop their career**	☐	☐	☐	☐	☐	☐	☐	☐	☐	☐	☐
H10	**Supporting staff who give end-of-life care**	☐	☐	☐	☐	☐	☐	☐	☐	☐	☐	☐

From your experience of working in this hospital, how much attention does the hospital pay to these things?		Very little attention ↓									A lot of attention ↓	Don't know
		1	2	3	4	5	6	7	8	9	10	0
H11	Making sure the hospital's beliefs and principles are respected	☐	☐	☐	☐	☐	☐	☐	☐	☐	☐	☐
H12	Avoiding legal risks and exposure to legal claims	☐	☐	☐	☐	☐	☐	☐	☐	☐	☐	☐
H13	Carrying out innovative research	☐	☐	☐	☐	☐	☐	☐	☐	☐	☐	☐

H14	Overall, how would you describe the religious ethos of this hospital?	Non-religious	Fairly religious	Very religious
		☐1	☐2	☐3

Section J: Final thoughts

What do you think are the three most important things about the care available to you if you were dying or in the last stages of a terminal illness? Write the number '1' opposite your first choice, the number '2' opposite your second choice, and the number '3' opposite your third choice.		
J1.1	To be free from pain	
J1.2	To be conscious and able to communicate	
J1.3	To be surrounded by people I love	
J1.4	To be at home	
J1.5	To be have medical and nursing support readily available	
J1.6	To be have spiritual support readily available	
J1.7	To be in a private space	
J1.8	Other (write details):	

Thank you very much for taking the time to complete this questionnaire.

Put it in the pre-paid H*f*H envelope, seal the envelope, and return to the designated Hospital Audit Manager who will post it to the H*f*H Programme.

Appendix 6

Questionnaire 6

Hospital Profile

(Brown)

Hospice friendly Hospitals PROGRAMME

Questionnaire 6: Hospital Profile

Guide to Instruments

Question	Concept / Domain	Instrument
	Hospital ID	This number will be allocated to each hospital by the H*f*H Programme, and will remain confidential to each hospital.
**SECTION A: ACCOMMODATION**		
A1	Number of inpatient and day beds	HIPE question
A2	Number of beds in single rooms and multi-occupancy rooms	Bespoke question
A3	Average bed-occupancy level	Bespoke question
**SECTION B: PATIENTS**		
B1	Number of patients discharged in 2008	HIPE question
B2	Number of public and private patients discharged in 2008	HIPE question
B3	Number of patients with a Medical Card discharged in 2008	HIPE question
**SECTION C: DEATHS**		
C1	Acute hospitals only: Deaths in 2008	Bespoke question
C2	Acute hospitals only: 'brought in dead' in 2008	Bespoke question
C3	Community hospitals only: Deaths in 2008	Bespoke question
C4	Post mortems in 2008	Bespoke question
**SECTION D: STAFF**		
D1.1-18	Number of whole-time-equivalent staff on 31st December 2008	Bespoke question
D2.1-18	Number of number of staff who in December 2008 who were employed by the hospital for less than one year	Bespoke question to measure staff turnover
D3.1-18	Number of number of days lost in 2008 due to sickness and other reasons, excluding annual leave	Bespoke question to measure absenteeism
**SECTION E: SPECIALIST PALLIATIVE CARE SERVICE**		
E1	Does hospital have a specialist palliative care service	Bespoke question.
E2.1-15	Categories of staff on the specialist palliative care service	Bespoke question. Staff categories taken from Chapter Six of the national baseline study on palliative care published in 2006 but based on 2004 data[101].
E3	Does the hospital use the Liverpool Care Pathway	Bespoke question.

101 Murray, Sweeney, Smyth, and Conolly, 2006.

**SECTION F: FACILITIES IN HOSPITAL AND WARDS**		
F1	Year hospital was originally built	Bespoke question
F2.1-22	Facilities in hospital	Informed by the H*f*H Guidelines on Design and Dignity Guidelines for Physical Environments of Hospitals Supporting End-of-Life Care[102].
**SECTION G: COMPLAINTS**		
G1	Number of official complaints in 2008	Bespoke question
G2	Number of official complaints in 2008 related to end-of-life issues	Bespoke question
**SECTION G: POLICIES AND PROCEDURES SUPPORTING END-OF-LIFE SERVICES**		
H1	Does the hospital have a document outlining its policies and procedures for end-of-life care?	Bespoke question
H2	Does hospital's current business plan have a separate section devoted to services on dying, death and bereavement?	Bespoke question
H3.1-3	Is hospital in the H*f*H Programme	Bespoke question
H4.1-3	Does the hospital have a Standing Committee on dying, death, and bereavement – or equivalent?	Bespoke question
**SECTION J: TRAINING AND SUPPORTING STAFF IN END-OF-LIFE CARE**		
J1.1-7	Does hospital provide training on dying, death and bereavement	Bespoke question
J2	Does the hospital have a document outlining the supports that are available to staff involved in end-of-life services?	Bespoke question
**SECTION K: MORTUARY**		
K1	Does hospital have a mortuary	Bespoke question
K2.1-22	Facilities in mortuary	Informed by the H*f*H Guidelines on Design and Dignity Guidelines for Physical Environments of Hospitals Supporting End-of-Life Care[103].
**SECTION L: BEREAVEMENT SERVICES**		
L1	Does hospital have a bereavement service	Bespoke question
L2.1-6	Characteristics of bereavement service	Bespoke question

102 Hospice Friendly Hospitals, 2008
103 Hospice Friendly Hospitals, 2008

Questionnaire 6:
Hospital profile

Thank you for taking part in the audit of end-of-life care in your hospital. This is the first ever audit of end-of-life care in Irish hospitals and it is probably the most comprehensive one being done in the European Union. The audit was developed by the Hospice friendly Hospitals (H*f*H) Programme, which is an initiative of the Irish Hospice Foundation, and is in partnership with the HSE, the Atlantic Philanthropies, Health Services National Partnership Forum and the Dormant Accounts Fund.

The H*f*H Programme will analyse the information in the audit and they will write a confidential report for the hospital. They will not use any names or other signifiers in any published reports which could link this information to any particular hospital, to any staff, to any patients, or to their relatives. The value of the audit depends on the quality of the information you give in this questionnaire. So, it is important that you carefully fill out the questionnaire with accurate and honest answers to each question.

Before beginning to answer the questionnaire, read these instructions:

- ✓ Before you start, check the bottom of this page to make sure it has the Hospital ID.
- ✓ Try not to answer 'Don't know', unless you really need to.
- ✓ Check that you have answered every question.
- ✓ If you have difficulty completing any part of the questionnaire, contact the designated Hospital Audit Manager.
- ✓ Some of the questions involve matters of judgement, such as questions about the extent to which the hospital meets certain standards for end-of-life care (Sections F, J, K and L). In view of this, the answers should reflect the agreed assessment of hospital management.
- ✓ It is important that the senior management in the hospital sees and approves your completed questionnaire.
- ✓ The designated Hospital Audit Manager will do a final check on the questionnaire before posting it to the H*f*H office in Dublin.

Thank you for contributing to your hospital's audit of end-of-life care by completing this questionnaire.

Hospital ID number:	Name of designated Hospital Audit Manager:

Section A: Accommodation

		In-patient beds	Day-beds	Total
A1	**How many beds were in the hospital on 31st December 2008?**			

		Number of beds in single rooms	Number of beds in multi-occupancy rooms	Total
A2	**How many of these beds were in single rooms and multi-occupancy rooms?**			

A3	**What was the average bed-occupancy* level of the hospital in 2008?**	Average bed occupancy:
	*You can work out the average bed occupancy rate in the hospital by dividing the total number of occupied bed-days by the total number of available bed-days, multiplied by 100.	

Section B: Patients

		In-patients	Day-patients*	Total
B1	**How many in-patients and day-patients were discharged from the hospital in 2008?**			
	*A day-patient is someone admitted on an elective basis and does not need the use of a hospital bed overnight.			

		Public patients	Private patients	Total
B2	**How many in-patients were discharged in 2008? How many were public patients and private patients?**			

B3	**How many in-patients who were discharged from the hospital in 2008 had a Medical Card?**	

Section C: Deaths

**This question is for acute hospitals only**			
**How many deaths happened in the hospital in 2008, and how many were referred to the coroner?**<br><br>**Give the numbers for these categories:**		Deaths	Referred to coroner
C1.1	**Deaths in Accident and Emergency department**		
C1.2	**Death in intensive care, which includes ICU, ITU, CCU, HDU, NITU, theatre, etc.**		
C1.3	**Deaths in other wards**		
C1.4	**Total**		

This question is for acute hospitals only			
How many deaths in your hospital in 2008 were followed by a post-mortem? Give the numbers for these categories:		Hospital post-mortem	Coroner's post-mortem
C2.1	Deaths in Accident and Emergency Department		
C2.2	Deaths in intensive care, which includes ICU, ITU, CCU, HDU, NITU, theatre, etc.		
C2.3	Deaths in other wards		
C2.4	Total		

This question is for acute hospitals only		
How many deaths in 2008 were 'brought in dead'? Give the numbers for each:		Number of 'brought in dead'
C3.1	People pronounced dead outside hospital and brought to Accident and Emergency	
C3.2	People pronounced dead outside hospital and brought directly to the mortuary	
C3.3	People pronounced dead outside hospital and brought in for preparation by funeral directors	
C3.4	Total	

These questions are for community hospitals only		
C4.1	How many deaths happened in the hospital in 2008?	
C4.2	How many of your patients or residents died in an acute hospital in 2008?	
C4.3	How many deaths in your hospital in 2008 were followed by a hospital post-mortem?	
C4.4	How many deaths in your hospital in 2008 were followed by a coroner's post-mortem?	

Section D: Staff

How many whole-time-equivalent staff (WTE) are in the hospital on 31st December 2008?<br><br>Also, give the actual number of staff in each category, not including agency staff.		WTE	Actual number of staff
D1.1	**CEO or General manager**		
D1.2	**Nursing management (DoN, Ass DoN,)**		
D1.3	**Consultant doctors**		
D1.4	**Non-consultant doctors**		
D1.5	**Clinical Nurse Managers / Clinical Nurse Specialists**		
D1.6	**Allied health professionals**<br><br>**(including social worker, physiotherapist, occupational therapist, speech therapist)**		
D1.7	**Pastoral carers**		
D1.8	**Bereavement coordinators**		
D1.9	**End-of-life care coordinators**		
D1.10	**Porters**		
D1.11	**Catering staff**		
D1.12	**Household staff**		
D1.13	**Mortuary staff**		
D1.14	**Security staff**		
D1.15	**Complaints officer or Patient advice and liaison officer**		
D1.16	**Ward clerk**		
D1.17	**Reception**		
D1.18	**Administration**<br><br>**(excluding complaints officer or patient advice and liaison officer, ward clerk, and reception)**		
D1.19	**Nurses (excluding nursing management, CNMs, CNSs and agency staff)**		
D1.20	**Healthcare Assistants**		
D1.21	**Other**		
D1.22	**TOTAL**		

How many staff were employed by the hospital for less than one year in December 2008? Do not include agency staff.		Number of staff employed for less than one year in December 2008
D2.1	**CEO or General manager**	
D2.2	**Nursing management (DoN, Ass DoN)**	
D2.3	**Consultant doctors**	
D2.4	**Non-consultant doctors**	
D2.5	**Clinical Nurse Managers / Clinical Nurse Specialists**	
D2.6	**Allied health professionals**<br><br>**(including social worker, physiotherapist, occupational therapist, speech therapist)**	
D2.7	**Pastoral carers**	
D2.8	**Bereavement coordinators**	
D2.9	**End-of-life care coordinators**	
D2.10	**Porters**	
D2.11	**Catering staff**	
D2.12	**Household staff**	
D2.13	**Mortuary staff**	
D2.14	**Security staff**	
D2.15	**Complaints officer or Patient advice and liaison officer**	
D2.16	**Ward clerk**	
D2.17	**Reception**	
D2.18	**Administration**<br><br>**(excluding complaints officer or patient advice and liaison officer, ward clerk, and reception)**	
D2.19	**Nurses (excluding nursing management, CNMs, CNSs and agency staff)**	
D2.20	**Healthcare Assistants**	
D2.21	**Other**	
D2.22	**TOTAL**	

What is the total number of days lost in 2008 because of sickness and other reasons, excluding annual leave, in each of these categories? Do not include agency staff.		Number of days lost because of sickness and other reasons, not including annual leave
D3.1	**CEO or General manager**	
D3.2	**Nursing management (DoN, Ass DoN)**	
D3.3	**Consultant doctors**	
D3.4	**Non-consultant doctors**	
D3.5	**Clinical Nurse Managers / Clinical Nurse Specialists**	
D3.6	**Allied health professionals**<br>**(including social worker, physiotherapist, occupational therapist, speech therapist)**	
D3.7	**Pastoral carers**	
D3.8	**Bereavement coordinators**	
D3.9	**End-of-life care coordinators**	
D3.10	**Porters**	
D3.11	**Catering staff**	
D3.12	**Household staff**	
D3.13	**Mortuary staff**	
D3.14	**Security staff**	
D3.15	**Complaints officer or Patient advice and liaison officer**	
D3.16	**Ward clerk**	
D3.17	**Reception**	
D3.18	**Administration**<br>**(excluding complaints officer or patient advice and liaison officer, ward clerk, and reception)**	
D3.19	**Nurses (excluding nursing management, CNMs, CNSs and agency staff)**	
D3.20	**Healthcare Assistants**	
D3.21	**Other**	
D3.22	**TOTAL**	

		Not a problem ↓									Major problem ↓
		**1**	**2**	**3**	**4**	**5**	**6**	**7**	**8**	**9**	**10**
D4.1	**Do you think the number of days lost in 2008 because of sickness and other reasons, excluding annual leave, was a problem for this hospital?**	☐	☐	☐	☐	☐	☐	☐	☐	☐	☐

Section E: Specialist palliative care service

E1	**Does the hospital have a specialist palliative care service?**	☐1 Yes	☐0 No

If yes, how many whole-time equivalent staff (WTE) are in the specialist palliative care service on 31st December 2008?		WTE
E2.1	**Consultant in palliative medicine**	
E2.2	**Non-consultant hospital doctors**	
E2.3	**Specialist palliative care nurses or Clinical nurse specialists**	
E2.4	**Physiotherapist**	
E2.5	**Occupational therapist**	
E2.6	**Social worker**	
E2.7	**Pastoral or spiritual care**	
E2.8	**Speech and language therapist**	
E2.9	**Clinical nutritionists or dietitians**	
E2.10	**Pharmacist**	
E2.11	**Care attendants or assistants**	
E2.12	**Volunteer coordinators**	
E2.13	**Librarian or education personnel**	
E2.14	**Administration, such as medical secretaries**	
E2.15	**Other (write details)**	

E3	**Does the hospital use the Liverpool Care Pathway?**	☐1 Yes	☐0 No

Section F: Facilities in the hospital

F1	**In what year was the hospital originally built?**	

Rate how true or untrue these statements are about this hospital.		Untrue 1	2	3	4	5	6	7	8	9	True 10
F2.1	The hospital is on a public transport route										
F2.2	There is enough car-parking for visitors to the hospital										
F2.3	There is a supervised set-down space close to the hospital entrance for patients and relatives										
F2.4	It is easy for patients and relatives to find their way around the hospital										
F2.5	All patients at the end of their life are offered a choice of a single room										
F2.6	There is at least 3.6 metres clear space around each bed										
F2.7	Each ward has a quiet sitting room for patients and relatives										
F2.8	Each ward has a child-friendly TV lounge										
F2.9	Each ward has a meeting room for sensitive private conversations between staff and patients or relatives										
F2.10	Each ward has access to a room suitable for therapies such as art, music, reflexology.										
F2.11	Each ward has enough storage for the personal belongings of deceased patients										
F2.12	There are toilets for relatives on or near each ward										
F2.13	There is a relative’s room with shower and toilet close to each ward										
F2.14	People can get drinking water, hot and cold drinks and snacks near each ward										
F2.15	There is at least one suitably sized multi-faith space in the hospital										
F2.16	There is a citizens information service or information resource area in the hospital										
F2.17	There is a diverse range of artwork displayed throughout the hospital										
F2.18	The hospital has spaces which are used for performing arts or playing music										
F2.19	Patients, relatives and staff have access to outdoor spaces such as gardens, patios, verandas, courtyards, or landscaped areas										
F2.20	The hospital has specific staff rooms										
F2.21	The hospital has meeting rooms for staff										
F2.22	The hospital has a separate dining space for staff										

Section G: Complaints

G1	**How many official complaints were made to the hospital in 2008?**	

G2	**How many of these official complaints in 2008 were about end-of-life issues?**	

Section H: Policies and procedures supporting end-of-life services

H1	**Does the hospital have a document outlining its policies and procedures for its end-of-life care?**	Yes, as a separate document	Yes, as part of another document	No
		☐1	☐2	☐0

H2	**In the hospital's current business plan, are there specific objectives or targets for improving its end-of-life care?**	Yes	No	No business plan
		☐1	☐0	☐3

H3.1	**Has the hospital signed a memorandum of understanding with the Hospice friendly Hospitals (H*f*H) Programme?**	☐1 Yes	☐0 No

H3.2	**Does the hospital have a H*f*H Development Coordinator?**	☐1 Yes	☐0 No

H3.3	**If yes, what year did the H*f*H Development Coordinator start working?**	(year)

H4.1	**Does the hospital have a standing committee on dying, death, and bereavement, or equivalent?**	☐1 Yes	☐0 No
H4.2	**If yes, what year was this committee set up?**	(year)	
H4.3	**If yes, how many meetings of this committee has there been since was it set up?**	meetings	

Section J: Training and supporting staff in end-of-life care

		**Induction training** Only include training that was half a day or more.		**In-service training** Only include training that was half a day or more.	
**Does the hospital give training, either induction or in-service, on any of these aspects of dying, death, and bereavement?**					
J1.1	**Care of the patient and family at the patient's end-of-life**	☐1 Yes	☐0 No	☐1 Yes	☐0 No
J1.2	**Communication skills about dying, death, and bereavement, including breaking bad news to people**	☐1 Yes	☐0 No	☐1 Yes	☐0 No
J1.3	**Training in what people from different cultures expect at death**	☐1 Yes	☐0 No	☐1 Yes	☐0 No
J1.4	**Understanding the impact of loss, grief and bereavement**	☐1 Yes	☐0 No	☐1 Yes	☐0 No
J1.5	**Understanding the legal and ethical issues around end-of-life care**	☐1 Yes	☐0 No	☐1 Yes	☐0 No
J1.6	**Support services for staff who give end-of-life care**	☐1 Yes	☐0 No	☐1 Yes	☐0 No
J1.7	**Other training (write details):**	☐1 Yes	☐0 No	☐1 Yes	☐0 No

J2	**Does the hospital have a document outlining the supports* that are available to staff who are involved in end-of-life services or in traumatic incidents?**	☐1 Yes	☐0 No
	*Supports for staff can be practical or emotional. These can include opportunities for debriefing, a quiet space in the hospital to reflect after a death, or access to counseling, psychological, psychiatric or bereavement support services, either inside or outside the hospital.		

Section K: Mortuary

**K1**	**Does the hospital have a mortuary?**	☐1 Yes	☐0 No

**If yes, does the mortuary have any of these:**			
K2.1	**An outer entrance with protection from the weather**	☐1 Yes	☐0 No
K2.2	**An inner reception area**	☐1 Yes	☐0 No
K2.3	**A waiting room that can hold more than one family**	☐1 Yes	☐0 No
K2.4	**More than one waiting room**	☐1 Yes	☐0 No
K2.5	**A waiting room that has hot and cold drinks**	☐1 Yes	☐0 No
K2.6	**A waiting room that has toilets nearby**	☐1 Yes	☐0 No
K2.7	**A viewing room that can hold several relatives at the same time**	☐1 Yes	☐0 No
K2.8	**A viewing room that can be adapted to the needs of different faiths and cultures**	☐1 Yes	☐0 No
K2.9	**A viewing room that can be adapted for baby or child deaths**	☐1 Yes	☐0 No
K2.10	**A viewing room that has suitable furniture for relatives to stay over-night if they want to**	☐1 Yes	☐0 No
K2.11	**A viewing room where people can wash their hands**	☐1 Yes	☐0 No
K2.12	**A viewing room that has toilets nearby**	☐1 Yes	☐0 No
K2.13	**More than one viewing room**	☐1 Yes	☐0 No
K2.14	**A multi-faith room**	☐1 Yes	☐0 No
K2.15	**A meeting or interview room**	☐1 Yes	☐0 No
K2.16	**A preparatory room for ritual washing of the body or preparation of the body for viewing**	☐1 Yes	☐0 No
K2.17	**A storage area for extra furniture or storage of religious symbols of different faiths**	☐1 Yes	☐0 No
K2.18	**Access to a mortuary garden**	☐1 Yes	☐0 No
K2.19	**The route from the hospital to the mortuary is covered**	☐1 Yes	☐0 No
K2.20	**Enough car parking at the mortuary**	☐1 Yes	☐0 No
K2.21	**Good access and exit routes for cars to stop congestion between arriving and departing groups**	☐1 Yes	☐0 No

Section L: Bereavement services

L1	Does the hospital have a bereavement service?	☐1 Yes	☐0 No

If yes, rate these statements about the bereavement service in this hospital.		Untrue 1	2	3	4	5	6	7	8	9	True 10
L2.1	Bereavement services are in a suitable part of the hospital	☐	☐	☐	☐	☐	☐	☐	☐	☐	☐
L2.2	Bereavement services are accessible without having to go through too many busy clinical areas	☐	☐	☐	☐	☐	☐	☐	☐	☐	☐
L2.3	Bereavement services counselling rooms have a calm atmosphere	☐	☐	☐	☐	☐	☐	☐	☐	☐	☐
L2.4	Bereavement services counselling rooms protect the confidentiality of the people using the service	☐	☐	☐	☐	☐	☐	☐	☐	☐	☐
L2.5	Bereavement services counselling rooms are child friendly	☐	☐	☐	☐	☐	☐	☐	☐	☐	☐
L2.6	Bereavement services counselling rooms are beside or have easy access to a washroom and toilet	☐	☐	☐	☐	☐	☐	☐	☐	☐	☐

Thank you very much for taking the time to complete this questionnaire.

Put it in the pre-paid H*f*H envelope and return it to the designated Hospital Audit Manager for posting to the H*f*H Programme.

Appendix 7

Letter of Invitation to Bereaved Relatives

Name of Bereaved Relative / Friend,
Address 1,
Address 2,
Address 3.

Date

Dear Bereaved Relative / Friend [Use Actual Name],

I would like to offer my sympathy on the recent death of your relative [actual name, RIP]. I hope you and your family are coping with the loss and distress that is inevitable at such a time.

I write specifically to ask if you would be willing to take part in a survey of bereaved relatives about the experiences of your late [mother, father, brother, sister, friend, etc] during the last week of [his / her] life in the hospital. The survey is being undertaken by the Irish Hospice Foundation as part of the Hospice Friendly Hospitals Programme, and our hospital is participating as a partner. The purpose of the survey is to find out about the care your relative experienced during the last week in hospital, so that we can learn from that experience and improve the quality of our care to patients and relatives.

I understand that this is a sensitive time for you. I also wish to inform you that your participation in the study is completely voluntary and that, if you do not feel that you can take part, you are completely free to do so. If you feel another relative is more appropriate to take part in the survey I would appreciate you passing this request on to them.

I would be very grateful if you could accept a telephone call from the hospital within the next week or so, to discuss if you are willing to participate in the postal survey and, if so, when would be a good time to send you the questionnaire. The questionnaire normally takes about half an hour to complete. A stamped-addressed envelope will be provided to return the completed questionnaire. Your answers to the questionnaire will be analysed by the Hospice Friendly Hospitals Programme and the results reported back to the hospital, but without revealing your identity. In this way, the survey is completely confidential and you can feel free to comment on any aspect of your relative's experience during the last week in hospital and how this affected you and your family.

Thank you for considering my request.

Yours sincerely

[Name], Director of Nursing.

Appendix 8

Briefing Notes for Phoning Relatives

Hospice friendly Hospitals PROGRAMME

Phoning Bereaved Relatives: Briefing Notes

Introduction

It can be difficult to make a telephone call to a person who is recently bereaved. However it is worth remembering that, in many bereaved people are very willing to give their opinions to hospitals for a number of reasons:

- To have a formal opportunity to give feedback to the hospital
- To ensure that their experiences might benefit others
- To help prevent negative experiences for others families
- To help to maximise positive experiences for other families
- To pay tribute to those who provided care to the patient.

Purpose of Phone Call

Your purpose in phoning the bereaved relative is to ask for their consent to participate in a survey of bereaved relatives. As you know, the survey of bereaved relatives is an integral part of the hospital's audit of end-of-life care. The survey of bereaved relatives, as described in the Manual, involves a four-step procedure comprising:

(i) sending a letter of invitation to inform bereaved relatives about the survey and seek their consent;

(ii) making a phone call to ascertain if consent is given or withheld;

(iii) sending an information pack containing the questionnaire and support materials on bereavement and bereavement services;

(iv) sending a thank you letter to all who took part in the survey while also reminding those who have not already done so, to returned their questionnaires as soon as possible.

These Briefing Notes are designed to help you with the second step of the procedure. They are intended to help you anticipate any difficulties, and to give you support.

Preparing for the telephone call

- Try to secure a quiet space or office from which to make the call.
- Ensure that you have the correct name of the next of kin and of the person who died and that you are clear how they were related (e.g., spouse, friend).
- Check that the letter of invitation to participate in the survey has already been sent out.

Talking to the person

- Clarify that you are talking to the right person.
- Give your name, explaining that you are telephoning from the hospital, on behalf of the Director of Nursing and following on from his/her recent letter.
- Clarify if this is a convenient time to talk further; if not decide on a mutually convenient time.
- Acknowledge in a sensitive manner the recent death of the named patient.
- Ask if they have had a chance to read the letter of invitation.
- Ask if they understand that the purpose of the survey is to find out about the care your relative experienced during the last week in hospital, so that the hospital can learn from that experience and improve the quality its care to patients and relatives.
- Ask if the relative/friend would be willing to receive a questionnaire, emphasising that while the hospital will use the information to improve its services, the individual information that the relative provides will be fully confidential.
- Explain that receiving the questionnaire does not commit them to returning it, they can make a decision on receiving it.

What if a person becomes upset or angry?

It is helpful to be aware that distress, anger and irritability are all common emotions in bereavement. It is possible that, on occasion, the person receiving the call will react in one of those ways.

- If a person is distressed you should acknowledge this distress, perhaps saying ‘I can tell this is a hard time for you’ or reiterating your condolences.
- If a person is angry remember that, however it may seem, the anger is not personal to you. Acknowledge the anger and, if it is appropriate, advise the person how to make an official complaint, or offer to have the Director of Nursing/Complaints Officer to contact them.
- You cannot change what has happened but you can provide a listening ear; that is why it is important to leave some time for the call.

Bereavement support

- Be aware of the hospital’s usual bereavement support procedure. If there is a bereavement service which will be in touch with the caller, let them know.
- If the relative is agreeing to receive the questionnaire, tell them that is will include some information on bereavement services.
- If the person would like to receive information on bereavement, please arrange to send the appropriate leaflets, even if they are not are agreeing to receive the questionnaire.

After the call

- Take a few minutes after the call to reflect on how it went. If it was a difficult call, remember that emotions can run high in bereavement and it is unlikely that you caused the anger/distress. It you feel upset or unsettled following a phone call, please debrief with a trusted colleague.
- Please make a note on the audit database of whether or not the person gave consent and, if so, ensure that the questionnaire and other enclosures are sent out within a couple of days.

Appendix 9

Letter with Questionnaire

Name of Bereaved Relative / Friend,
Address 1,
Address 2,
Address 3.

Date

Dear Bereaved Relative / Friend [Actual Name],

Thank you for agreeing to take part in the survey. I am now sending you the questionnaire and would like you to complete it whenever you can, preferably within the next week.

As I explained in my last letter, the survey is being undertaken by the Irish Hospice Foundation as part of the Hospice Friendly Hospitals Programme. The purpose of the survey is to find out about the care your relative experienced during the last week in hospital, so that we can learn from that experience and improve the quality our care to patients and relatives.

I would also like to repeat the assurance you that all of the information which you record on the questionnaire will be treated in absolute confidence, and the identity of you and your relative will never be revealed to anyone.

I hope you will be able to complete every question. However, if you find that some of the questions bring back memories that are upsetting or distressing, feel free to skip them and move on.

When you have completed the questionnaire, please put it in the stamped-addressed envelope which is also enclosed, and post it as soon as you can.

Finally, I enclose some information about bereavement which you may like to read, and some information about how to contact bereavement support services, if you need them. If you are having difficulty finding information, or if you need to talk to someone in confidence, please telephone the Irish Hospice Foundation at 01-679 3188 and we will do our best to help you.

Thank you again for agreeing to take part in the survey.

Yours sincerely

[Name], Director of Nursing.

Appendix 10

Leaflet on Bereavement

Bereavement

The death of someone close, for most of us, is the greatest loss we will suffer in our lives. Making sense of that loss and learning to live in the world without that person is the work of grieving.

The death of someone close may not be a surprise but it is very often a shock, because we cannot truly prepare ourselves for the loss of someone who is important in our lives. Everyone's loss is different and therefore everyone's grief is different.

In your grieving you may experience feelings such as shock, disbelief, confusion, sadness and loneliness. It is not uncommon to feel irritable and angry. The anger can be directed towards medical or emergency staff, God or even the person who died. Some people experience guilt and regrets about things they might have done or not done. There can also be a sense of relief, that the person's pain is over or that the difficult parts of caring are over.

Grief also has physical effects. You may find that your attention and concentration are poor, that you become absent-minded. You may have difficulty sleeping; you may have very vivid dreams when you do sleep. You may lose your appetite. Expect to feel out-of-sorts. Your body is trying to cope with the big changes in your life.

Many bereaved people find that at times their loss and grief overwhelm them. They think about what happened, cry and want to talk about the person who died. At other times their energy is taken up with day-to-day events and trying to get on with their lives.

Most people find their own way through the difficulties and pain of bereavement with support from friends and family.

Three levels of bereavement support:

1. Information

Many bereaved people find it helpful to learn more about bereavement and to read accounts of other people's experiences. The Irish Hospice Foundation has a number of leaflets which can be posted to you (phone 01 6793188) or if you have access to the web you can download them at www.hospice-foundation.ie. We have attached one with this questionnaire, the other leaflet titles are as follows:

When someone you care about is bereaved
- When a child is bereaved
- Adults grieving the death of a parent
- Grieving the death of someone close
- Living through the death of your partner or spouse
- The grieving family
- Grieving the death of a young child

2. Bereavement support

Some bereaved people find it useful to receive outside support from similarly bereaved people or from trained volunteers. Reasons for this might include having no supportive family members available or having concerns about burdening other family members.
Where can I get bereavement support?

There are several organisations you may wish to contact.

- Firstly, if your relative or friend was cared for by a hospice or palliative care team in the hospital or community, it is likely that a bereavement support service will be available through your contact there.
- If you have been bereaved through a hospital death, or through an accident where the person was dead on arrival at the hospital, it is likely that bereavement support will be available through the hospital's social work department
- The Bereavement Counselling Service (tel. 01-8391766) offers a volunteer-led bereavement support service mostly in Dublin
- If you have been bereaved through suicide, you can contact Console at 01-8574300 or the National Suicide Bereavement Support Network (tel. 024-95561).
- Finally, the Samaritans (1850 609090) offer a 24-hour listening service.

3. Bereavement counselling or therapy

A small minority of people experience significant difficulties in their bereavement. These might include: intense and unrelenting grief six months post-bereavement; prolonged agitation; depression; guilt; despair; or serious and persistent thoughts of suicide. If you are concerned, please consult your GP and consider professional help as unlike the more usual grief reactions, these symptoms do not always decrease over time.

Your GP or the hospital social worker could make a specific recommendation to you. Remember that professionally trained therapists will be registered with a governing body.

If you are having difficulty finding information please telephone the Irish Hospice Foundation at 01 6793188 and we will do our best to help you.

Appendix 11

Thank-you Letter to Bereaved Relatives

Name of Bereaved Relative / Friend,
Address 1,
Address 2,
Address 3.

Date

Dear Bereaved Relative / Friend [Actual Name],

Thank you again for agreeing to take part in the survey of bereaved relatives which is being undertaken by the Irish Hospice Foundation as part of the Hospice Friendly Hospitals Programme.

If you have already returned your questionnaire in the stamped-address envelope, thank you.

We value your opinions because they will help us to improve the quality our care to patients and relatives, especially those patients whose last days are spent in hospital.

If you have not yet had time to complete and return your questionnaire, I would appreciate if you could do so within the next few days. Your views and experience can make a difference.

Once again, I would like to assure you that all of the information which you record on the questionnaire will be treated in absolute confidence, and the identity of you and your relative will never be revealed to anyone.

Thank you again.

Yours sincerely

[Name], Director of Nursing.

Appendix 12

H*f*H Publication and Dissemination Strategy

Draft publication and dissemination strategy for the evaluation of the Hospice Friendly Hospitals Programme

David Clark

30 October 2007

Hospice Friendly Hospitals (H*f*H) is a programme designed to change hospital cultures of care and organisation relating to death, dying and bereavement in Ireland, using a 'systems level' approach. A national intervention on this scale poses significant evaluation challenges. To that end the National Steering Committee of H*f*H has established an Evaluation Sub-Committee, charged with overseeing the evaluation and ensuring that the H*f*H evaluation requirements are fully met.

The Evaluation Sub-Committee is responsible for ensuring that the evaluation and any results it generates are appropriately disseminated and made available to a wider audience. Within the terms of reference of the Evaluation Sub-Committee is the requirement to advise on and review publication plans for each evaluation component of the H*f*H Programme and on publications related to the preceding pilot project.

The following are suggested as good principles.

1. It is important that outputs from the evaluation are widely disseminated and appear in a variety of journals and other publications. Some significant 'summative' papers are expected to appear in high impact factor, peer reviewed journals. It is also important that evaluation results are published in professional journals, magazines, newsletters, conference symposia etc, even though these may not be peer reviewed.

2. All abstracts, submissions for publication, and major conference presentations relating to the evaluation of H*f*H should be notified to the Evaluation Sub-Committee through its chair and in advance of submission or presentation. Efforts will be made to seek to assure the quality of such items and in doing this will draw on the expertise of the Evaluation Co-ordinator and other members of the Evaluation Sub-Committee, as required. Where work has been sub-contracted to academic providers, it may not be reasonable for the above to apply, but written agreement with these providers should at least include the requirement to inform the Sub-Committee chair of such work, prior to submission or presentation. A form of words which clearly establishes the work as part of the H*f*H programme of activities will be drafted for use in all such publications and authors of evaluation outputs will be encouraged to use it.

3. Authorship of these outputs should reflect the key persons involved and should be inclusive in orientation – whilst at the same time avoiding a tendency to include names of those merely tangentially involved. Evaluation outputs should seek to involve authorship by H*f*H programme staff, wherever appropriate and where they are able to make a recognised contribution to the work.

4. It is important that any other publications, public domain outputs or similar relating to the H*f*H programme (but not involving its evaluation) are notified to the Evaluation Co-ordinator *for information*, even though they do not comprise outputs of the evaluation.

5. It is suggested that H*f*H may benefit from a wider strategy for *dissemination* of its work, though this clearly goes beyond the remit of the Evaluation Sub-Committee. The dissemination strategy should cover such areas as policy briefings, press releases, newsletters, seminars, conferences, campaigns, etc. Close linkage between the publication of evaluation findings and the wider dissemination of information would be desirable.
