# Supplementary material for: Determinants of care outcomes for patients who die in hospital in Ireland: a retrospective study
Source: BMC Palliat Care. 2015 Apr 18;14:11. doi: 10.1186/s12904-015-0014-2 (PMC4422526; doi:10.1186/s12904-015-0014-2)
Supplement: Additional file 2: — Statistically significant (p < .05) relationships between care inputs and care outcomes. [file 12904_2015_14_MOESM2_ESM.docx]

**Additional file 2**

**Statistically significant (p < .05) relationships between care inputs and care outcomes**

| Care Inputs | Level | Symptom Manage | Symptom  Experience | Patient  Care | Accept-ability | Family Support |
| --- | --- | --- | --- | --- | --- | --- |
| Disease & Sudden Death: |  |  | | | | |
| *Cancer (0/1) (the reference is circulatory disease)* | L1 |  |  | 3.45D |  |  |
| *Respiratory disease (0/1) (the reference is circulatory disease)* | L1 |  |  | -3.16N |  |  |
| *Dementia (0/1) (the reference is circulatory disease)* | L1 |  |  | -5.01N |  |  |
| *Sudden Death (0/1)* *(the reference is expected death)* | L1 |  | -4.46N  -6.94R | -14.57R |  |  |
| Route of admission: |  |  | | | | |
| *Admission not through ED (0/1)* | L1 |  | 5.11N |  |  | 3.64R |
| *Elective admission (0/1)* | L1 | 4.22D |  |  | 5.63N 4.13D |  |
| Physical environment: |  |  | | | | |
| *Single rooms (0/1)* | L1 | 4.21D | 7.66R |  | 5.67N 5.09R |  |
| *Dignity of ward / room (1-100)* | L1 | 0.09N  -0.06D |  | 0.1N | 0.14N |  |
| *Environment of ward (1-100)* | L1 |  |  | 0.08N 0.12D |  |  |
| Team meetings: |  |  | | | | |
| *Medical & nursing team (0/1)* | L1 |  |  | 4.91N 3.49D |  | 2.68N |
| *Multidisciplinary team (0/1)* | L1 | 5.22N |  |  |  |  |

| Communication: |  |  | | | | |
| --- | --- | --- | --- | --- | --- | --- |
| *Discussion with patients (0-100)* | L1 |  | 0.04N | 0.06N |  | -0.07N |
| *Discussion with relatives (0-100)* | L1 | 0.15N |  | 0.12N  0.09R | 0.09N | 0.08N  0.05R |
| Facilitating relatives: |  |  | | | | |
| *Relatives stayed overnight (0/1)* | L1 | 3.84N |  |  |  |  |
| *Relatives present at death (0/1)* | L1 |  |  |  | 5.0N  4.98R |  |
| Nurse readiness: |  |  | | | | |
| *Professionally prepared (0/1)* | L1 |  | 4.14N  6.75R |  |  |  |
| *Personally prepared (0/1)* | L1 |  |  |  | 4.42N |  |
| *End-of-life training (0/1)* | L1 | 5.92D |  |  |  |  |
| *Years in ward (0-100)* | L2 |  | 0.46N  1.34R |  |  |  |
| *Years in hospital (0-100)* | L1 |  |  |  | 3.69R | 0.91N |
| Hospital governance: |  |  | | | | |
| *Goals in business plan (0/1)* | L3 | 4.89D |  |  |  |  |
| *Sufficiency of ward staff (0-100)* | L2 | -0.06N  -0.09R |  |  |  |  |

Notes:

N=Nurse. D=Doctor. R=Relative. Results are unstandardised regression coefficients.

L1=Level 1 (individuals); L2=Level 2 (wards); L3=Level 3 (hospitals).

Dichotomous variables (0/1) variables should be interpreted as in the following example: the coefficient 3.45 for the effect of being a cancer patient on doctors' assessment of patient care implies that patient care is 3.45 percentage points higher for those whose primary diagnosis is cancer (controlling for other variables).

Scale variables (0-100) should be interpreted as in the following example: the coefficient 0.08 for the effect of the environment of the ward / room on nurse’s assessment of patient care implies that patient care is 0.08 percentage points higher for each percentage point improvement in the environment of the ward / room; this implies that even a small coefficient such as 0.08 can make an appreciable difference where the percentage point difference is 10, 20 or more.
